# High-performance polymer 3D printing – Open-source liquid cooled scalable printer design

**DOI:** 10.1016/j.ohx.2022.e00265

**Published:** 2022-01-10

**Authors:** Andreas Hagerup Birkelid, Sindre W. Eikevåg, Christer W. Elverum, Martin Steinert

**Affiliations:** a Department of Mechanical and Industrial Engineering, Norwegian University of Science and Technology, Trondheim, Norway; b Department of Civil and Environmental Engineering, Center for Sports Facilities and Technology, Norwegian University of Science and Technology, Trondheim, Norway

**Keywords:** Open source, Open hardware, High-temperature 3-D printing, Additive manufacturing, Polymide, PEEK

## Abstract

To print high-performance polymers, a stable running printer that can reach high temperatures is needed. There is currently a lack of low-cost solutions that allow manipulation of process parameters and expansion of sensors to monitor the printer as well as the process. This paper presents an open-source hardware upgrade for low-cost 3D printers to enable research on new high-temperature polymers as well as manufacturing from all currently available polymers. The hardware cost less than $1700, including the printer. Open-source firmware by Klipper and Fluidd is used for control. The printer is able to reach 500 °C nozzle, 200 °C heated bed, and 135 °C heated chamber with all electronics inside operating within the recommended temperature range. The presented design produced a CF-PEEK 3DBenchy and a spiral vase with excellent surface quality and no signs of delamination. Test specimens according to ISO527 using PA-CF performed similarly to the datasheet provided by the manufacturer for samples produced in the XY-orientation and outperformed the datasheet by 15 % in the ZX direction. Compared to specimens made on an Original Prusa i3 MK3S, the modified printer produced specimens with 22% higher strength in the YX-direction and 25% in ZX. By continuously monitoring and carefully calibrating both hardware and firmware, the presented design can perform as a research tool in material science and produce large-scale components of high-performance polymers.

## Graphical abstract

**Figure:**
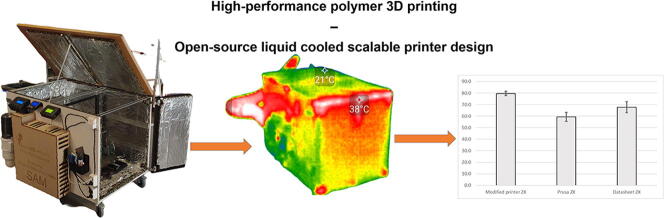

## Specifications table

**Table:** 

Hardware name	Open-Source Liquid-Cooled High-Performance 3D-Printer
Subject area	• Engineering and Material Science Educational Tools and Open Source Alternatives to Existing Infrastructure
Hardware type	• Additive manufacturing
Open Source License	CC by 4.0
Cost of Hardware	1700$
Source File Repository	https://doi.org/10.17632/7sjjmr9bz2.1

## Hardware in context

Fused Filament Fabrication (FFF) is a process where thermoplastic filament is extruded through a heated nozzle and parts are built layer by layer. Compared to other additive manufacturing (AM) processes, FFF is typically a less expensive and easy-to-operate alternative [1]. The process was first commercialized and trademarked as fused deposition modeling (FDM) by Stratasys in 1991. Up until 2009, Stratasys was the only company offering these machines due to their patent [2]. After the patent expired in 2009, RepRap [3] was the first open-source project that spurred an entire community of developers and eventually lead to the emergence of several companies, such as Prusa Research, LulzBot and Ultimaker. Common materials used for these machines are low-cost commodity and engineering polymers, such as acrylonitrile butadiene styrene (ABS), polylactide (PLA) and polyethylene terephthalate (PET) [4].

The evolvement and releases of new material blends such as carbon-fiber (CF) reinforced nylon (PA-CF), and polyether ether ketone (CF-PEEK) enable printed parts with higher strength than materials traditionally used in FFF [5]. However, these materials require high printing temperatures compared to entry-level materials [6] and a high environment temperature during printing [7], [8], [9]. In addition, high-performance materials can absorb humidity from the air, introducing defects and decreasing the achievable strength [10] and surface tolerances without proper humidity control. Due to recent development, FFF can now meet the strength and temperature resistance required for industrial applications; however, additional understanding between the FFF process and the materials must be investigated before scientific breakthroughs can happen [5]. In developing new polymer composites, open-source hardware for filament creation enables researchers to modify new high-performance materials [11]. However, the lack of availability of high-temperature additive manufacturing machines limits the research opportunities, but some are available for a relatively high cost [5]. For example, Intamsys Funmat HT enhanced has a maximum printing temperature of 470 °C, a heated bed up to 200 °C, and a heated environment that can reach 90 °C [12]. However, it has a relatively small build volume of 260 mm × 260 mm × 260 mm and comes at a price of almost 7500$. On the other hand, AON M2 has a larger build volume of 454 mm × 454 mm × 640 mm and can reach a chamber temperature of 120 °C, but the price is almost 50.000$ [13].

Various open-source printer designs with limitations are available to overcome the high price entry of high-performance 3D printing. For example, in 2016, NASA investigated using low-cost open-source hardware [14] to print ULTEM 1010 polyetherimide(PEI). By replacing the extruder and building an enclosure using IR lamps as an additional heat source, NASA managed to print parts using PEI filament with no evidence of warping or delamination. A more recent study [6], using an inverted build chamber with a heated bed and heated walls, maintained a chamber temperature of 200 °C, until 29.8 mm from the build plate. By printing polyphenylsulfone (PPSF) at this temperature, they managed to increase the specimen’s ultimate tensile strength by 48%. In a study where the focus was on printing heat-sterilizable personal protective equipment, the authors developed an open-source 3D printer and demonstrated the ability to print polyetherketoneketone (PEKK) and PEI/Ultem [15].

This article presents an open-source printer and environment design to enable further research and improvements of high-performance materials. By thermally protecting sensitive components, some of the drawbacks from previously proposed designs are solved [6]. The proposed design distinguishes itself from the aforementioned open-source designs by being:

• a modification of existing printers rather than a custom-built machine, utilizing off-the-shelf components where possible
• a highly scalable design, suitable for producing larger components
• a flexible design platform accommodating additional sensor equipment to monitor and control parameters needed to conduct research [5]

Furthermore, the environment can reach high temperatures in a controlled manner for optimal layer adhesion and minimal shrinkage. The filament path is short to enable accurate material extrusion and good printability of carbon-filled materials [15]. The filament is placed in a heated and controlled storage during printing to monitor and protect materials from humidity. Taken together, the open-source design proposed here aims to provide an alternative for those who want to upgrade their existing printer to manufacture high-performance polymer components for research or end-use applications. Ultimately, contributing to the progress of distributed manufacturing.

## Hardware description

**Fig. 1:**
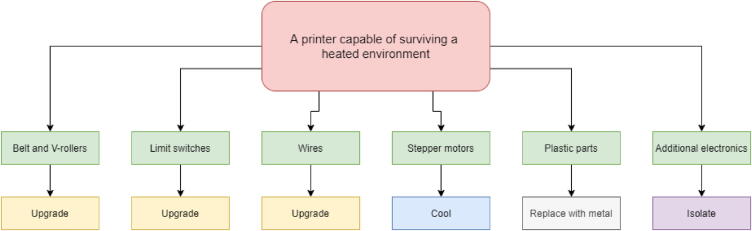
Low-cost 3D-printer capable of surviving a heated environment.

The liquid-cooled high-performance 3D printer presented in this build is a concept that can make virtually any low-cost 3D printer capable of printing high-performance materials. The price can be kept low by upgrading components on an already existing printer. The concept is as follows (as illustrated in Fig. 1):

• Build a temperature-controlled heated chamber.
• Remove, upgrade, or cool all temperature-sensitive objects.
• Increase the possible nozzle and bed temperature.
• Keep filament dry during printing.
• Utilize compatible software.
• Add additional sensor systems.

**Fig. 2:**
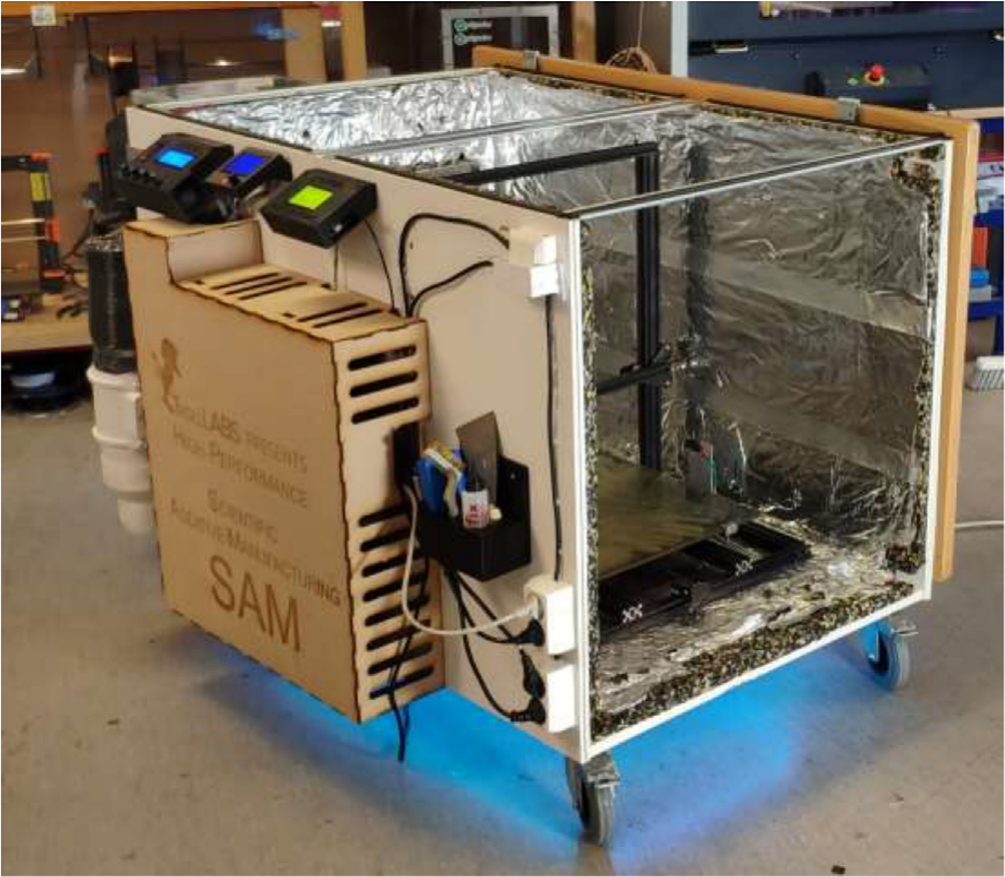
Presented printer design.

The instructions include the procedure of building a temperature-controlled chamber that is capable of high temperatures, ensuring good layer adhesion, and minimal warping during printing of high-temperature materials. The upgraded hotend is capable of temperatures up to 500 °C, and a silicone bed heater enables up to 200 °C and ensures good adhesion while also working as a second heater for the chamber. In addition, the filament is continuously dried during printing to decrease the humidity inside the material and improve printing quality. Fig. 2

All stepper motors are liquid-cooled, enabling upgrade of most commercially available printers and not only limited to printers where the stepper motors are isolated from the heated chamber. In addition, the design implements a liquid-cooled direct drive extruder which ensures a short filament path and accurate extrusions.

The liquid-cooled 3D printer presented in this paper has the following features:

• 135 °C chamber with both heating and cooling.
• High temperature liquid-cooled direct drive extruder and AC powered heated bed.
• Large build volume of 500 mm × 500 mm × 500 mm.
• Heated filament storage and drying chamber up to 90 °C.
• Possibility and room for adding monitoring systems and additional sensors for material research.

## Safety and reliability

The high-performance 3D printer build and components require familiarity with 3D Printers, soldering, wood- and metalworking. Also, knowledge of electronics is important, both AC and DC power systems, as wiring AC can be dangerous if done incorrectly. Ensure that all electrical connections are correctly connected and that conductive surfaces are grounded. The builder and user should wear proper personal protective equipment, such as safety glasses, gloves, and coats. Experienced personnel should be contacted if the country requires specific certification or if the builder lacks the skills to build the system properly. Further, the printer and its environment are built using low-cost components and are working at high temperatures. The risk of fire increases with temperature, and it is very important never to leave the printer unattended while it is running. The printer should only be used in a fire-safe space with a fire extinguisher close by. Printer temperatures and cooling systems should be monitored continually while operating. Some high-temperature filaments may emit dangerous fumes when heated to sufficient temperatures. Therefore, the printer should be used in ventilated space to capture chemical emissions.

The printer is built using low-cost solutions; therefore, some components may be checked and replaced regularly. This includes, but is not limited to, heaters, limit switches, belts, silicone tubes, and water pumps. Also, the aluminum profiles may be rotated or replaced by stainless steel profiles as the stainless-steel rollers will create grooves over time. Software errors and board freezing might also happen, which in most cases is solved by a hard restart, software update, or a board replacement.

As of when this article is published, the presented printer has been operational for one year and six months with approximately 500 h of running time without any major issues. Until now, one hotend thermistor has been replaced, and after approximately 500 h, we started to see wear of the aluminum profiles and addressed the issue in a simple operation by rotating the profile 90°. As our presented design are mounted on wheels, the printer has also been transported between several locations within the university without any signs of structural damage. The printer still provides calibration prints with high accuracy, just as when it first was put in operation.

## Design files

**Table:** 

Design file name	Number of files	File type	Open source license	Location of the file
*CableStrainRelief*	*2*	*STEP, STL*	*CC BY 4.0*	https://doi.org/10.17632/7sjjmr9bz2.1
*Chamber Heater*	5	DXF, PDF, STEP, STL	*CC BY 4.0*	https://doi.org/10.17632/7sjjmr9bz2.1
CoolingControl	5	STEP, STL	*CC BY 4.0*	https://doi.org/10.17632/7sjjmr9bz2.1
Drying Chamber	16	DXF, PDF, STEP, STL	*CC BY 4.0*	https://doi.org/10.17632/7sjjmr9bz2.1
Electronics Protection	9	DXF, STEP, STL	*CC BY 4.0*	https://doi.org/10.17632/7sjjmr9bz2.1
ExhaustFan	2	STEP, STL	*CC BY 4.0*	https://doi.org/10.17632/7sjjmr9bz2.1
ExtruderMount	7	DXF, STEP, STL, PDF	*CC BY 4.0*	https://doi.org/10.17632/7sjjmr9bz2.1
FanAdapters (from thingyverse)	3	STL	*CC BY 4.0*	https://doi.org/10.17632/7sjjmr9bz2.1
HeatedChamber	3	STEP, PDF	*CC BY 4.0*	https://doi.org/10.17632/7sjjmr9bz2.1
RadiatorHolder	9	DXF, STEP	*CC BY 4.0*	https://doi.org/10.17632/7sjjmr9bz2.1
ReservoirHolder	10	DXF, STEP	*CC BY 4.0*	https://doi.org/10.17632/7sjjmr9bz2.1
ReservoirLids	9	DXF, STEP, STL	*CC BY 4.0*	https://doi.org/10.17632/7sjjmr9bz2.1
ThermistorMeasurement	5	STEP, STL	*CC BY 4.0*	https://doi.org/10.17632/7sjjmr9bz2.1
XcarriageBracket	4	DXF, PDF, STEP, STL	*CC BY 4.0*	https://doi.org/10.17632/7sjjmr9bz2.1
ZlimitSwitch	3	PDF, STEP, STL	*CC BY 4.0*	https://doi.org/10.17632/7sjjmr9bz2.1
ZmotorHolder	3	PDF, STEP, STL	*CC BY 4.0*	https://doi.org/10.17632/7sjjmr9bz2.1

## Bill of materials

**Table:** 

Design file name	File type	Open source license	Location of the file
Drying Chamber	.xlsx	CC BY 4.0	https://doi.org/10.17632/7sjjmr9bz2.1
Extras	.xlsx	CC BY 4.0	https://doi.org/10.17632/7sjjmr9bz2.1
Heated Chamber	.xlsx	CC BY 4.0	https://doi.org/10.17632/7sjjmr9bz2.1
LiquidCooling	.xlsx	CC BY 4.0	https://doi.org/10.17632/7sjjmr9bz2.1
Printer Mods	.xlsx	CC BY 4.0	https://doi.org/10.17632/7sjjmr9bz2.1

## Code, Klipper configuration and slicer profile

**Table:** 

Design file name	File type	Open source license	Location of the file
*DryingChamber*	*INO*	CC BY 4.0	https://doi.org/10.17632/7sjjmr9bz2.1
*LiquidTemp*	*INO*	CC BY 4.0	https://doi.org/10.17632/7sjjmr9bz2.1
*StepperTemp*	*INO*	CC BY 4.0	https://doi.org/10.17632/7sjjmr9bz2.1
*Klipper-Config-Baseline*	*CFG*	CC BY 4.0	https://doi.org/10.17632/7sjjmr9bz2.1
*Klipper-Config-Extra*	*CFG*	CC BY 4.0	https://doi.org/10.17632/7sjjmr9bz2.1
*SuperSlicer_config_bundle*	*INI*	CC BY 4.0	https://doi.org/10.17632/7sjjmr9bz2.1

## Build instructions

The high-performance 3D printer build and its components require familiarity with 3D printers, wood- and metalworking, soldering, and electronics. The authors chose to use hardware and tools available at their workshop; however, different tools than noted should be possible to use with ease. The build instructions are to be read thoroughly before attempting the build. As the build is quite extensive, small operations may be omitted, and it is assumed that the builder is experienced to manage without detailed instructions. Several screw sizes and material choices should be suitable for this build. It is intended that the builder choose whatever he/she has in their workshop in both materials and components. If the printer used in this build is the only available printer in the workshop, ensure that all parts are printed before disassembling the printer. All woodscrews are of size 3.5 mm × 20 mm unless stated otherwise. Where printed material is marked as a heat-resistant filament (HF), it is recommended to use either HTPLA or PolyCarbonate with 100% infill and annealing the parts according to manufacturer recommendations. One could choose any filament and infill percentage for other non-high-temp objects, but a general guideline would be PLA with 15% infill and supports enabled. The length and connection of wires may vary depending on the mounting position. It is recommended to take a quick measure of the required length and add some extra to provide slack. Several electrical connections may be used to complete this build.

### Building an enclosed environment

**Fig. 3:**
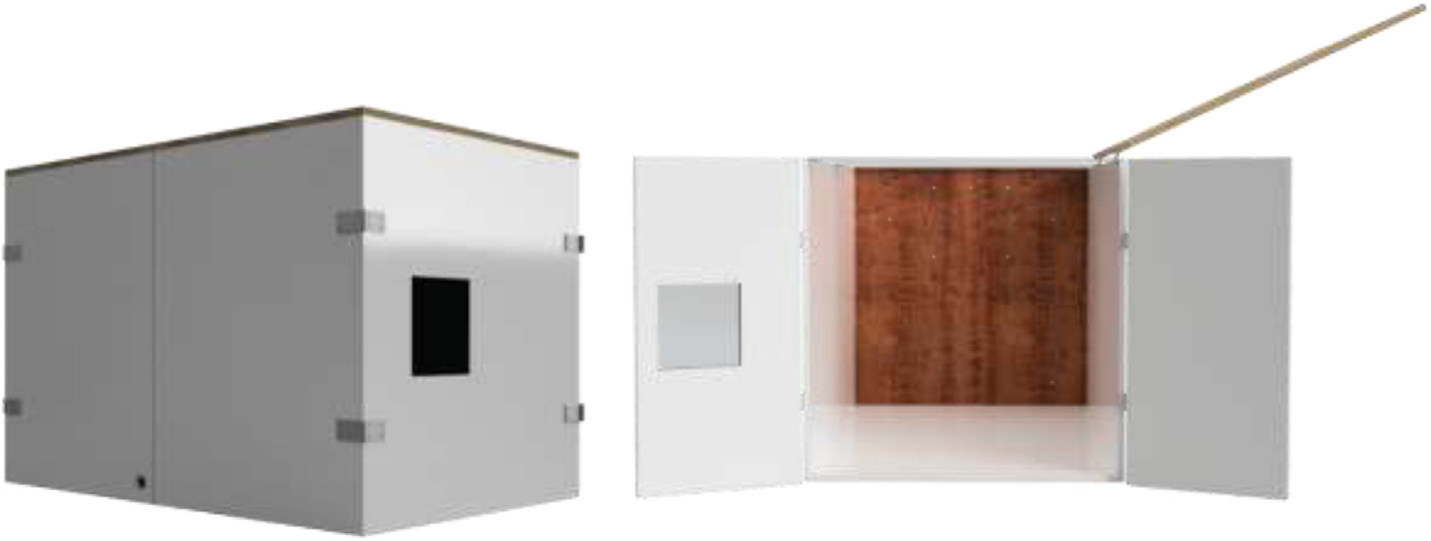
Enclosed environment.

The enclosed environment presented by the authors is created using two interconnected 800x600x800mm IKEA cabinets for simplicity. Two doors, a backplate, and a top cover are also installed. When finished building, the environment could look as shown in Fig. 3 with a total dimension of 800x1200x800.

**Fig. 4:**
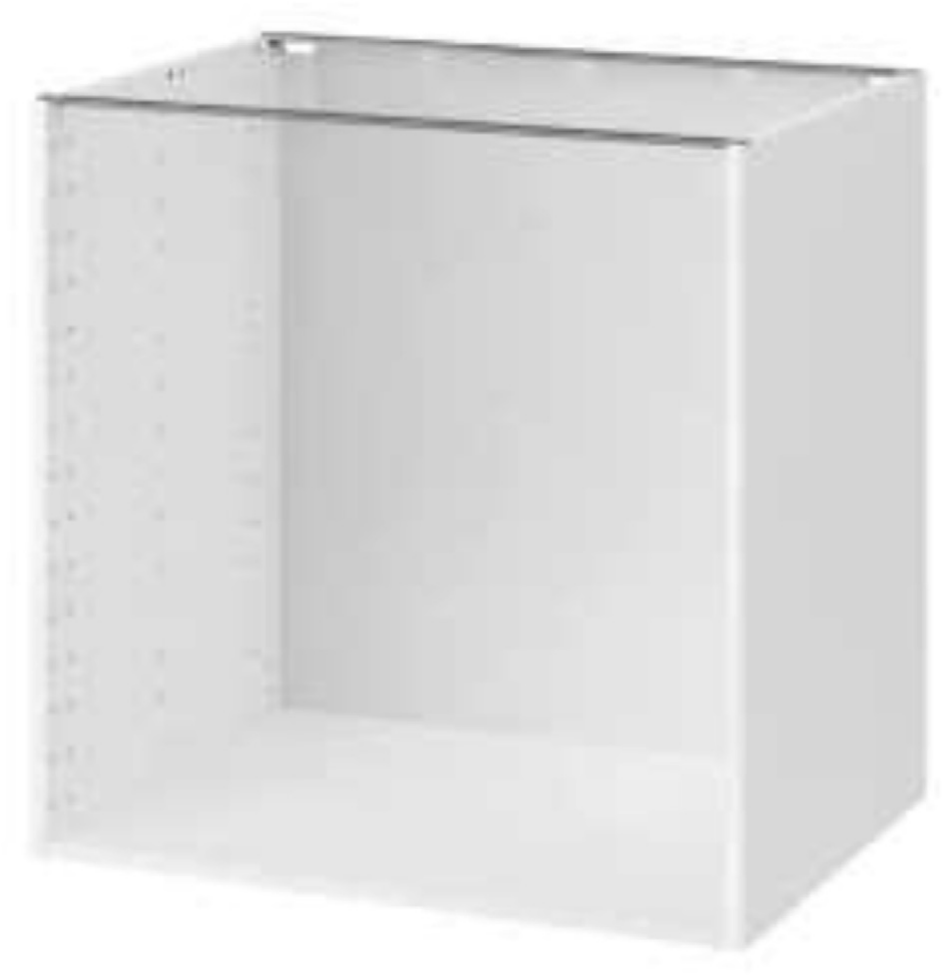
IKEA METOD assembled [16]

Start by building both closets using the provided instructions from IKEA as in Fig. 4. The backplate can be omitted as it is not needed.

**Fig. 5:**
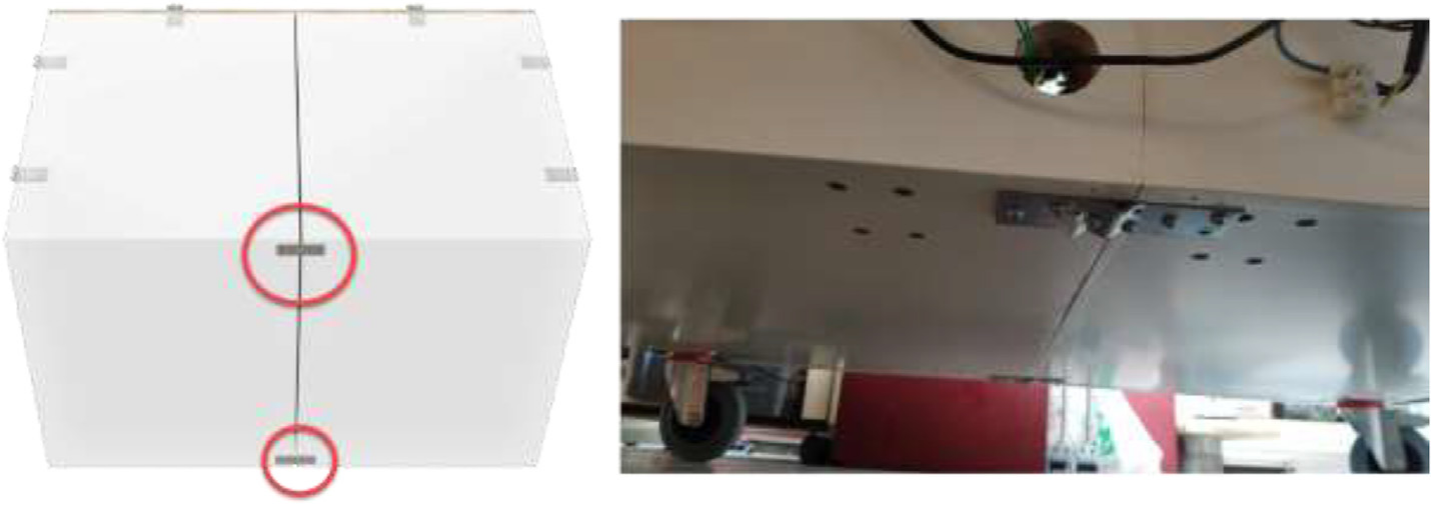
Interconnection of two closets.

Connect both closets using steel plates in the bottom (Fig. 5) and seal the cabinets using construction adhesive. Optionally, attach wheels to each bottom corner to ease transportation. If the printer is to be transported frequently, additional steel plates or L-beams may be required to strengthen the chamber.

**Fig. 6:**
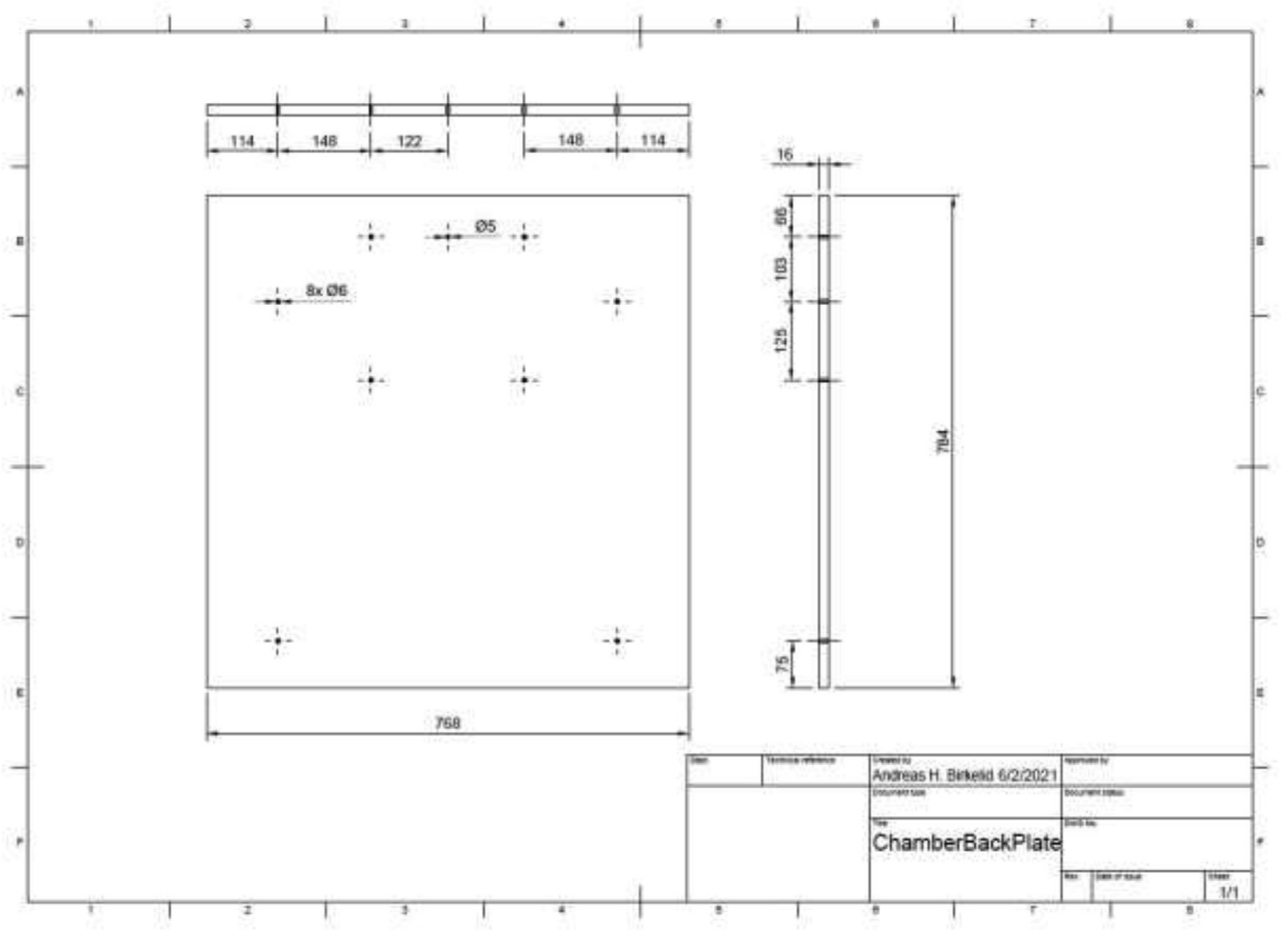
Chamber backplate.

Cut and drill a ∼ 16 mm thick wood plate to be used as a backplate according to the dimensions in Fig. 6.

**Fig. 7:**
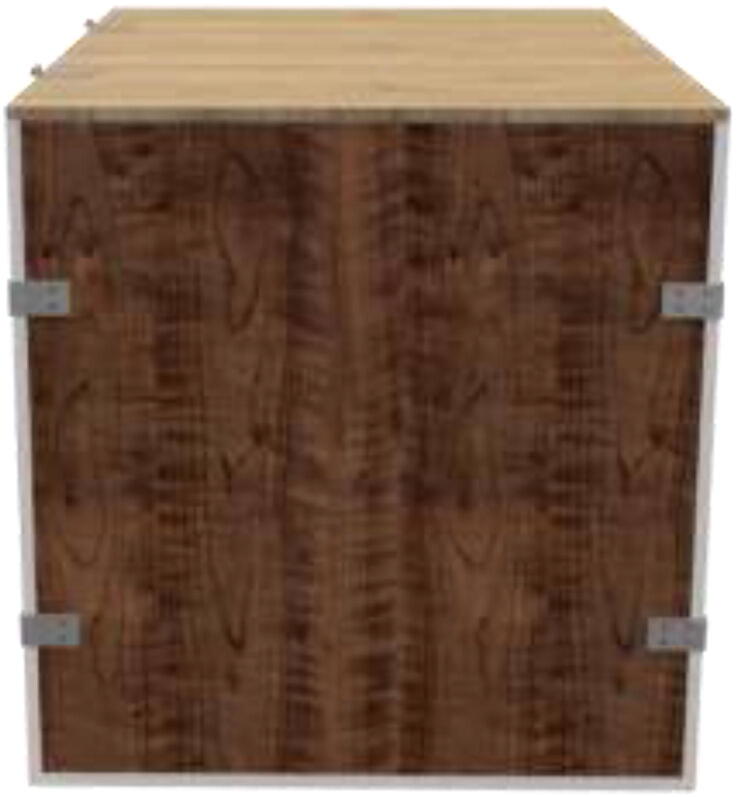
Mounting position of the backplate.

Add backplate to the chamber using wood screws and four angle brackets (Fig. 7). Make sure that it is mounted in the same orientation as presented in (Fig. 6).

**Fig. 8:**
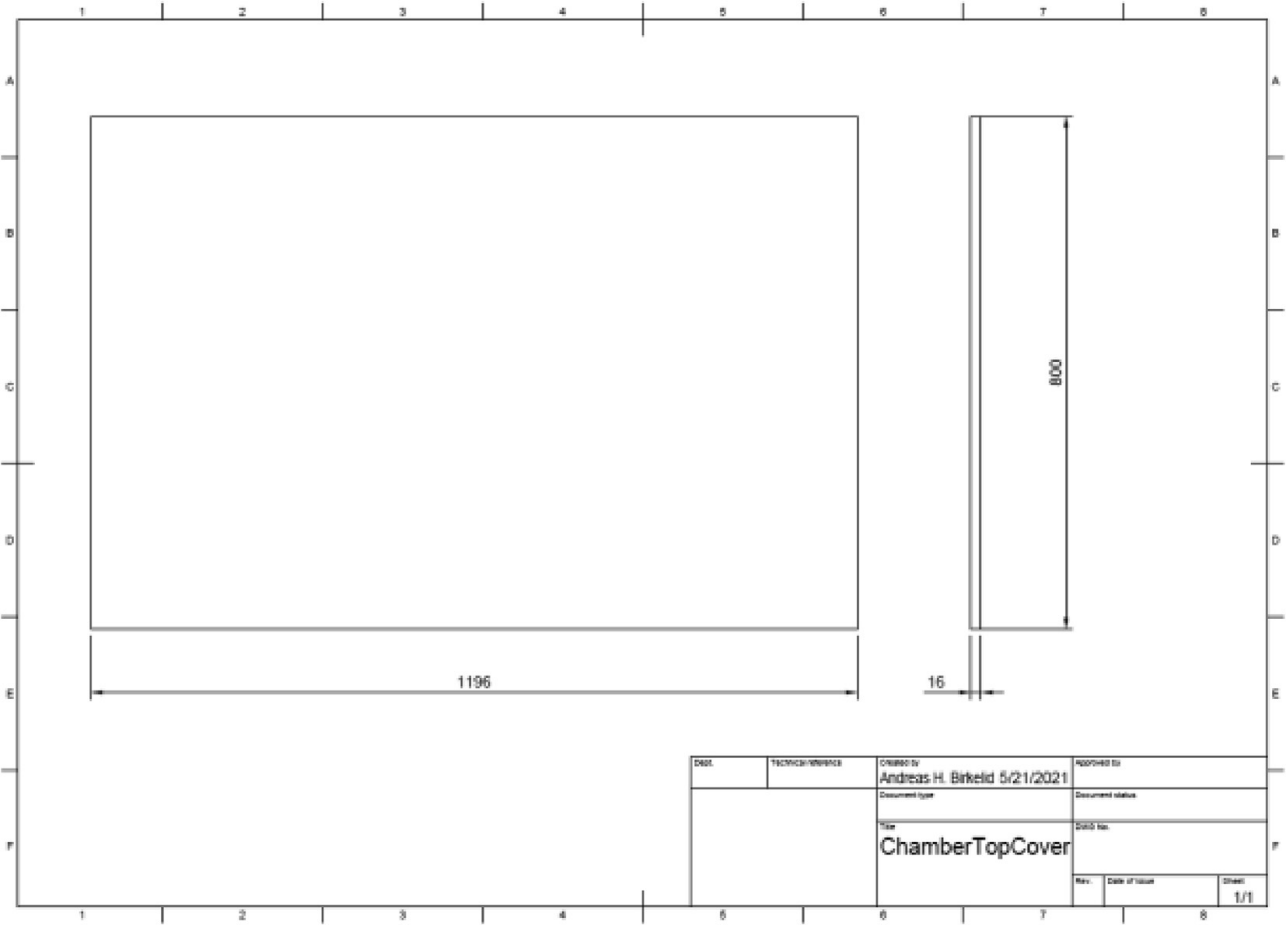
Top cover.

Cut a ∼ 16 mm thick wood plate functioning as a top cover into the dimensions presented in Fig. 8.

**Fig. 9:**
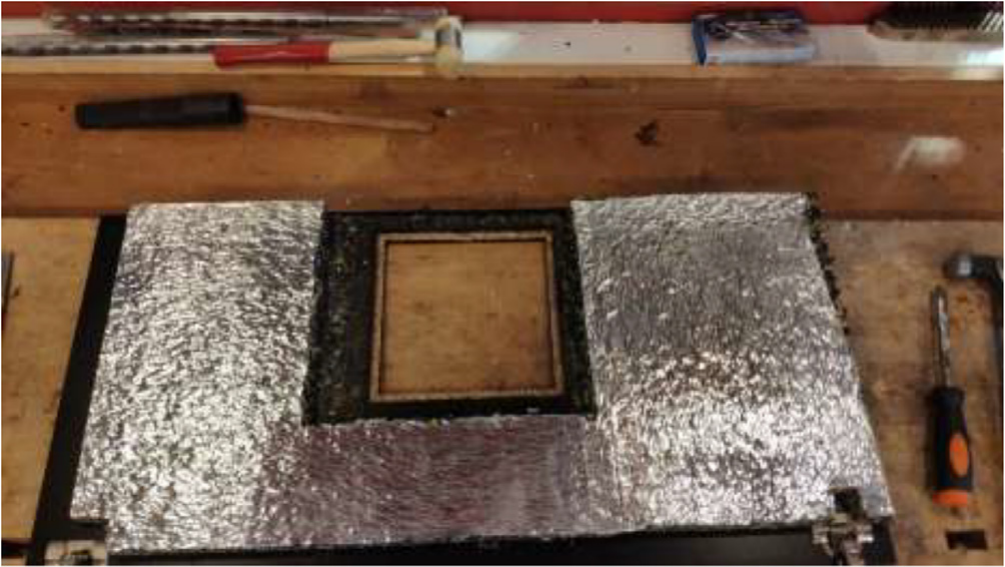
Optionally, window installation.

Optionally, the user can cut a square hole through one of the front doors and insert a heat-resistant glass plate with a 200 mm × 200 mm size as a window. Then, seal the glass using high-temperature silicone (HTS) (Fig. 9).

**Fig. 10:**
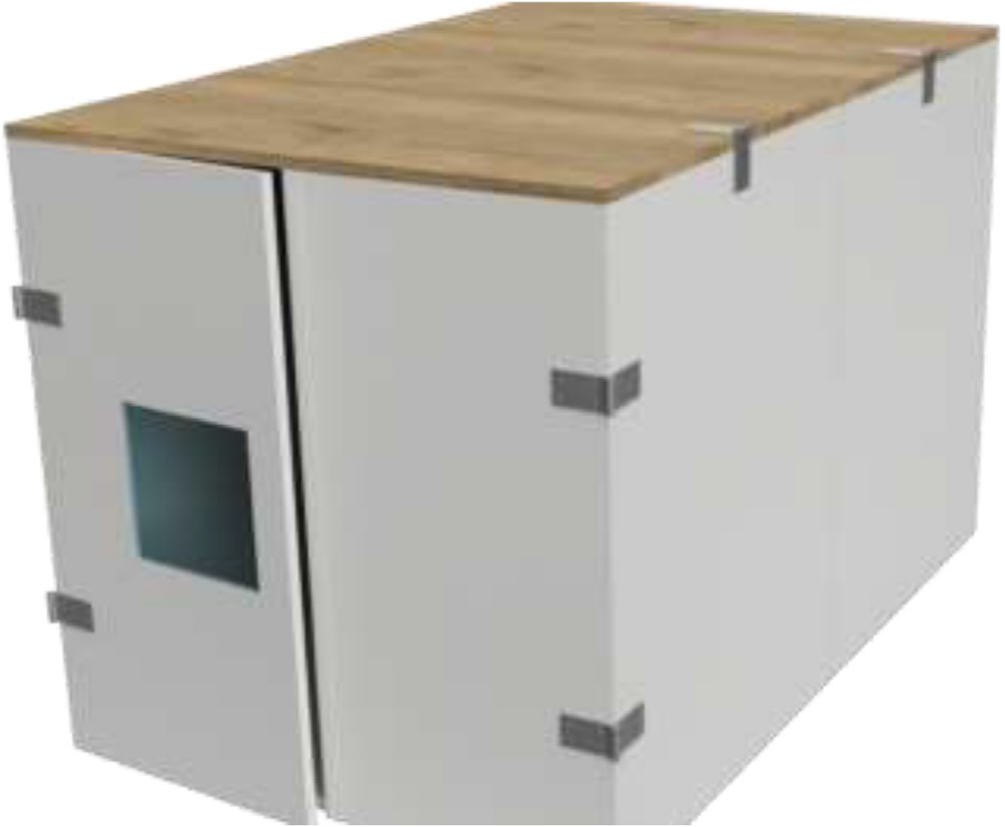
Hinge placement for doors and top cover.

Assemble the top plate and doors using two hinges on each component and fasten using wood screws (Fig. 10). Make sure that the cover and doors can be closed tightly.

**Fig. 11:**
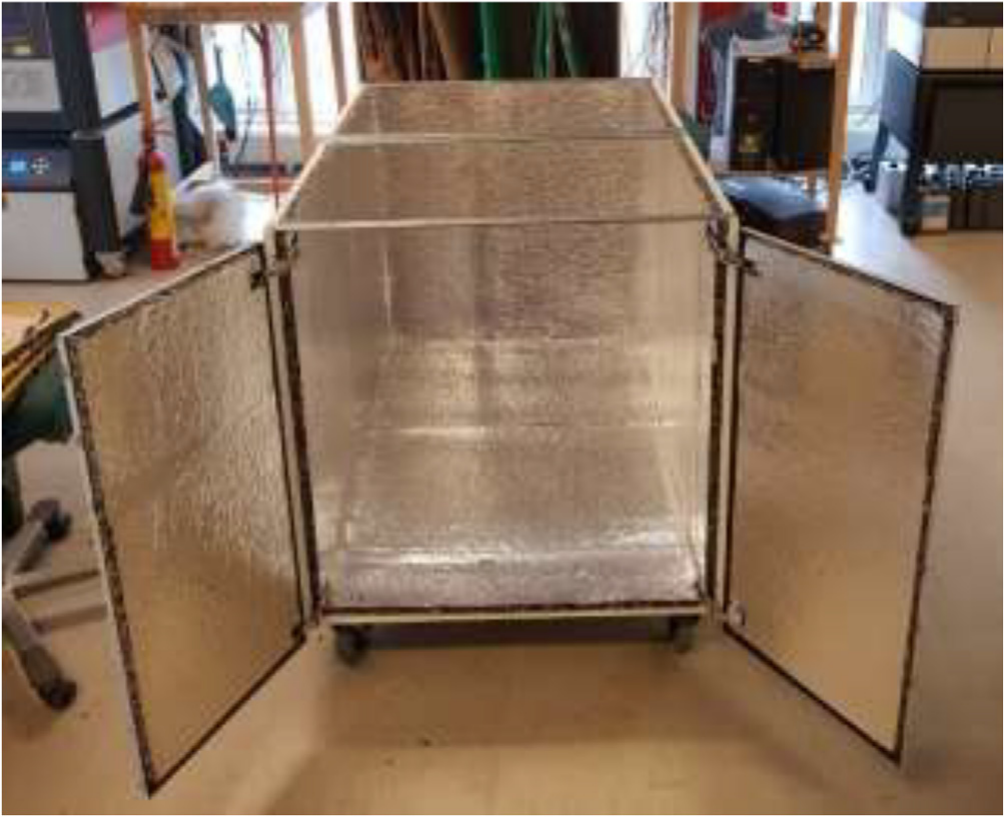
Insulation of heated chamber.

Insulate the chamber using ∼ 20 mm car heat insulation mat as noted in the repository, and seal all gaps using aluminum tape. The insulation is cut to the preferred size and glued to walls, as seen in Fig. 11. Make sure that doors and top cover still form a tight seal when closed.

**Fig. 12:**
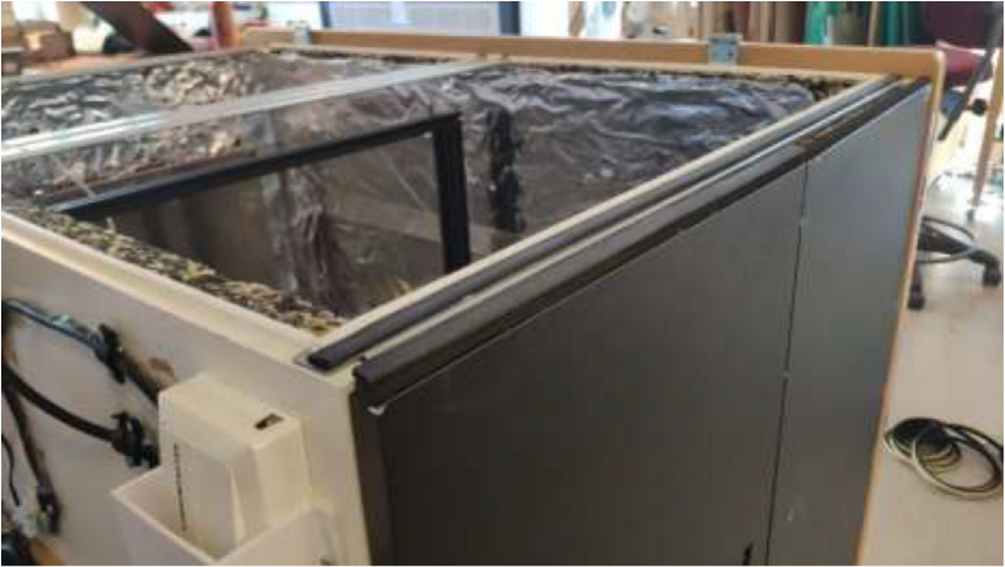
Rubber gaskets example configuration.

Add rubber gaskets around edges to seal, following Fig. 12.

**Fig. 13:**
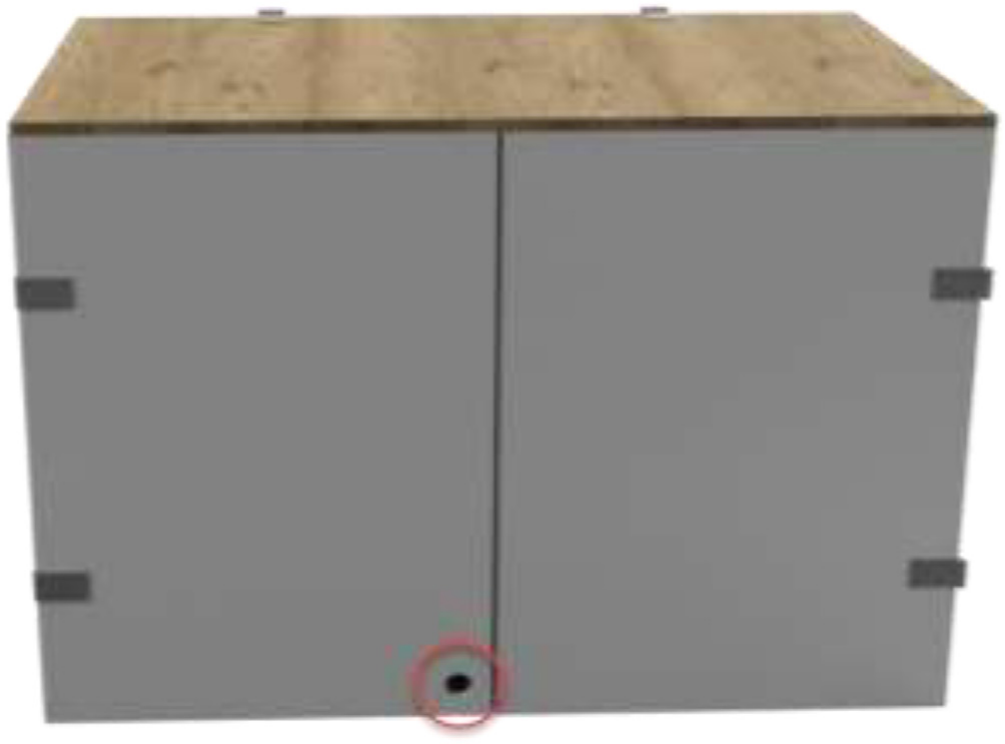
Hole for cables from 3D Printer.

The cables are routed from inside the heated environment through a hole made on the left side (Fig. 13). Proceed with drilling a 30 mm hole in the bottom middle on the left side, as seen in Fig. 13. Ensure that the hole is positioned 30 mm from the bottom edge to avoid damaging the bottom plate.

**Fig. 14:**
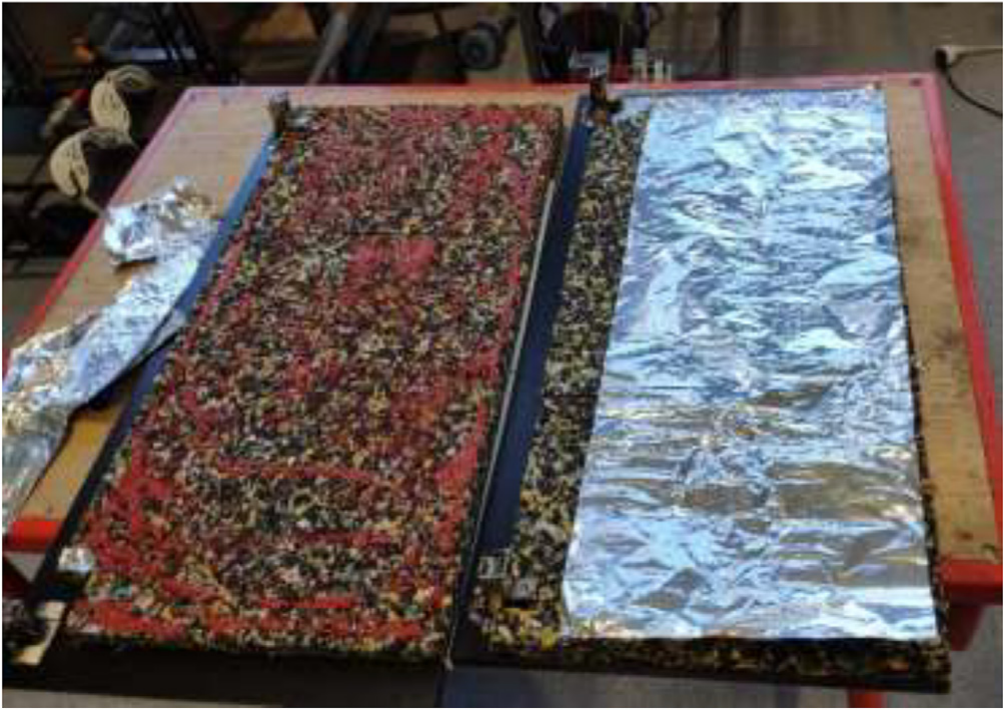
Optionally, replacing reflective foil with aluminum foil.

The glue for the reflective foil may not be rated for high temperatures depending on the chosen insulation. If the foil loosens, one could remove it entirely, add HTS, cut aluminum foil to the desired size, and fix it as in Fig. 14.

**Fig. 15:**
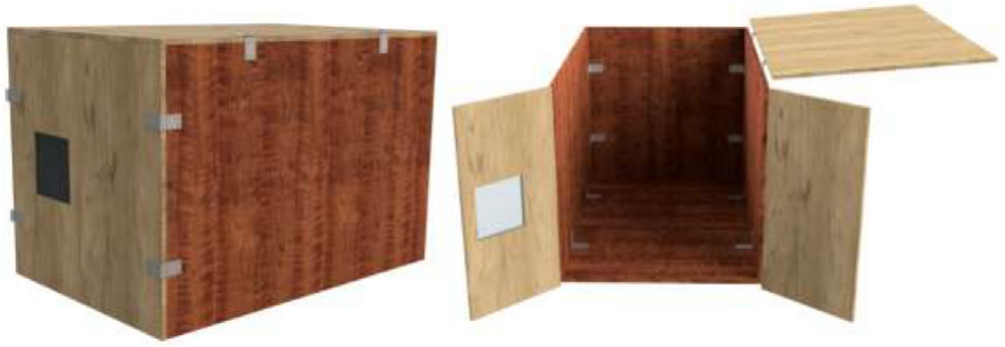
Alternative build.

An alternative to interconnected IKEA closets is to make the chamber entirely of 16 mm thick wood panels. CAD and mechanical drawings are included. Mount plates using angle brackets and wood screws, as seen in Fig. 15, seal all edges using HTS and insulate.

### Incorporating heating and cooling

**Fig. 16:**
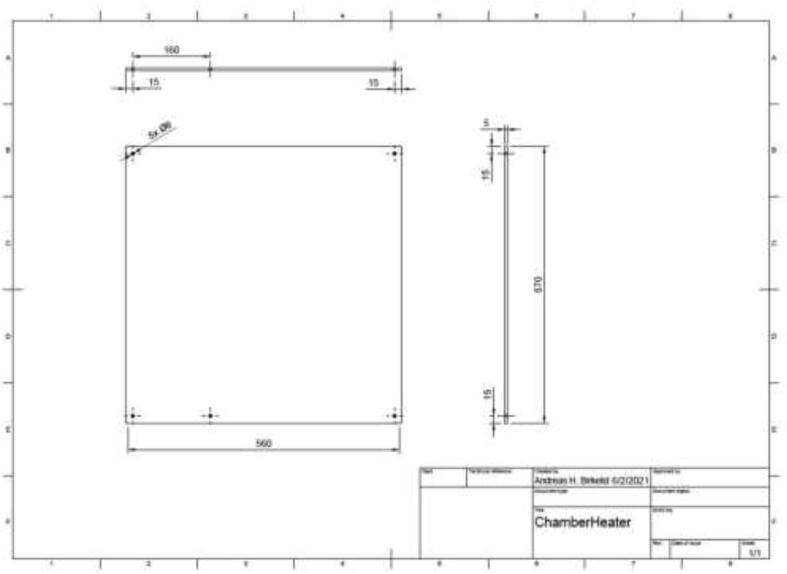
Chamber heater aluminum plate.

The chamber is heated with a 2.5 kW, 220 V AC, 500 mm × 500 mm silicone heater, mounted to an aluminum plate to dissipate the heat. The heater’s wattage will determine how fast the chamber can reach the desired temperature and limit the maximum temperature. A heater that provides above 1300 W should be sufficient, preferably with an included PID controller, simplifying the build; however, the controller may also be implemented manually. A heat-resistant fan for chamber circulation may also be added to decrease the time to heat the chamber and better heat distribution. However, we chose not to add a fan as preliminary temperature measurements suggested a minimal fluxation of temperature by a couple of degrees. Start by cutting and drilling an aluminum plate to the dimensions seen in Fig. 16.

**Fig. 17:**
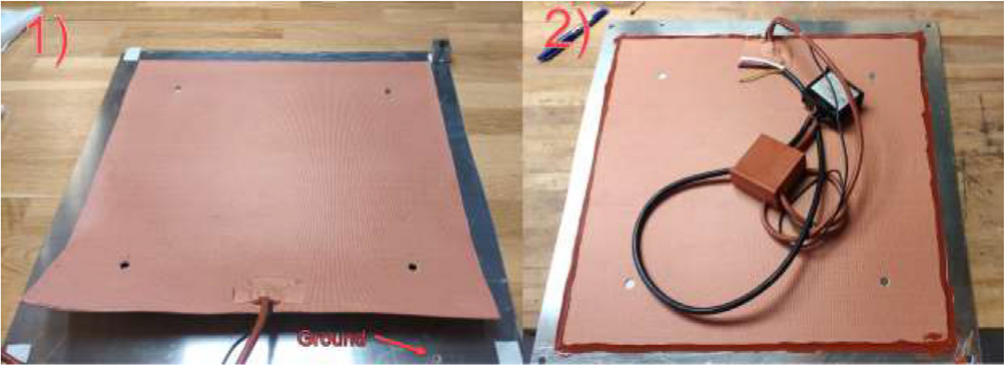
Silicone heater applying steps. 1) Glue silicone heater and add grounding cable. 2) Add HTS around edges.

Clean the plate with isopropyl alcohol and glue the silicone heater to the aluminum plate (see Fig. 17). Start with one side and gradually glue the heater while applying pressure, forcing out air bubbles. Try to fit the mat as close to the middle as possible. To ensure that the mat does not loosen in the edges, add HTS and wait until fully hardened before proceeding. Using an M5x10mm bolt, a nut, and a cable crimp, mount high-temperature silicone wire (HRW) to the hole marked in Fig. 17 to ground the plate.

**Fig. 18:**
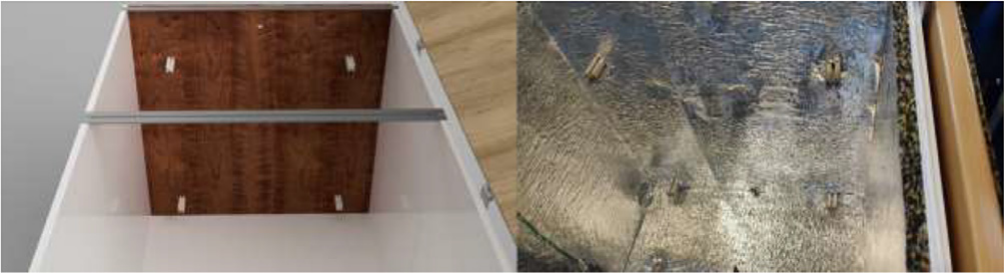
Aluminum profile mounting.

Proceed with cutting 4 × 2020 aluminum extrusions to equal lengths of 60 mm and tap in both ends with M5. Next, cut holes through the isolation, matching the outer 6 mm holes created in the backplate (Fig. 6), and mount the profiles to the backplate using M5x40 mm (Fig. 18).

**Fig. 19:**
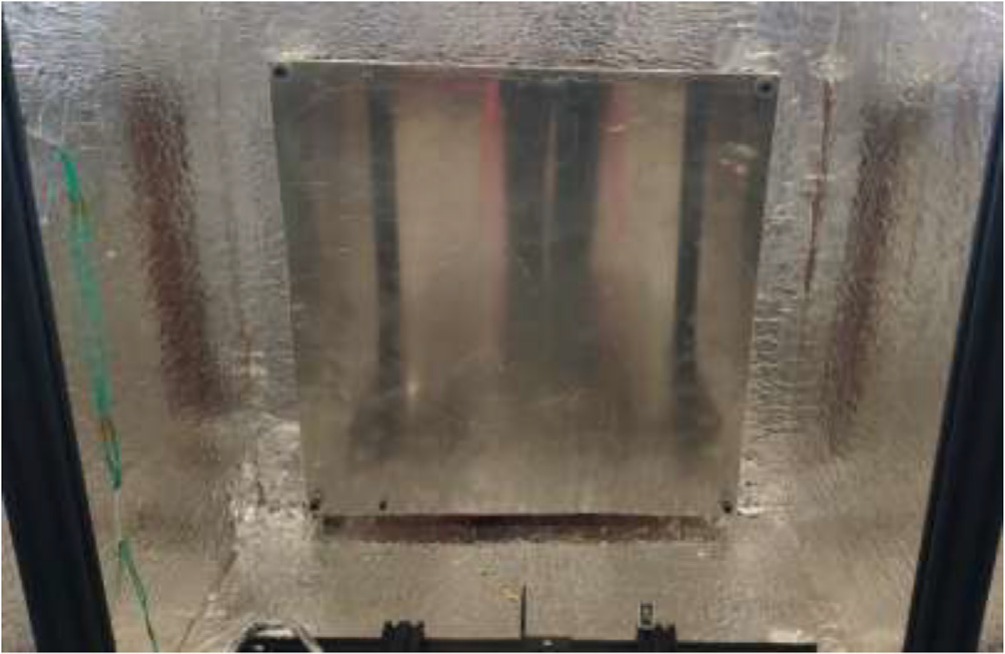
Chamber heater mounting.

Measure the size of the cables included with the silicone heater and drill a suitable hole in the bottom middle of the backplate. If the silicone heater has a PID controller included, note the wiring, and disconnect the controller. Next, pull cables for the silicone heater and grounding cable through the backside and mount the chamber heater to the aluminum profiles using M5x20 mm (Fig. 19).

**Fig. 20:**
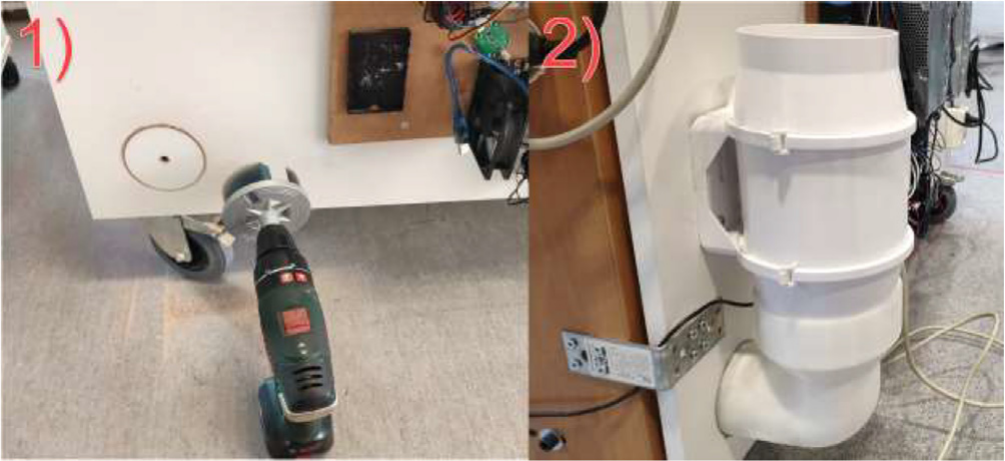
Exhaust fan mounting steps. 1) Drill a hole for the exhaust. 2) Install fan and printed bracket.

To be able to decrease the chamber temperature, an exhaust fan is installed. Start by printing ExhaustFanOutlet.stl in HF. Next, drill a hole with 93 mm diameter in the bottom back of the chamber, as in Fig. 20. Make sure not to interfere with the bottom plate of the chamber. Insert the printed part and screw the exhaust fan to the wall—finally, mount 125 mm ventilation hose to exhaust fan using a hose clamp.

**Fig. 21:**
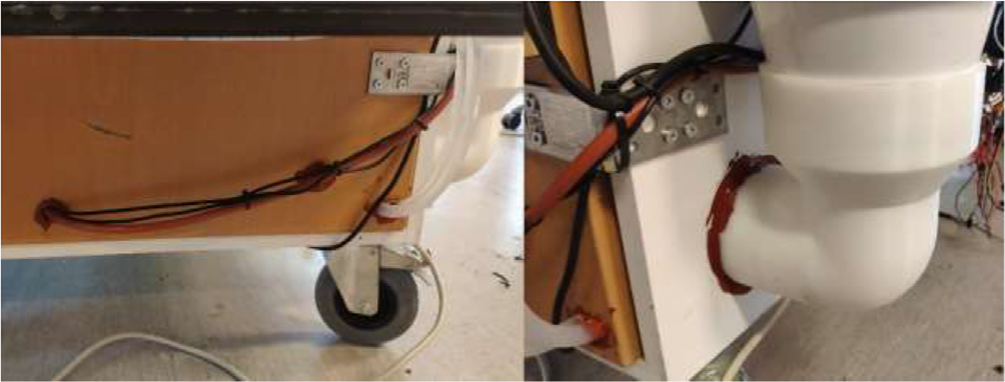
HTS sealing of chamber heated cables (left) and exhaust fan (right).

Proceed to seal the exhaust fan and chamber heater cable output using HTS, as seen in Fig. 21.

**Fig. 22:**
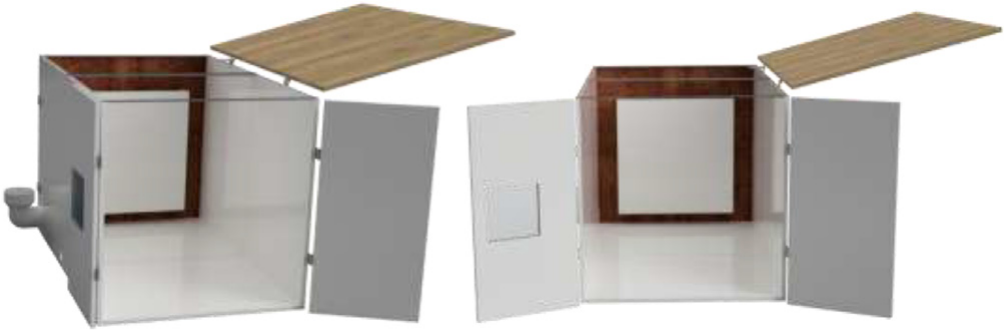
Temperature-controlled printing environment.

The chamber should now look according to Fig. 22.

### Reservoir and radiator holder

**Fig. 23:**
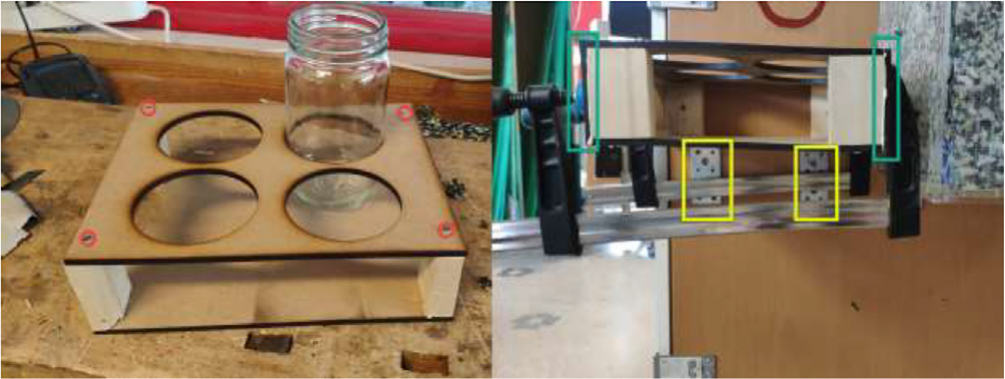
Reservoir holder. Wood screws (red), angle brackets (yellow), and reservoir holder sides (green). (For interpretation of the references to color in this figure legend, the reader is referred to the web version of this article.)

The stepper motors and hotend are cooled by liquid-cooling loops enabling low operating temperatures inside the heated environment. This subchapter will go through the instructions for creating a holder for the reservoirs and radiators. Use a laser cutter or hand tools to cut 6 mm plywood into the dimensions in the repository DXF and STEP files for reservoir and radiator holders. Assemble reservoir holder and mount to wall (Fig. 23) using four 70 mm long wood blocks, wood screws, and wood glue. Screw into the top left side of the backplate, and add additional support using angle brackets in the bottom. Glue sides to the holder to increase strength.

**Fig. 24:**
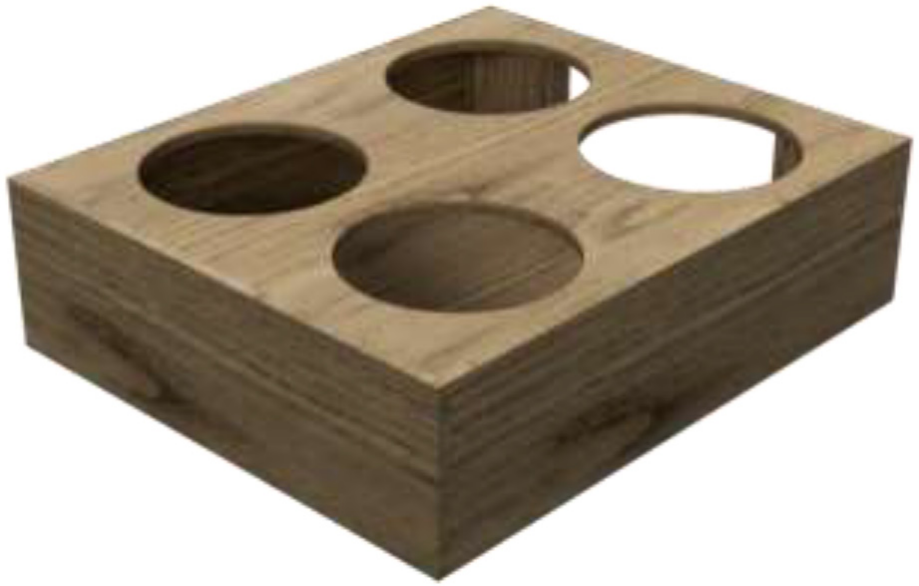
Radiatorholder.

Proceed to glue the front plate to the reservoir holder (Fig. 24). According to the repository, the new reservoir covers are made by either 3D-print, laser-cut, or cutting existing covers according to DXF, STEP, and STL files. Create 1x SmallLid, 1x MediumLid and 2x LargeLid. Finally, insert Ø100 reservoirs and Ø86 lids into the stand, each with a new cover.

**Fig. 25:**
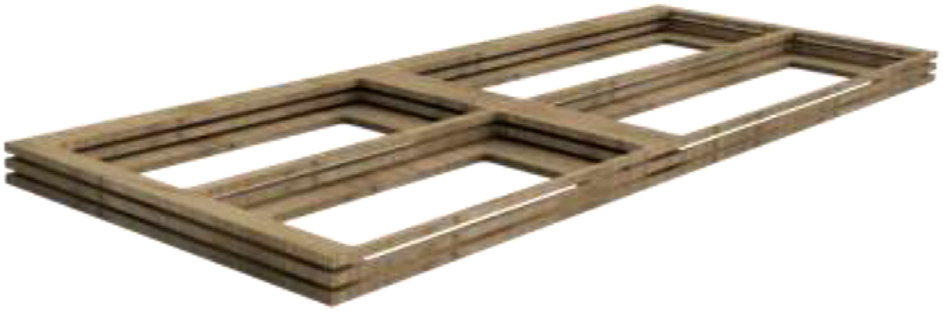
2x RadTop and 1x RadBottom assembly.

Proceed to build the radiator holder. Glue RadBottom and two pieces of RadTop using wood glue in the order noted in Fig. 25.

**Fig. 26:**
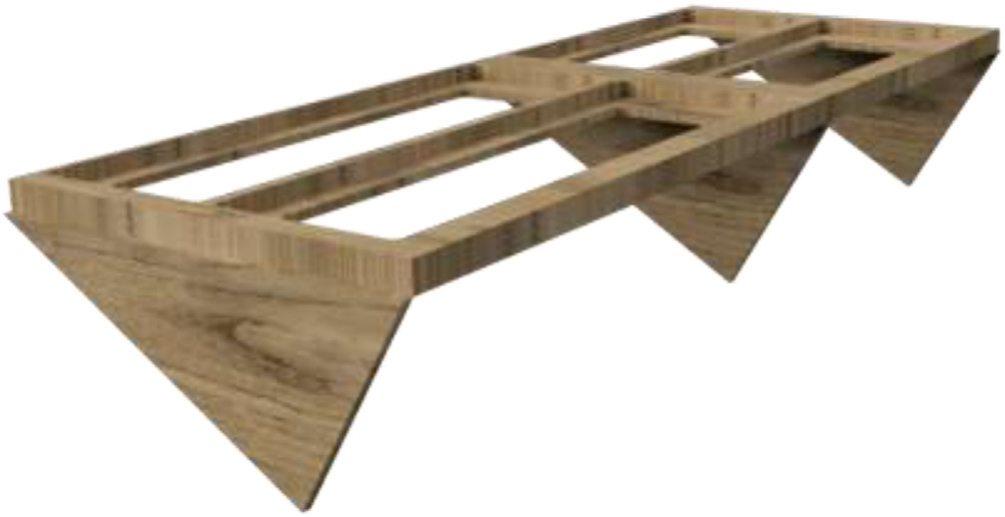
Radiator holder support.

Next, glue three pieces for the RadSupport (Fig. 26). Wait until the glue is thoroughly dried before proceeding to the next step.

**Fig. 27:**
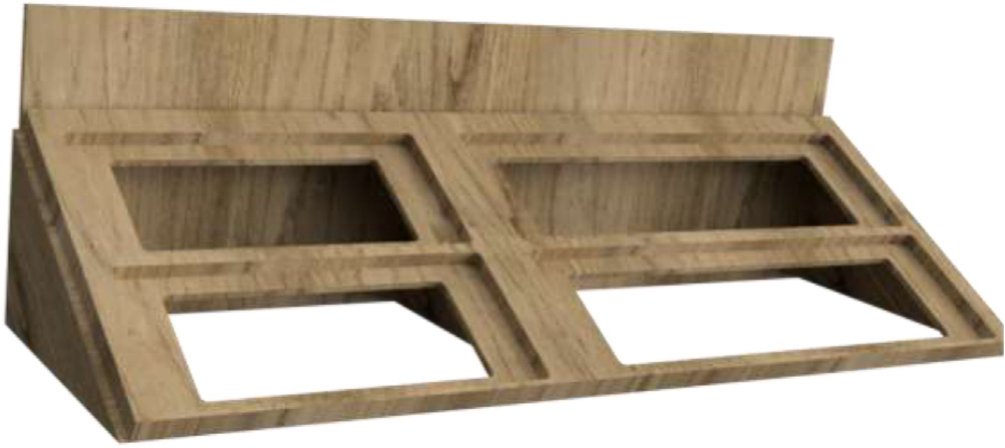
RadBack installed.

Install RadBack as shown in Fig. 27 to complete the assembly.

**Fig. 28:**
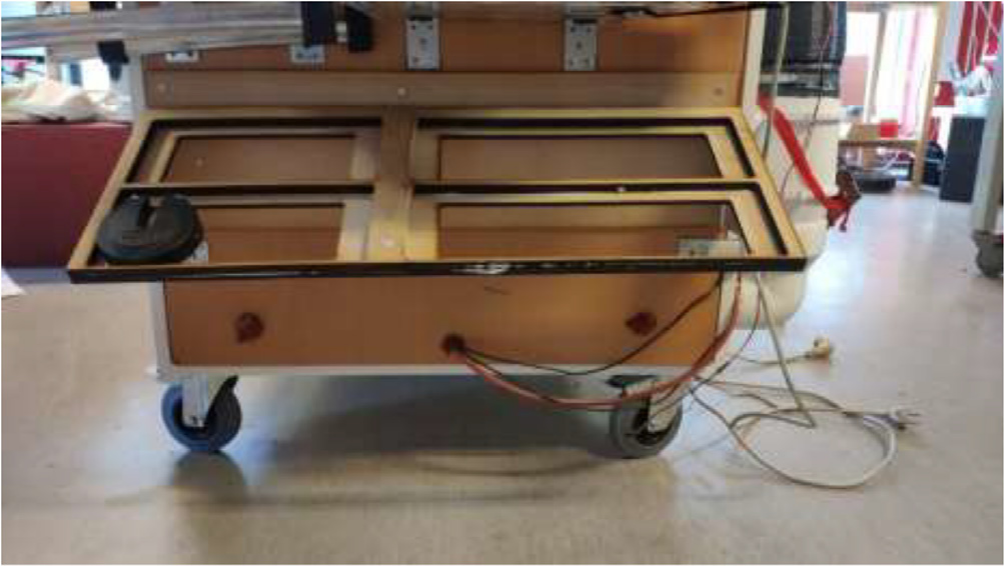
Radiatorholder mounted on backplate.

When thoroughly dried, the radiator holder is then mounted to the backplate using several wood screws, as seen in Fig. 28.

### Disassembly of 3D printer

**Fig. 29:**
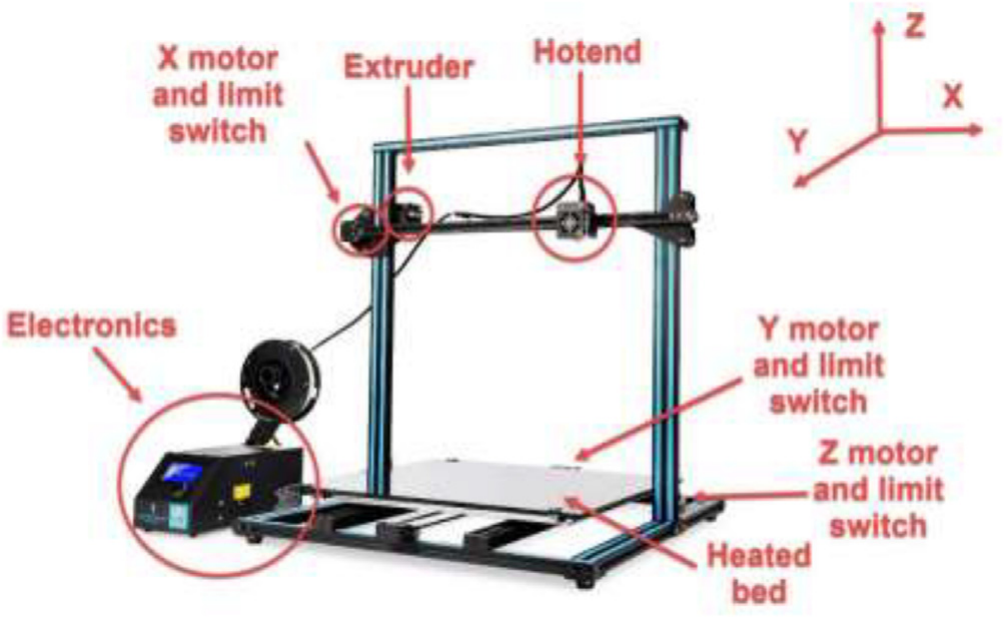
CR10 Plus printer, its components, and the coordinate system[17]

This article used a CR10 Plus printer as the base for the high-performance printer due to the large build volume and easily accessible components. Other printers could also be selected; however, one should choose a printer with minimal printed parts since they have to be replaced by metal parts. The components, the printer, and its coordinate system are presented in Fig. 29.

**Fig. 30:**
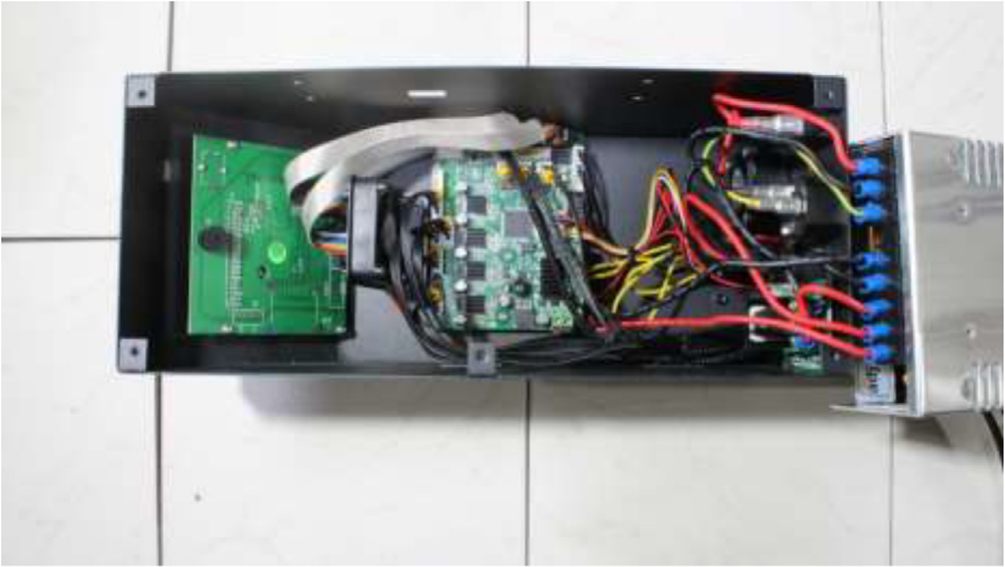
Electronics box [18]

The 3D printer will be fully disassembled, leaving only the frame. Please take note during the disassembly as it will be assembled in the reverse order. Start by opening the electronics enclosure as seen in Fig. 30, disconnecting all wiring, and removing all components from the enclosure. The LCD and the casing will not be used.

**Fig. 31:**
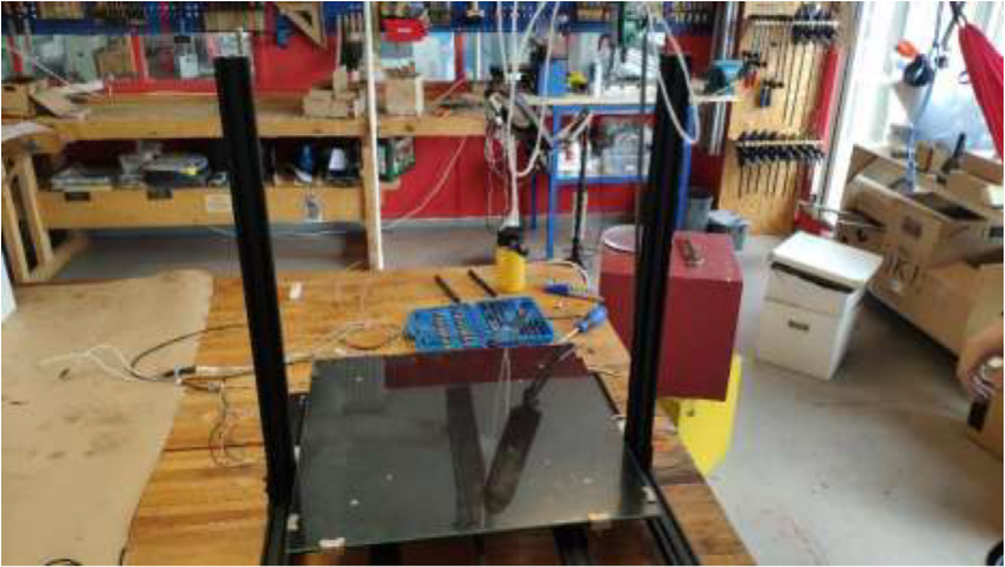
Removal of the top bracket.

Remove limit switches for the X, Y, and Z-axis and all plastic covers on the printer. Next, remove the top bracket and Z rod holders of the frame (Fig. 31).

**Fig. 32:**
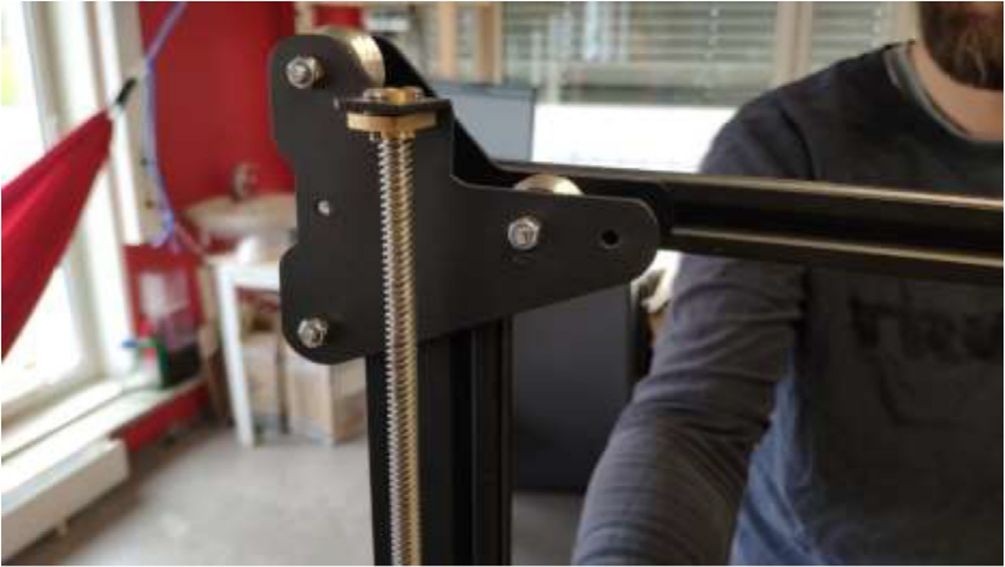
Removal of the X-axis.

Next, remove the X-axis by turning the Z-axis leadscrews to the top (Fig. 32).

**Fig. 33:**
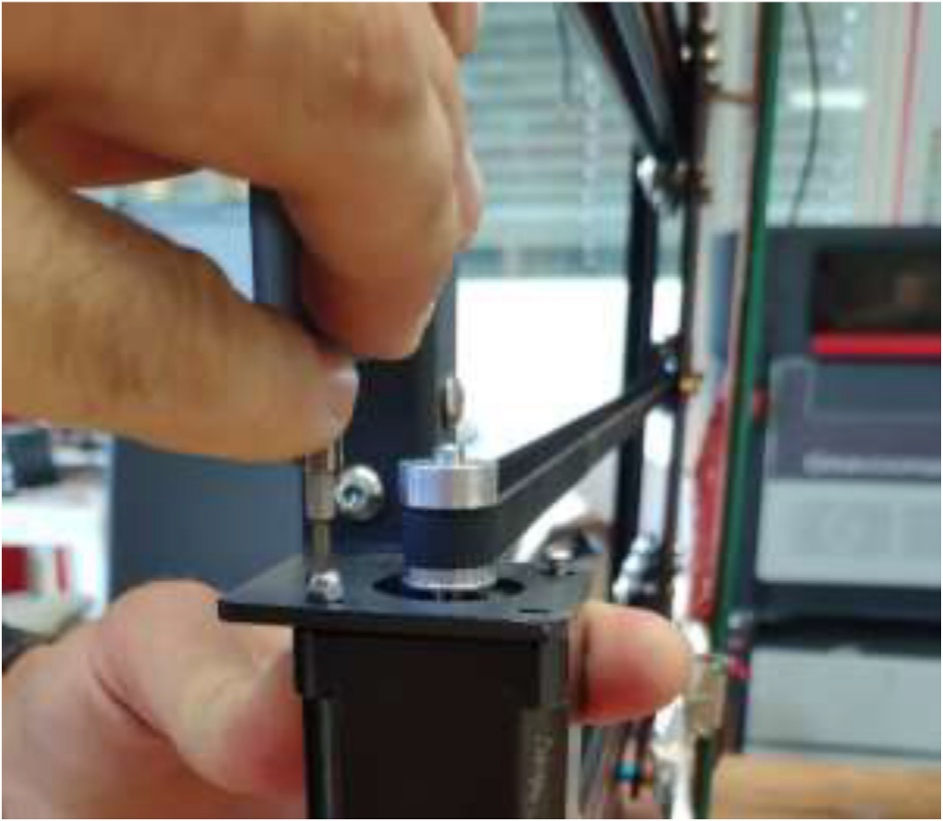
Removal of Y motor.

Remove Z steppers by unscrewing them from their mount. Next, do the same for the Y motor, as seen in Fig. 33.

**Fig. 34:**
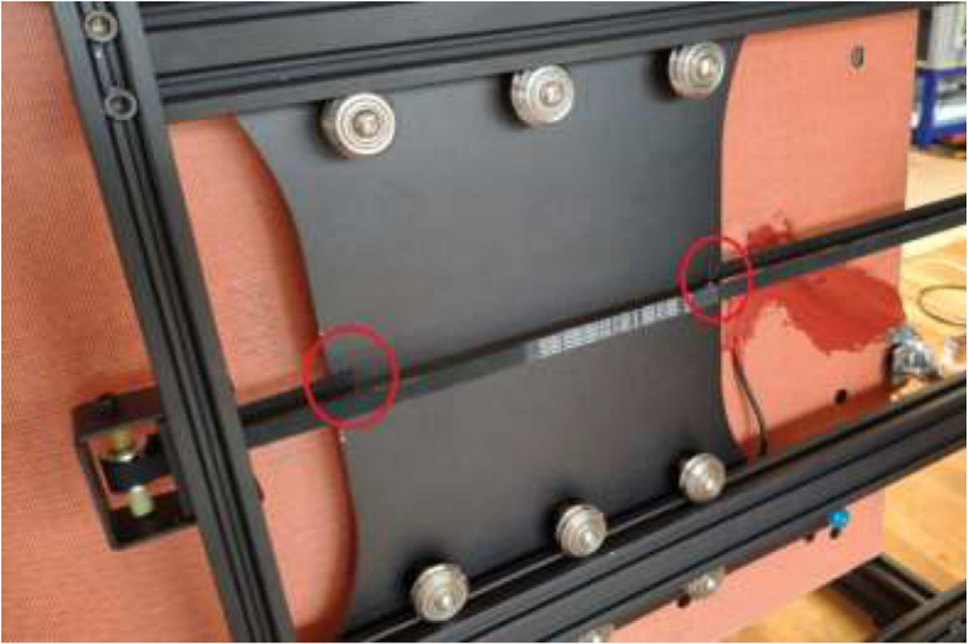
Removing belt from Y carriage.

Disassemble the belt on the Y-axis by pulling it to the side (Fig. 34), removing the Y motor mount, and pulling the Y carriage out of the frame.

Remove the belt tensioner for X-axis and the X carriage before removing the extruder and hotend from the carriage. The hotend and extruder will not be used later in this build.

### Stepper motor modifications

**Fig. 35:**
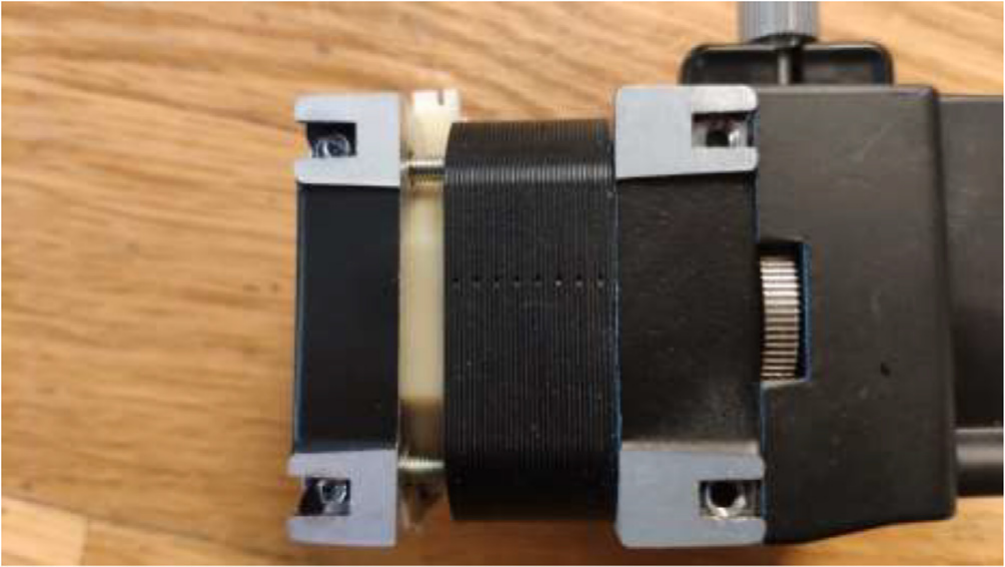
Removing back cover of stepper motor.

Nema17 stepper motors will start to skip steps and demagnetize if overheated. Skipping of steps depends on brand and exposure time, but generally happends at around 80 °C. Cooling blocks and HRW for water cooling are installed next, making the motors compatible with a heated environment. Next, disassemble each stepper motor by removing the four bottom screws and pulling the back cover of the motor (Fig. 35).

**Fig. 36:**
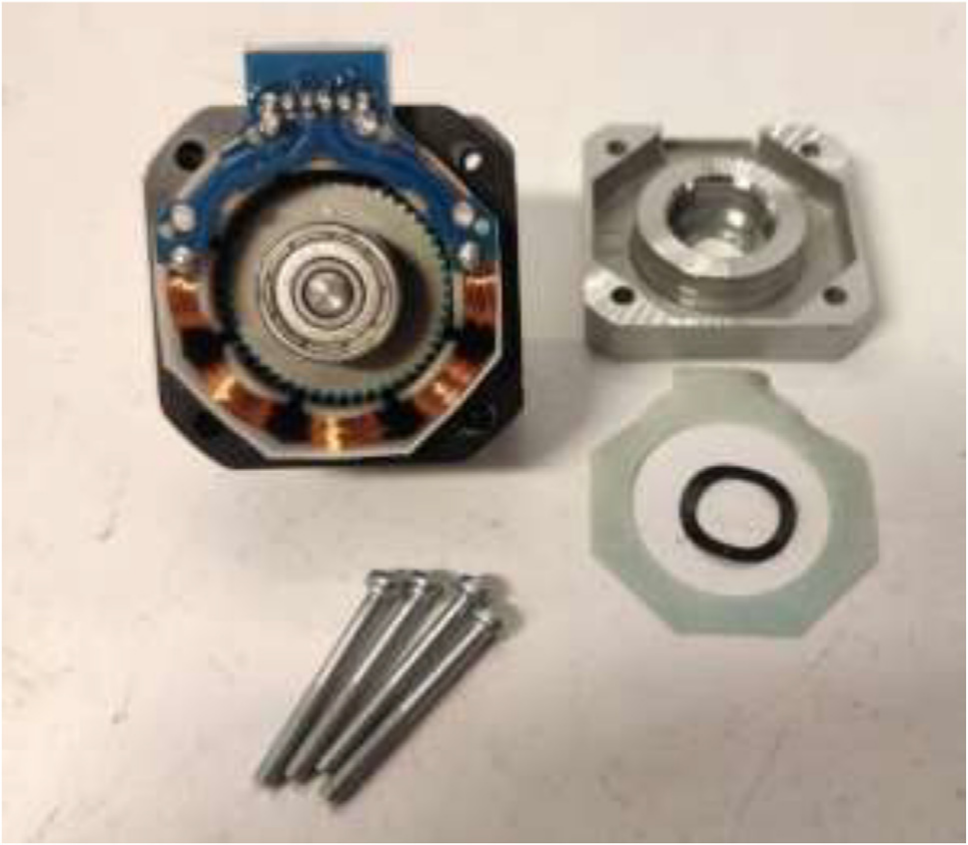
Stepper motor disassembled.

After disassembly, it should look like Fig. 36.

**Fig. 37:**
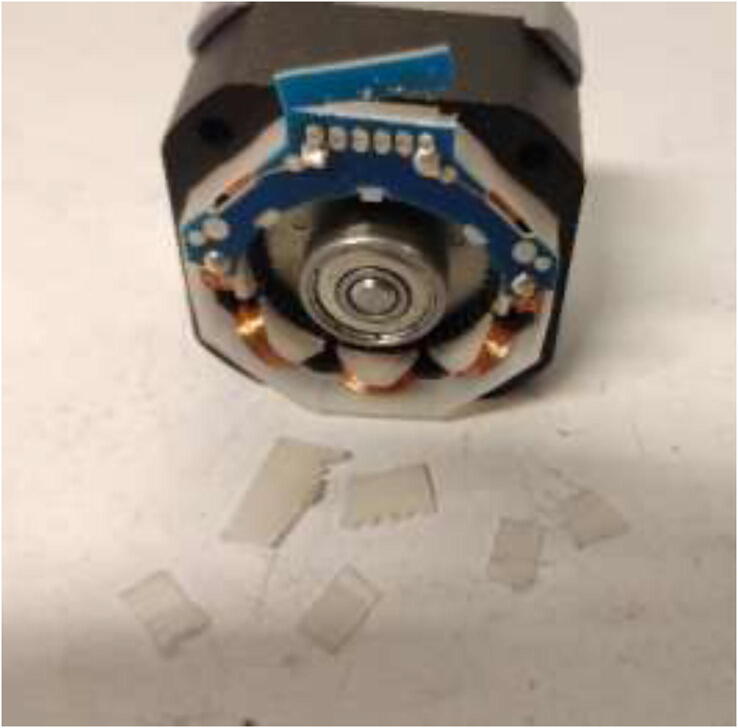
Removal of stepper motor connector.

De-solder the connector and trim the PCB and plastic, as shown in Fig. 37. Take a picture of the wiring and cable colors for later.

**Fig. 38:**
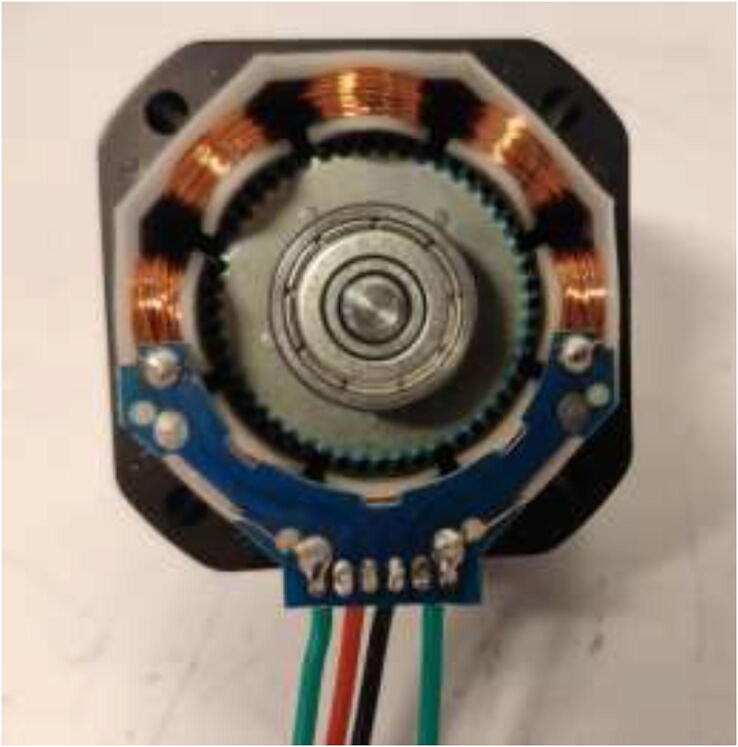
Soldering HRW.

Solder 26AWG HRW to the solder pads as shown in Fig. 38. Ensure that the color-coding is correct for the stepper motor, as this may vary. Solder the ends of the wire to the old connector.

**Fig. 39:**
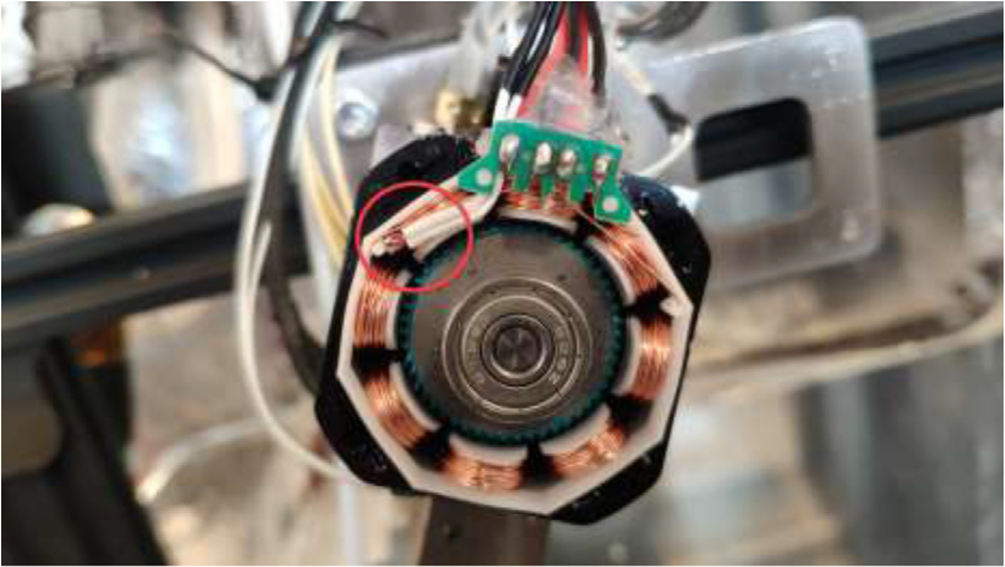
Inserting thermistor for temperature observation.

An NTC 100 K thermistor could optionally be installed under the bottom cover. It is recommended to put the thermistor on top of the stator and hold it in place with glue as seen in Fig. 39. Reassemble the motor using the assembled steps in reverse.

**Fig. 40:**
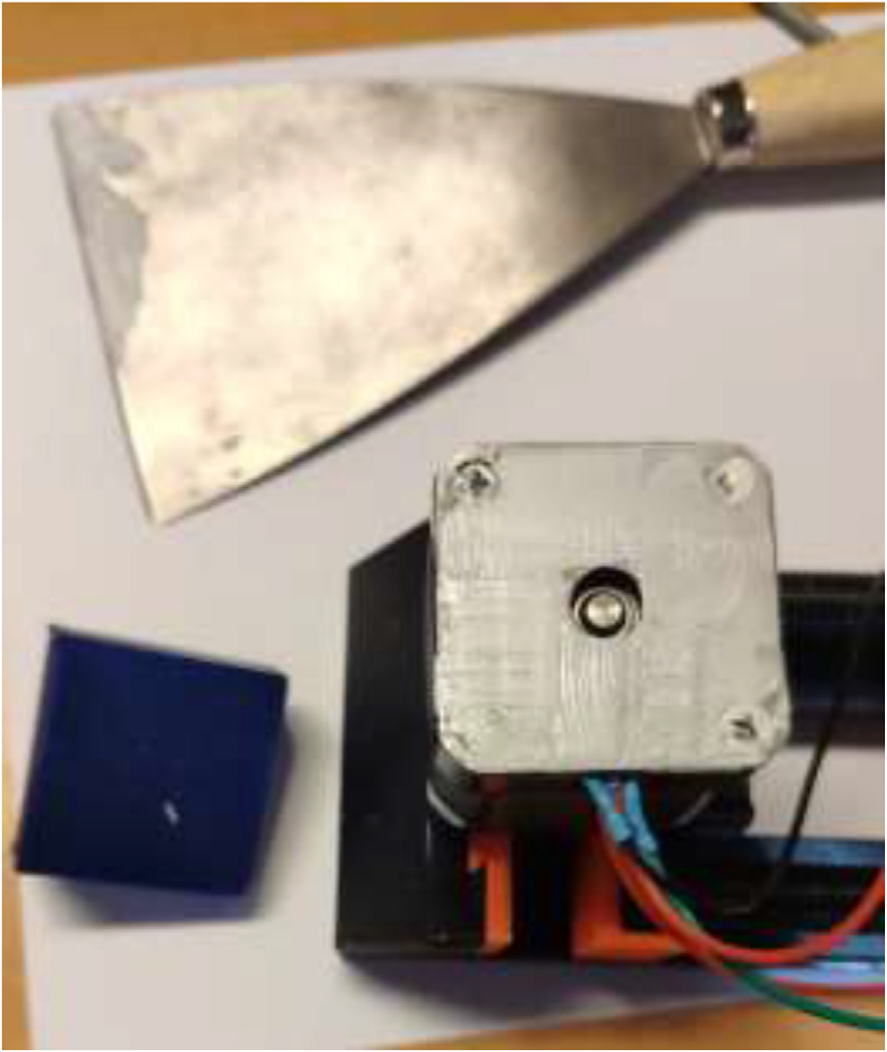
Apply a thin layer of heatsink plaster after grinding and washing.

Grind half a millimeter of the bottom cover, wash with isopropyl alcohol and apply a thin layer of heatsink plaster, as seen in Fig. 40.

**Fig. 41:**
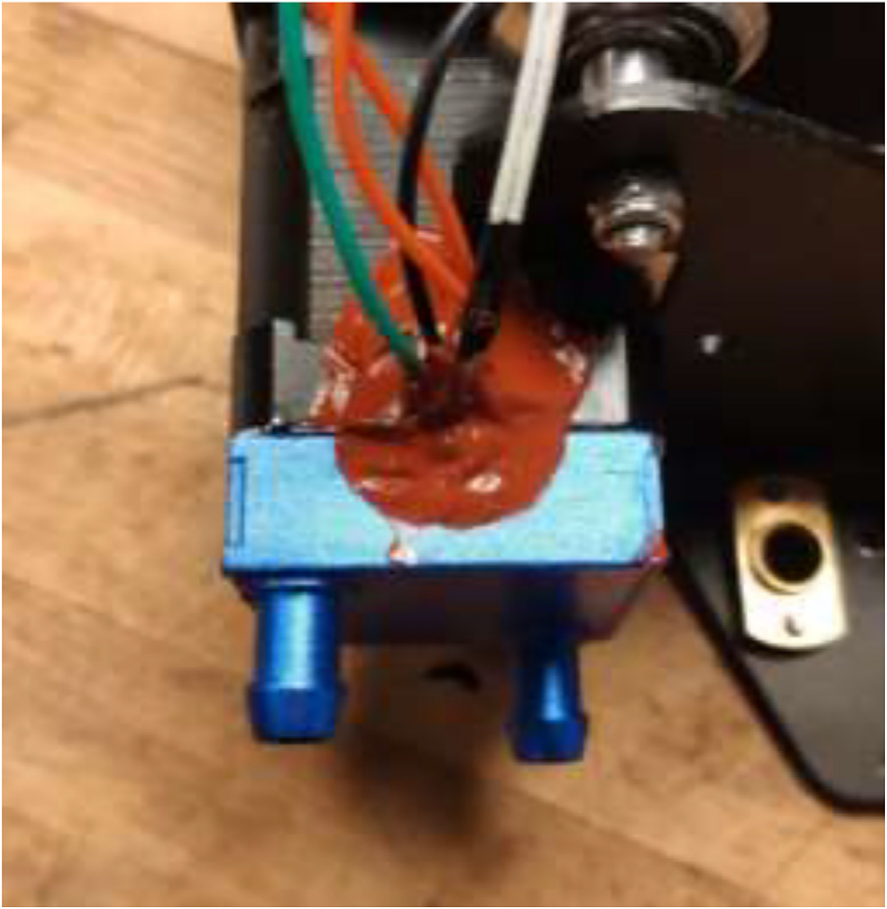
Mount cooling block and add HTS.

Proceed to mount a 40x40mm CPU cooling block with the preferred orientation to the stepper motors. Add HTS to seal the motor and provide strain relief to prevent the wires from being pulled out, as seen in Fig. 41. Wait until the heatsink plaster and HTS are fully hardened.

### Liquid-cooled extruder

**Fig. 42:**
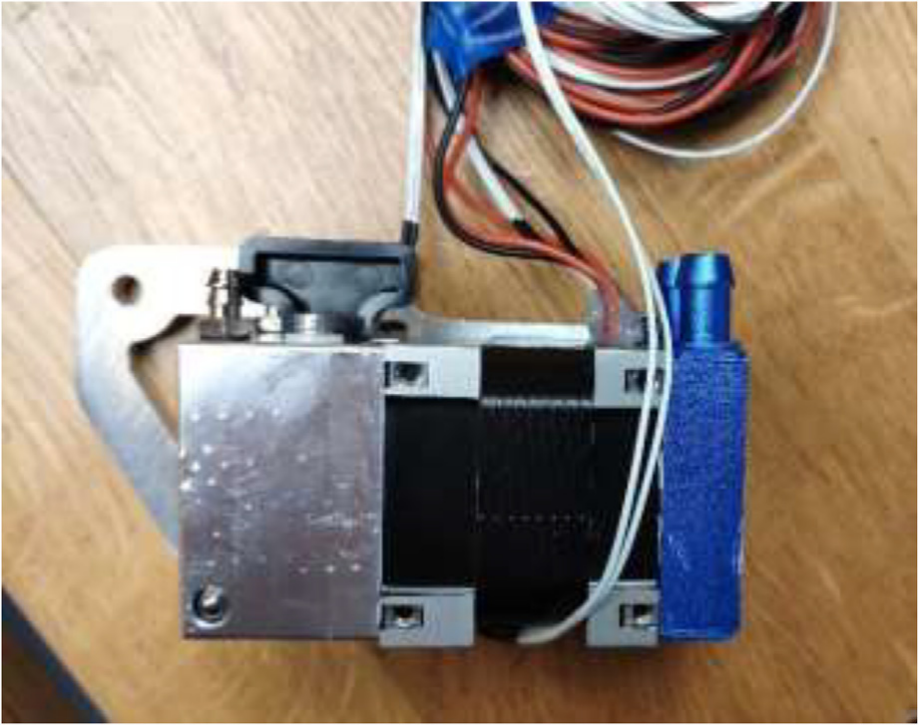
TriangleLab Matrix Liquid-Cooled extruder.

Having a direct drive extruder instead of a remote extruder will shorten the filament path and give greater filament control and print quality. Therefore, the TriangleLab Matrix liquid-cooled extruder is chosen (see Fig. 42), but other liquid-cooled extruders may also work.

**Fig. 43:**
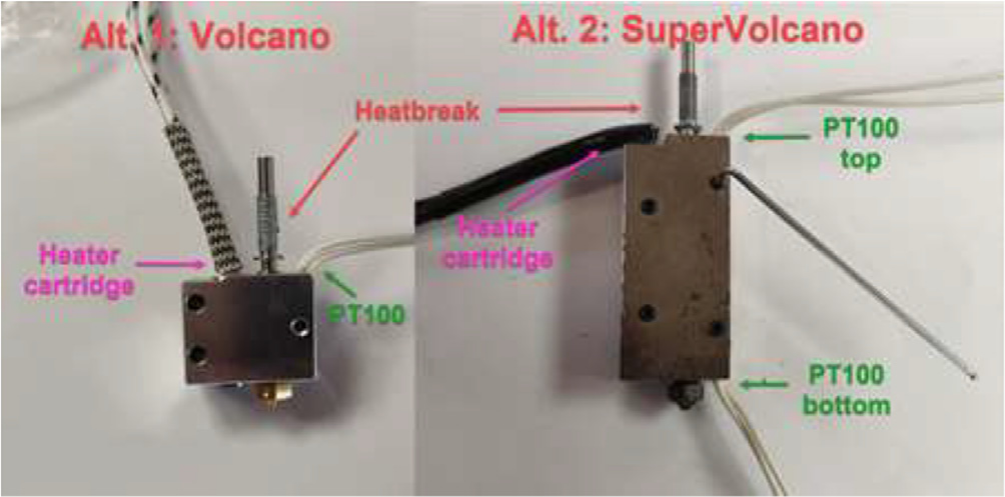
Volcano (left) and SuperVolcano (right) hotend assembly.

Start by disassembling the extruder and upgrade the stepper motor, as shown in section 5.3. The build is compatible with both a Volcano and a SuperVolcano hotend. It is noted that a SuperVolcano will increase the maximum possible volumetric flow but decrease the build volume. Next, add thermal compound to the cold side of the heat break, screw nozzle, and heat break into hotend and add heater cartridge and PT100 sensor as seen in Fig. 43. Optionally, one could add a PT100 sensor in the top end of the SuperVolcano hotend as there is room for an extra sensor.

**Fig. 44:**
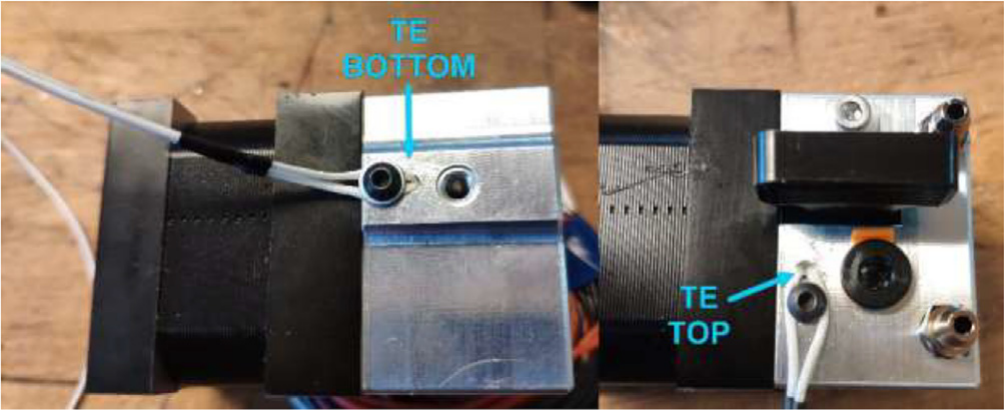
Thermistors installed in the cooling block.

Optionally, one can drill holes in the cooling block to obtain temperature measurements. First, drill a hole and insert an NTC 100 K thermistor. Then, right beside the hole, drill another hole and thread with M3. Using an M3 bolt with a suitable length and a washer, hold the thermistor in place, as seen in Fig. 44. Make sure that the holes do not interfere with the internal geometry of the cooling block.

**Fig. 45:**
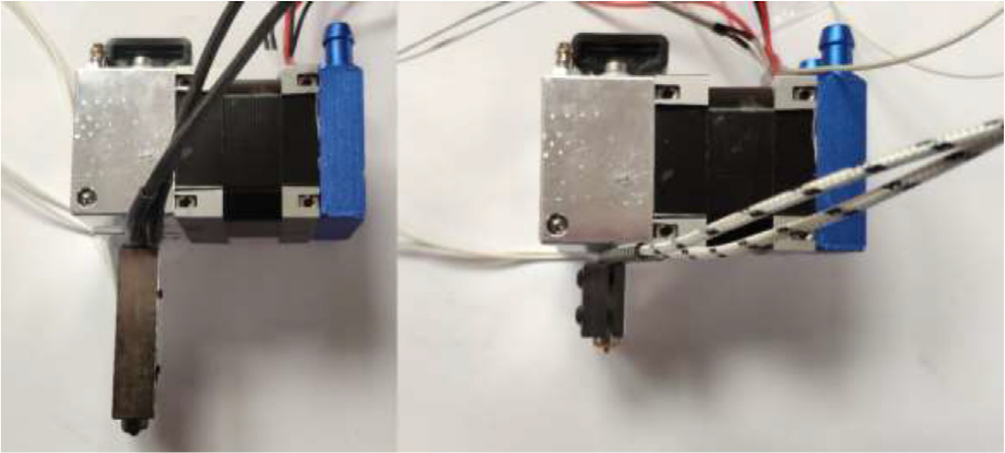
SuperVolcano (left) and Volcano hotend (right) extruder assembly.

Screw the desired hotend into the extruder, Fig. 45, and ensure that it is tightly screwed to avoid leakage. Extend all cables to at least 2 m using HRW. Make sure that the heater cartridge is soldered to a thick (at least 14AWG) cable.

### Upgrading movement system

**Fig. 46:**
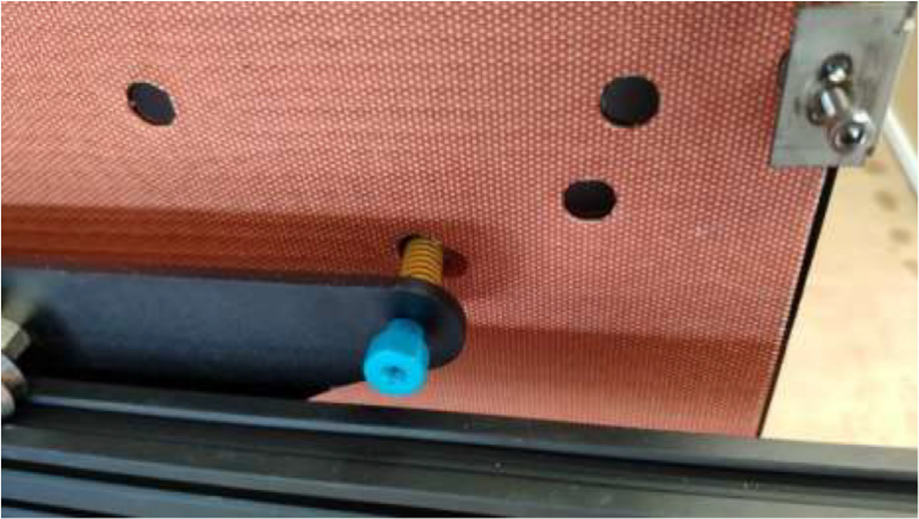
Underside of heated bed with new bed leveling nut installed.

The movement system of a CR-10 Plus printer consists of V-rollers, belts, and leadscrews. V-rollers made of Polyoxymethylene (POM) may start to soften after being exposed to higher temperatures. Also, the original belts will stretch in a heated chamber. Therefore, both the rollers and the belts will be upgraded in this section. Start by removing the heated bed from the Y carriage using the provided plastic bed leveling nuts, replace these with all-metal nuts when assembling the printer later on, as in Fig. 46.

**Fig. 47:**
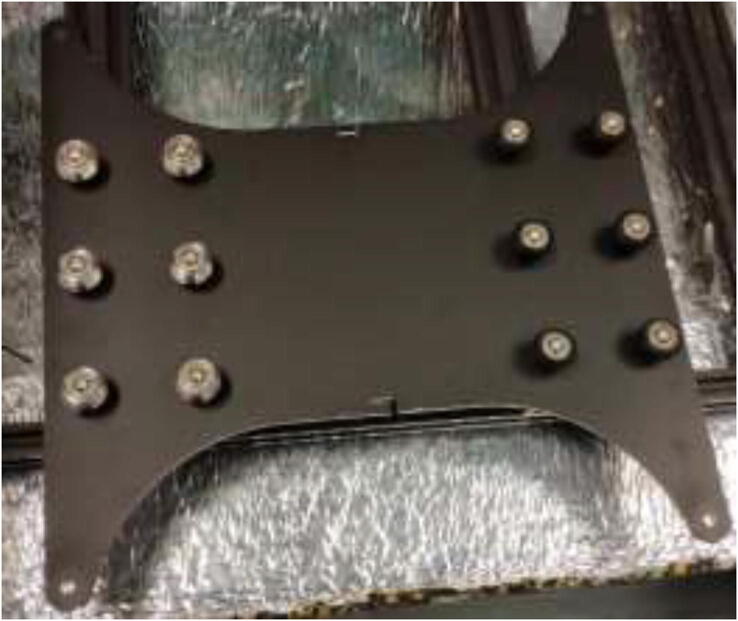
V-rollers in POM (right) should be replaced with rollers in stainless-steel(left).

All POM rollers on the Y carriage are replaced by stainless steel parts, as seen in Fig. 47. Reinstall the carriage and the Y motor mount. Measure the required belt length from the old belt and cut a piece of high-temperature 9 mm wide belt as a replacement. Install the old belt clips on the new belt and install them reversely as it was uninstalled. Next, install the upgraded Y-motor, but do not install the heated bed yet.

**Fig. 48:**
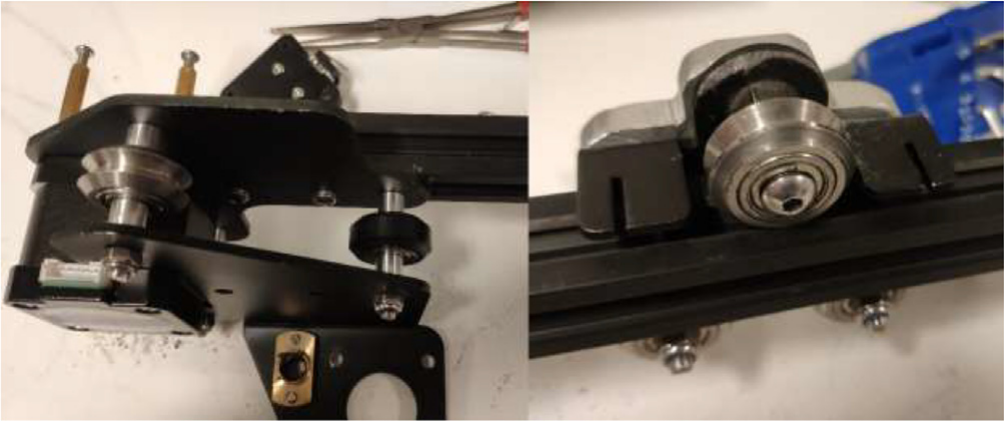
X-axis V-rollers.

Replace all bearings on the X-axis as shown in Fig. 48.

**Fig. 49:**
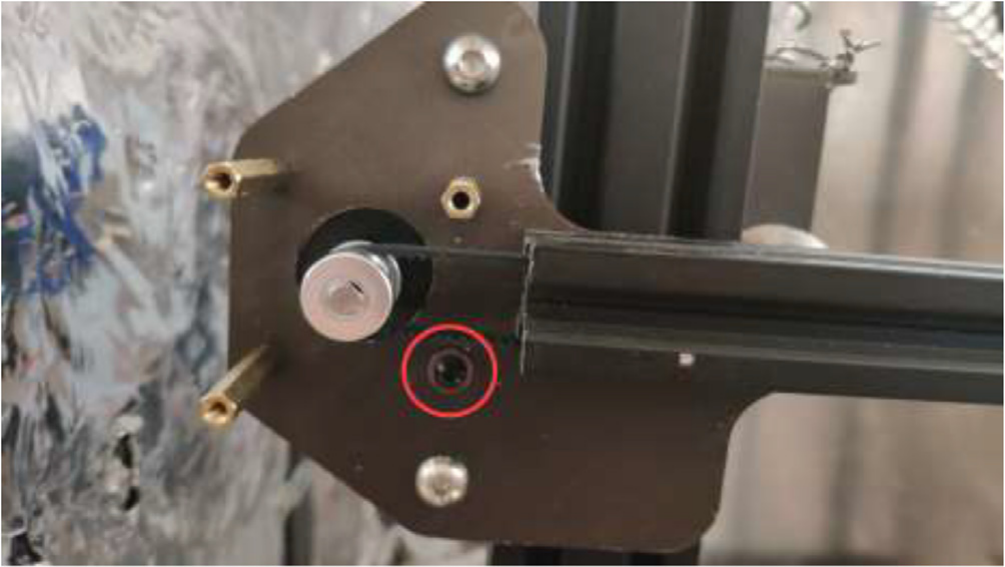
Installing upgraded X-motor and making room for limit switch.

Install the newly upgraded X-motor, replacing one of the long screws with an M3x8 mm and a washer to make room for a new limit switch as in Fig. 49. Upgrade the 6 mm wide belt on this axis with the same procedure as for the Y-axis.

**Fig. 50:**
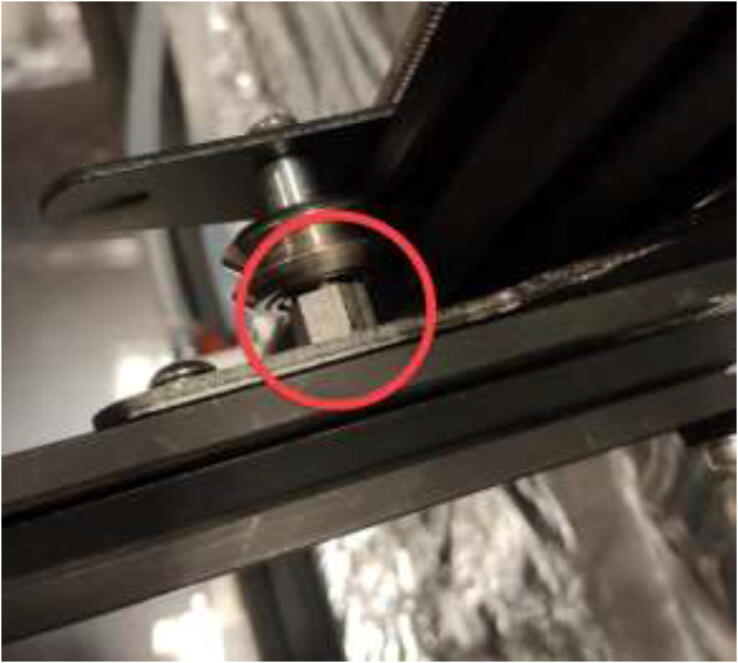
Tension adjustment on axis.

Make sure that the rollers are adequately tensioned and provide a smooth movement. To adjust the tension, rotate each six-headed nuts on the axis, which needs adjustment (Fig. 50).

**Fig. 51:**
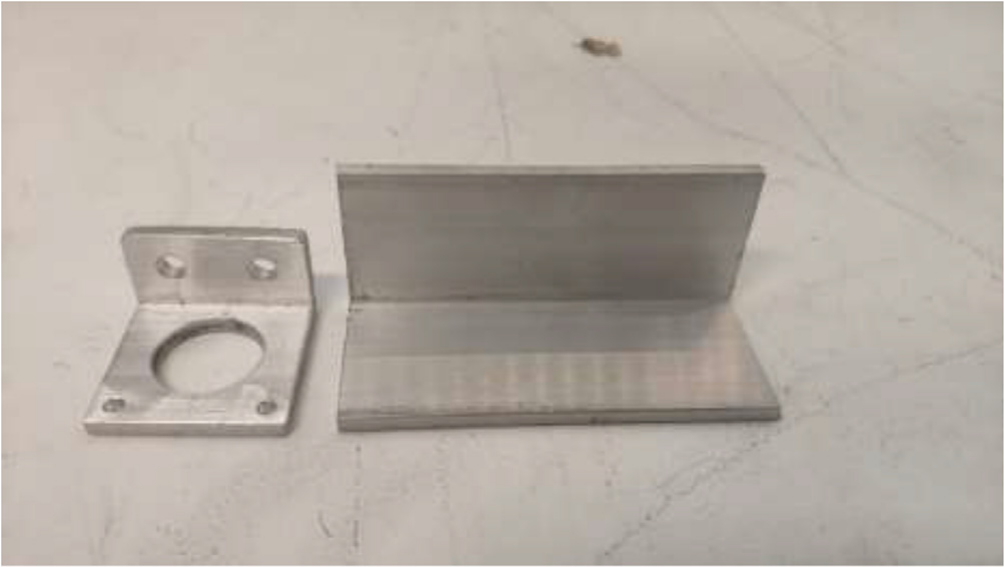
Z stepper motor brackets.

**Fig. 52:**
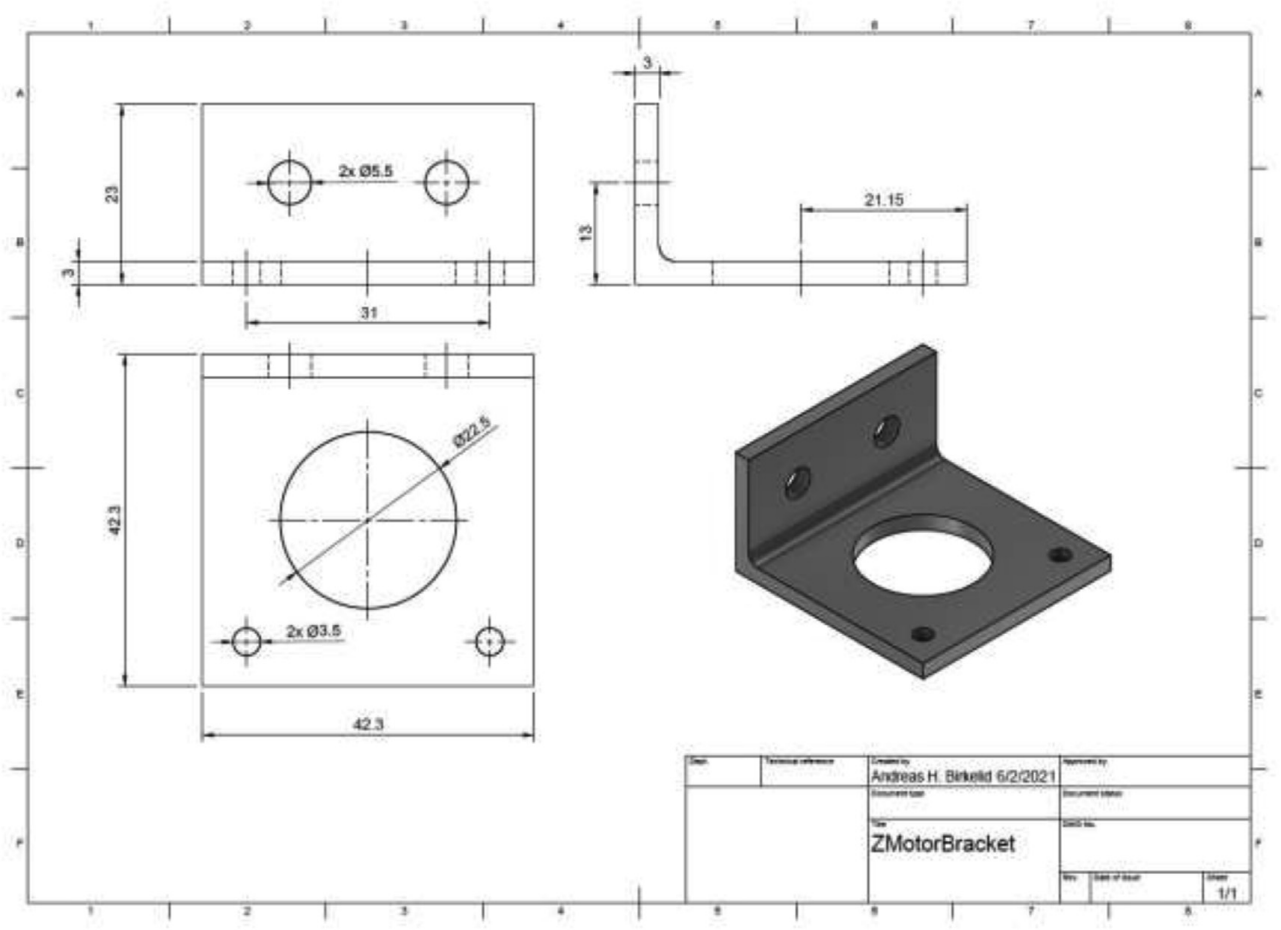
Aluminum bracket drawing.

The original Z-motor holders are made from plastic and should be replaced by aluminum brackets. Use an aluminum extrusion with an L-profile to create the holder, as in Fig. 51 by the dimensions presented in Fig. 52.

**Fig. 53:**
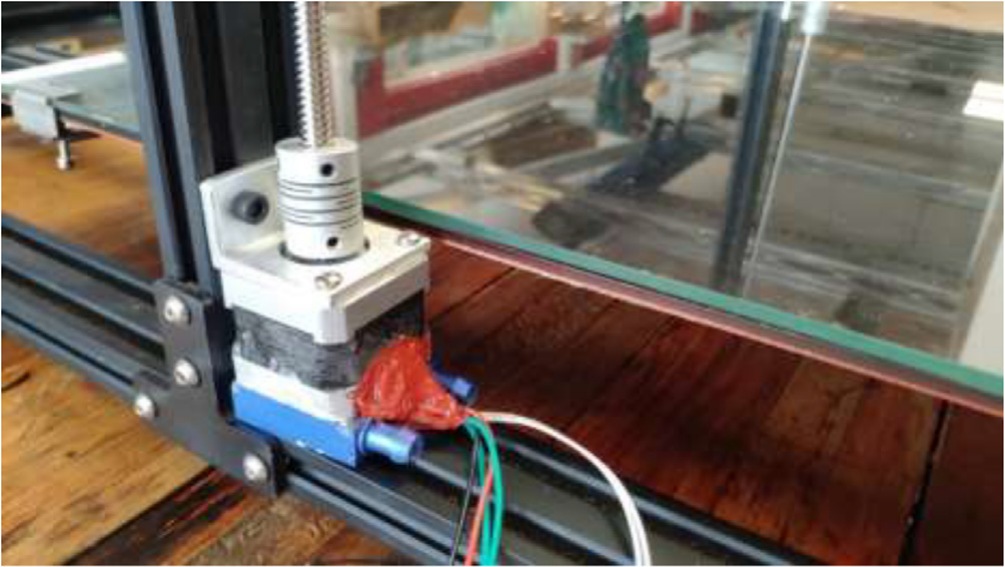
Upgraded Z motors with new Z motor holder.

Mount Z-motors with cooling block to the frame of the printer, as seen in Fig. 53.

**Fig. 54:**
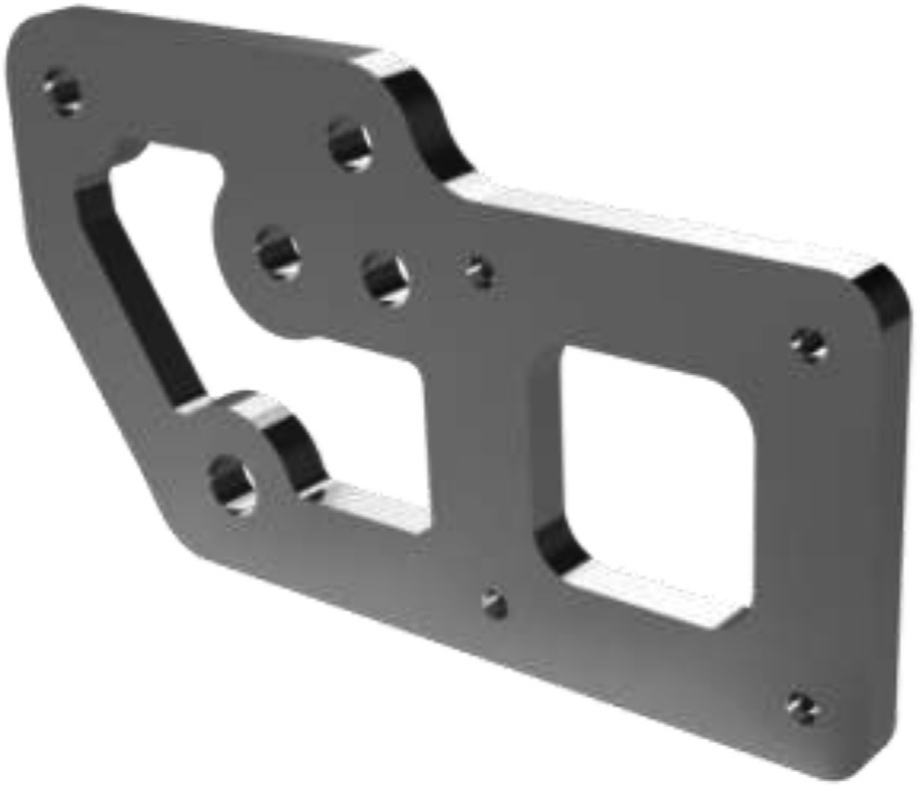
Extruder mount.

A new extruder mount is made to fit the upgraded extruder and files are provided in the repository. Both a complex and simple model is included. One could use CNC tooling to create the complex model (Fig. 54) or 3D print the simple model, then trace and cut using a metal saw. Create the mount using 5 mm aluminum.

**Fig. 55:**
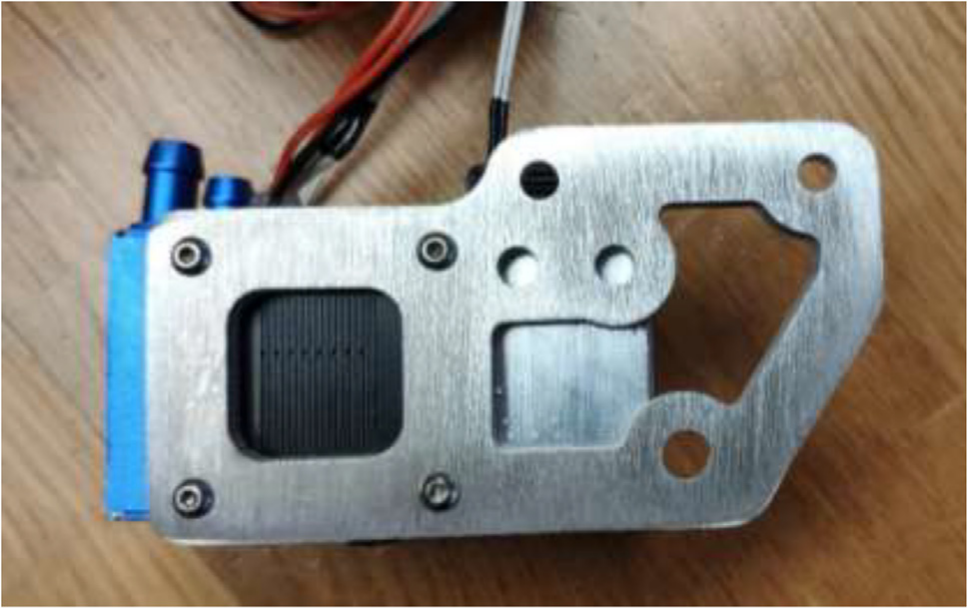
Extruder fixed to the new mount.

Mount extruder to the aluminum bracket using 4x M3x8 mm bolts as seen in Fig. 55.

**Fig. 56:**
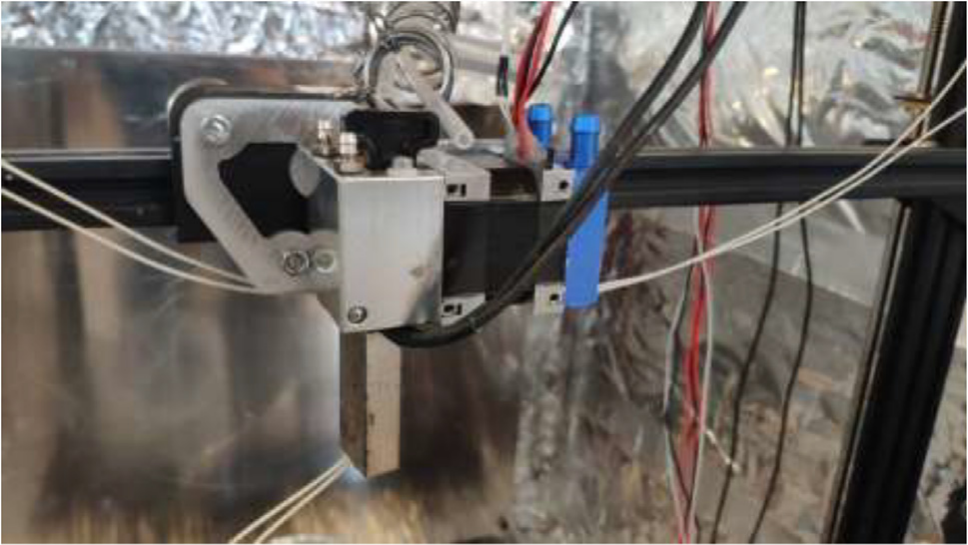
Extruder installed on X-carriage.

Proceed by mounting the adapter to the X-carriage, as seen in Fig. 56.

**Fig. 57:**
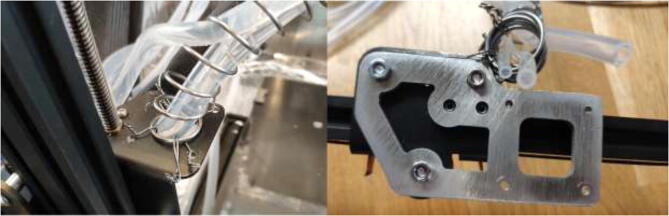
Spring mounting.

A spring with an inner diameter of 25 mm and a length of 1 m will be used to manage cables and silicone tubing from the extruder. Mount the spring to the previously extruder mount and X carriage using steel wire, as seen in Fig. 57.

**Fig. 58:**
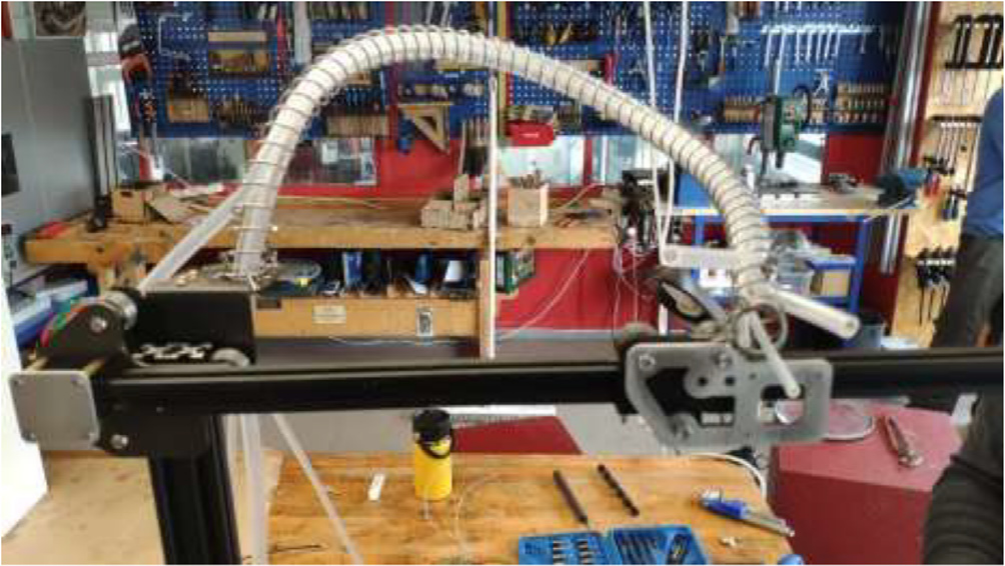
Spring curve for tubing and cable management.

Make sure that the spring will create a smooth curve, as seen in Fig. 58.

**Fig. 59:**
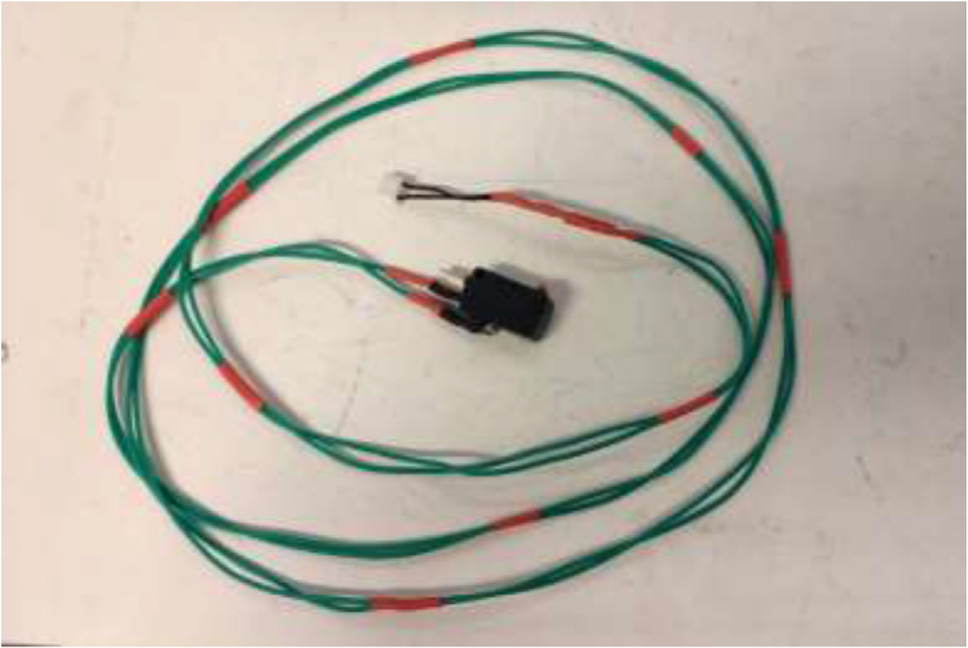
Limit switch wire.

The original limit switches are not rated for high temperatures and need to be upgraded. Start by soldering two meters of HRW to all three limit switches and solder the old limit switch connector for the new switch as in Fig. 59.

**Fig. 60:**
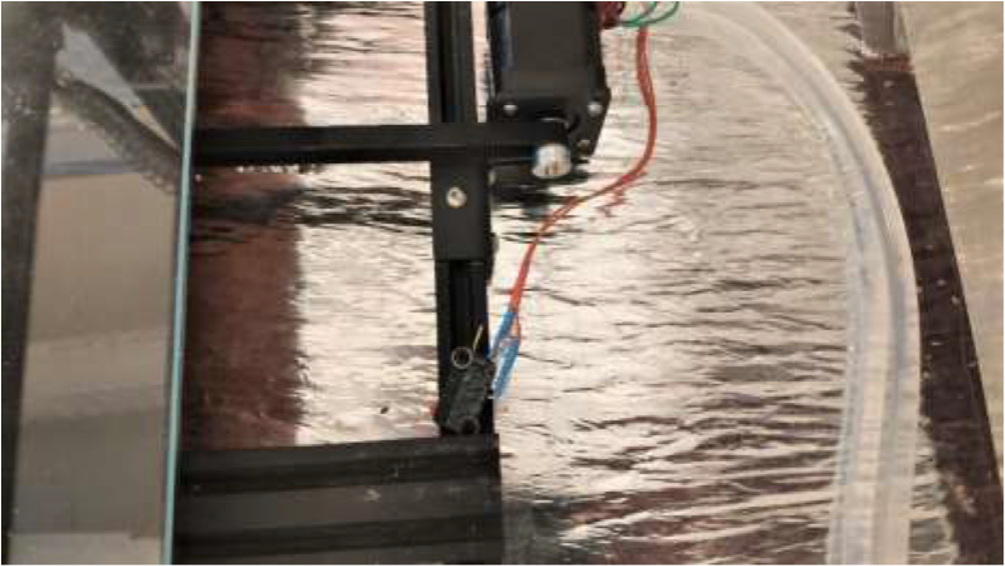
Mounting Y limit switch.

Mount the Y limit switch using 2x M3x16 mm screws, 2x M3 T-Nuts, and 2x washers, as seen in Fig. 60.

**Fig. 61:**
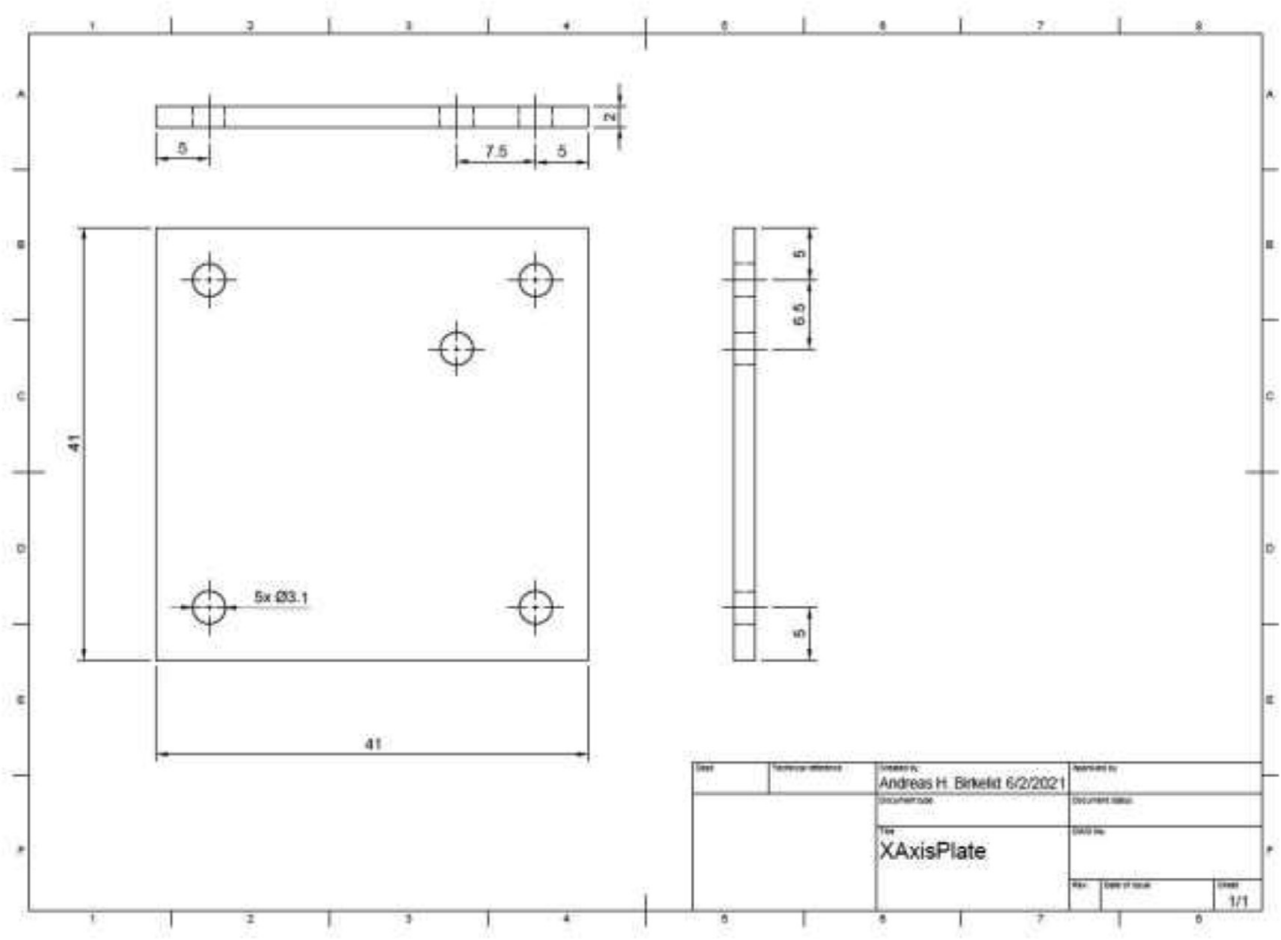
X limit switch holder.

Follow the mechanical drawing in Fig. 61 and create a limit switch holder using aluminum or steel plating for the X-axis.

**Fig. 62:**
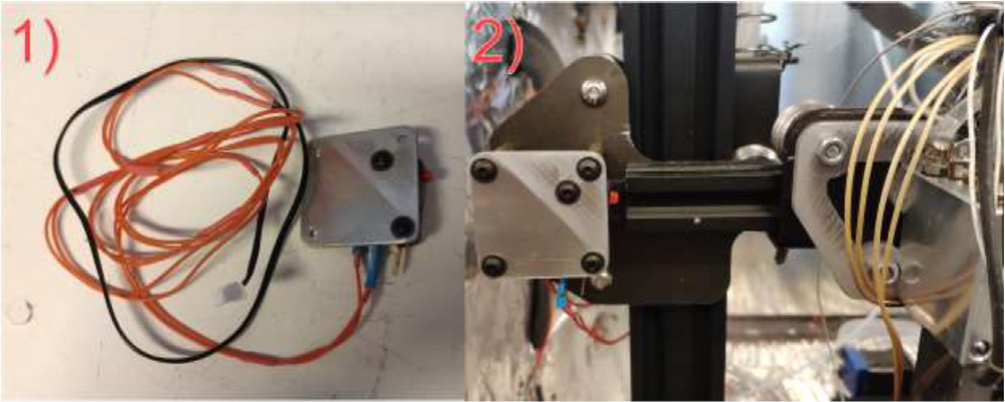
X limit switch.

Use 2x M3x12 mm, 2x washer, and 2x M3 nuts to mount the limit switch to the plate (Fig. 62). Mount the plate and limit switch using 3x M3x12 and 3x M3 nuts as spacers behind the mounting plate.

**Fig. 63:**
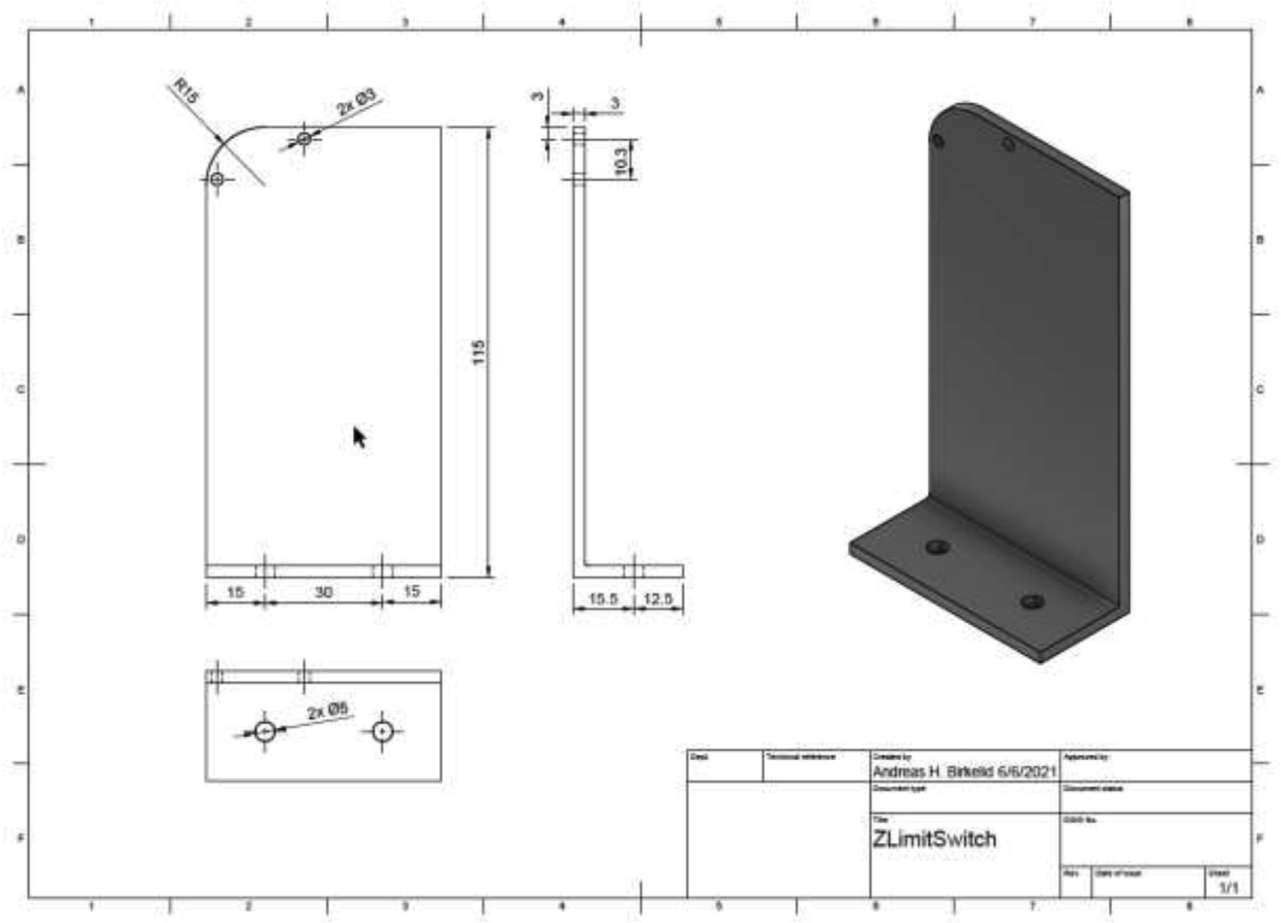
Z limit switch holder.

Create a Z limit switch holder by bending or welding a 3 mm steel/aluminum plate following the mechanical drawing in Fig. 63. This holder would work with both Volcano and SuperVolcano hotend; however, if one chose the Volcano, the height may be reduced to save material costs and decrease the time needed for homing.

**Fig. 64:**
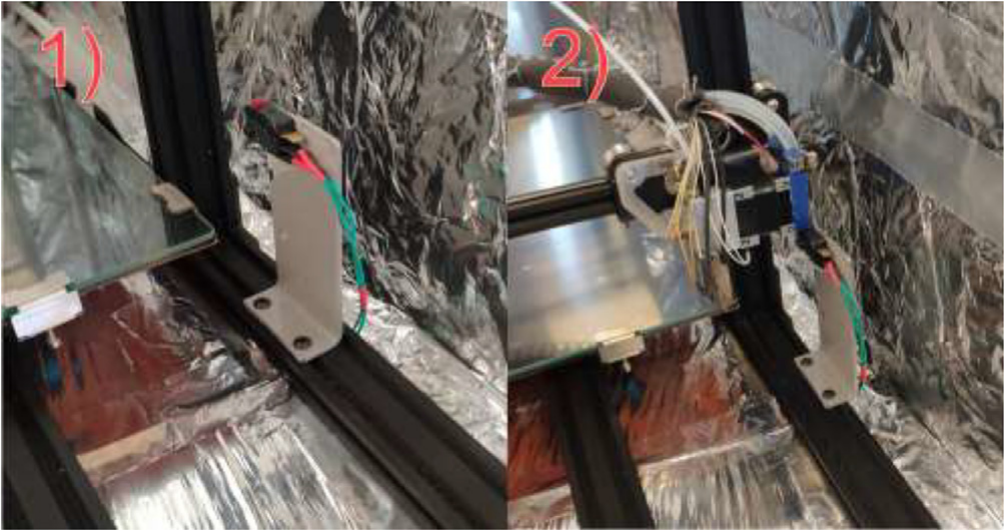
Z limit switch installed (left) and homing position (right).

Using 2x M3x16 mm, 2x M3 nuts, and 2x M3 spacers, mount the limit switch to the bracket using 2x M5x10mm and 2x M5 T-nuts mount the assembly to frame as in Fig. 64. Ensure that the extruder assembly can safely hit the limit switch to enable safe homing.

### Upgrading heated bed

**Fig. 65:**
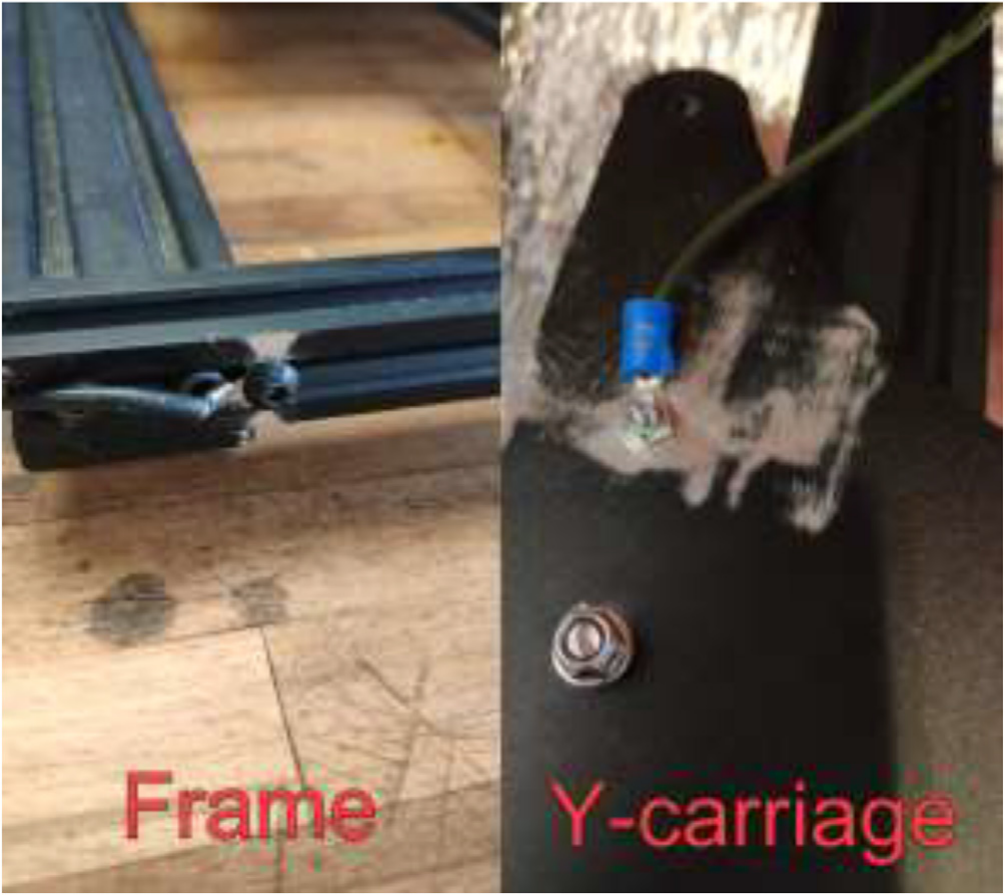
Grounding of frame and carriage.

High bed temperatures are generally needed for high-performance printing polymers. A 220 V AC-heated bed will replace a 12 V DC bed and provide temperatures up to 200 °C while also working as an additional heat source for the chamber. As AC power can be dangerous, it is crucial to ground the frame and carriage properly. Drill a hole in the carriage in the back left side, grind away the paint, and mount HRW to the Y-carriage, as seen in Fig. 65. Make sure that the screw does not interfere with the bed movements. In the front left of the frame, do the same procedure and use a T-nut and an M5x10 mm screw to mount another HRW grounding cable.

**Fig. 66:**
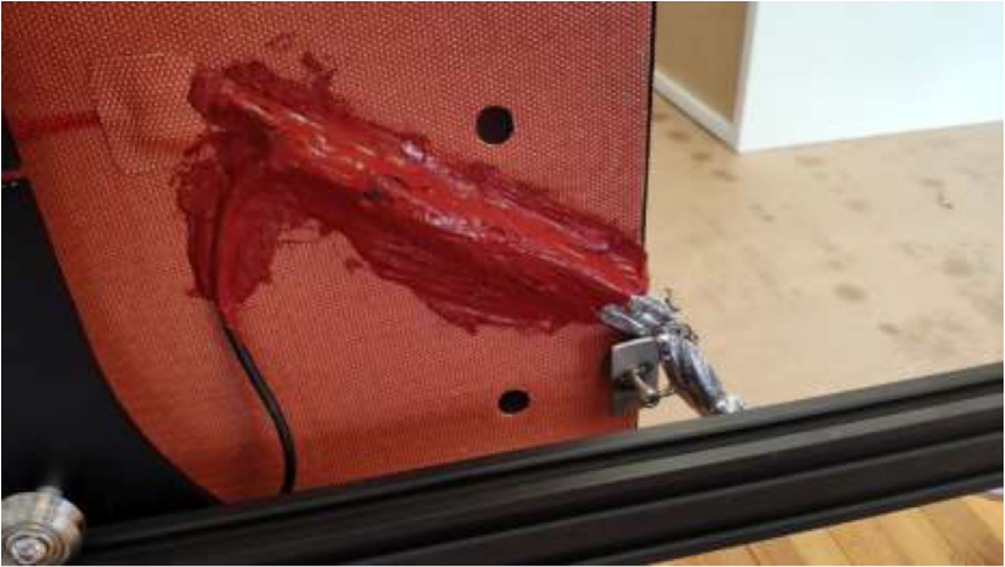
Strain relief for silicone heater using HTS.

To install a bed heater, start by removing cables and solder joints from the heated bed. Next, wash with isopropyl alcohol and mount silicone heater directly to the bed using the same procedure as the chamber heater. It is vital to ensure that no air bubbles are trapped under the heater and that the holes line up correctly. To provide strain relief to cables, the heater wires, thermistor wires, and the carriage grounding cables are fastened to the pad using HTS (Fig. 66).

**Fig. 67:**
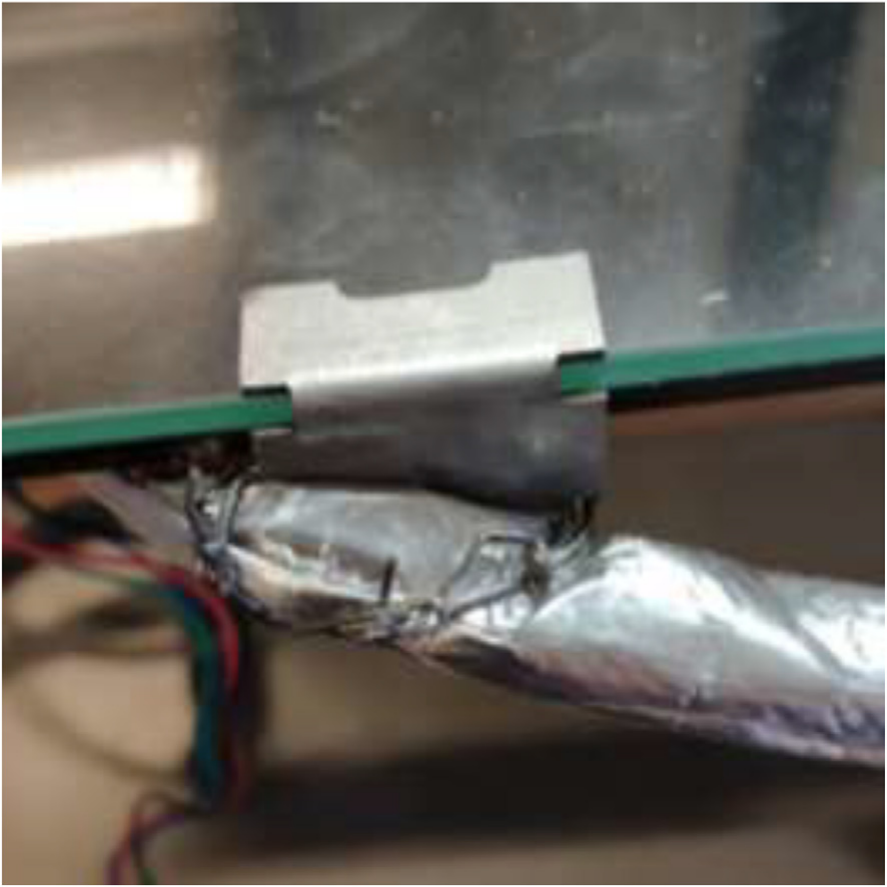
Y-axis strain relief using steel wire and glass bed holder.

Install new glass bed holders to each side of the glass, add 20 mm heat-resistant fiberglass sleeve and fasten cables into one of the glass bed holders (Fig. 67) to prevent live wires from rubbing into the frame and potentially short-circuiting the system.

**Fig. 68:**
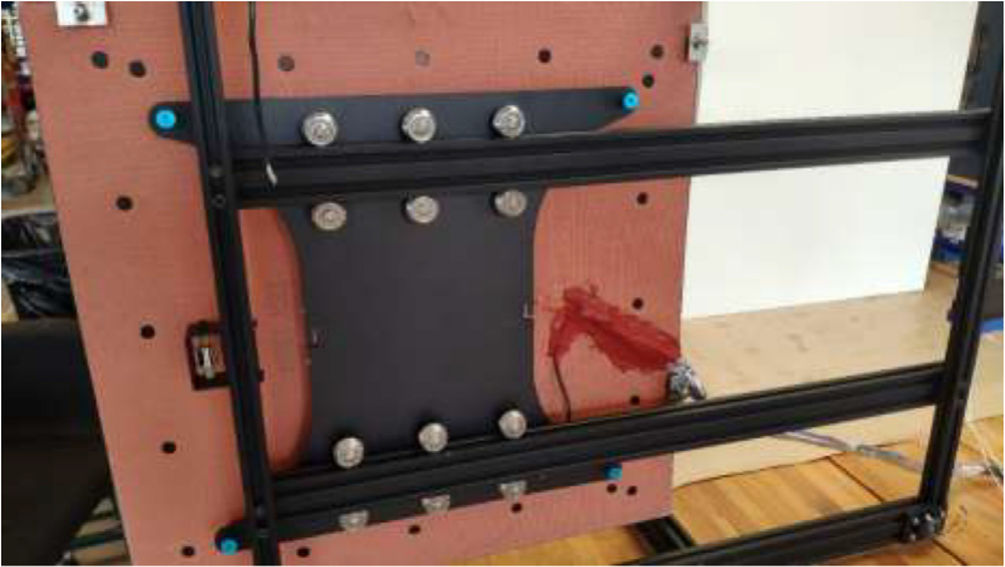
New heated bed.

Mount heated bed to the carriage and replace plastic bed leveling nuts with all-metal listed in the BOM. After assembly, it should look according to Fig. 68, however, with a belt and Y motor installed.

### Printer installation and electronics mounting

**Fig. 69:**
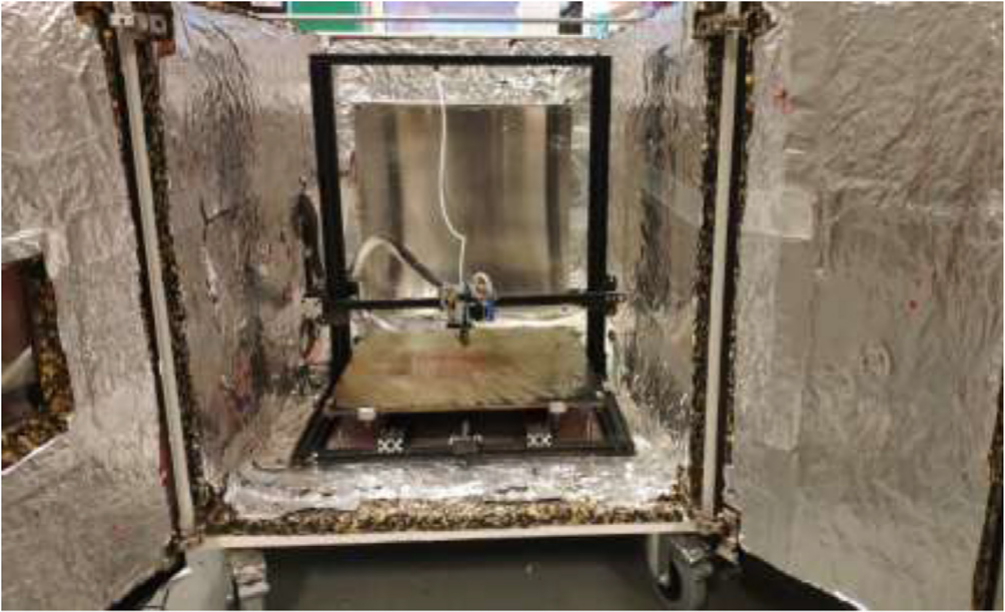
Illustration photo of printer placement in the chamber.

Check that all stepper motors, limit switches, the heated bed, and extruder are correctly installed. Now it is time to assemble the 3D printer inside the heated chamber, as shown in Fig. 69. Again, ensure that all wires are appropriately marked with their function and ensure that all axes can move freely.

**Fig. 70:**
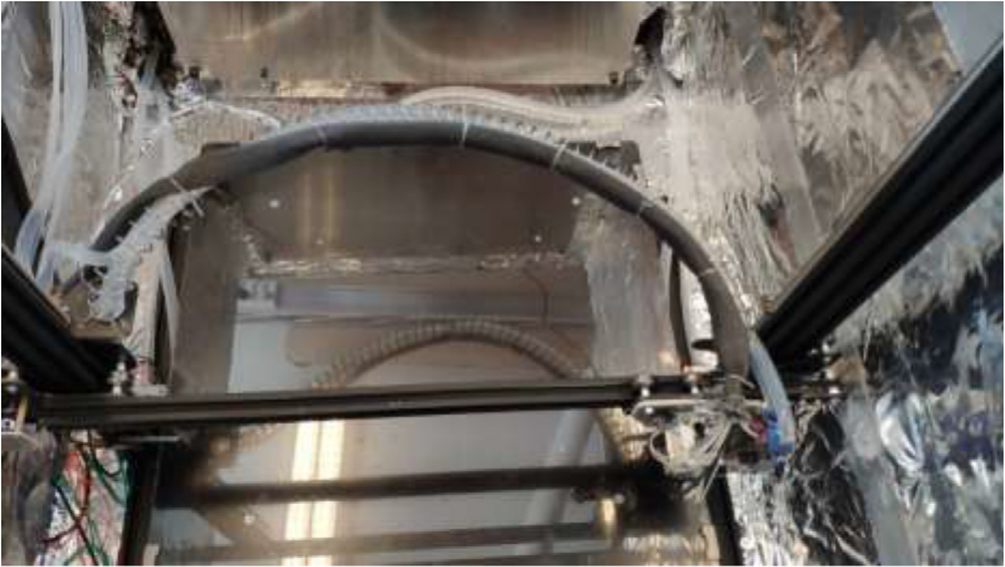
Extruder cable management.

Cut a piece of 20 mm braided fiberglass sleeve equal to the length of the spring. Insert all cables from the extruder through the sleeve and mount to spring using steel wire, as shown in Fig. 70.

**Fig. 71:**
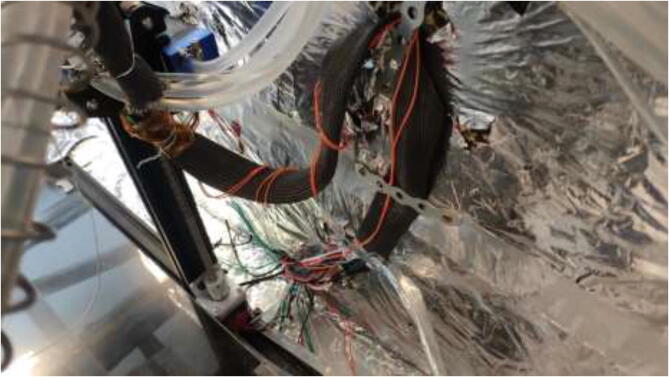
Cable management for X axis and extruder.

Pull all cables from the extruder and X-axis into a 1 m long braided sleeve and mount to the wall using steel bands and 3.5x20 mm wood screws as in Fig. 71. Make sure to provide enough length so that the X-axis can use the whole build volume. Use the same procedure to ensure that the cables coming from the heated bed do not interfere with the bed movements. Pull all cables through the hole created earlier in the design (Fig. 13).

**Fig. 72:**
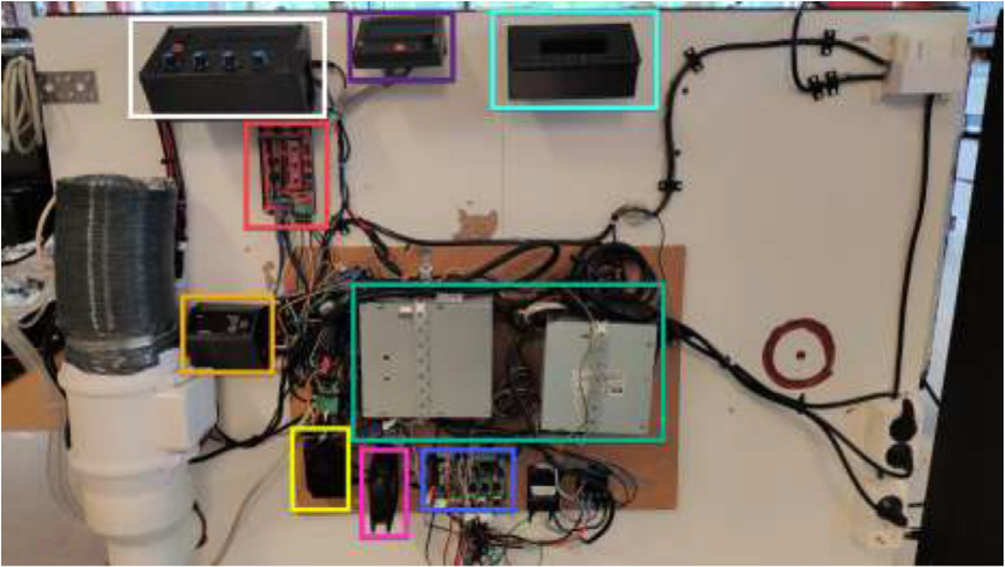
Electronic mounting. Liquid cooling control (white), Printer LCD (purple), Stepper temp monitoring (torquise), Ramps 1.4 (red), PID controller (orange), PSU’s (green), RPi 4 (yellow), Fan for mainboard (pink) and Mainboard (blue). (For interpretation of the references to color in this figure legend, the reader is referred to the web version of this article.)

**Fig. 73:**
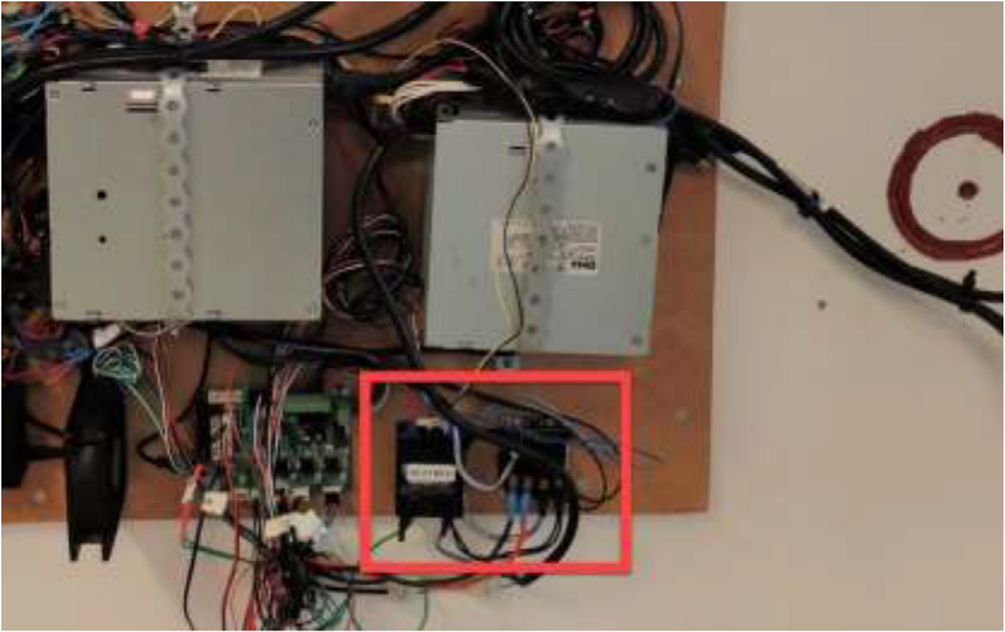
Bed SSR and hotend mosfet.

**Fig. 74:**
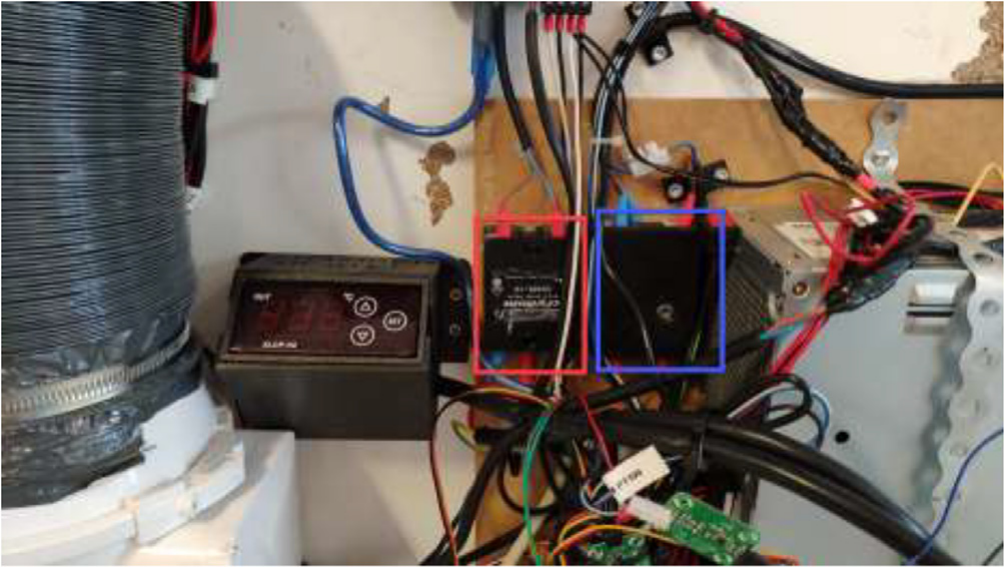
Exhaust fan and chamber heater SSR.

Print RAMPSHOLDER, RPI CASE, CoolingControlCase, CoolingControlCover, 3x relayCover, and optionally PID HOLDER/1x relay cover and LCD holder. Print CoolingControlCover and CoolingControlCase. Mount printed cases and printer components using screws, glue, and steel bands to the left side of the chamber. Examples of mounting configurations are shown in Fig. 72, Fig. 73, and Fig. 74.

**Fig. 75:**
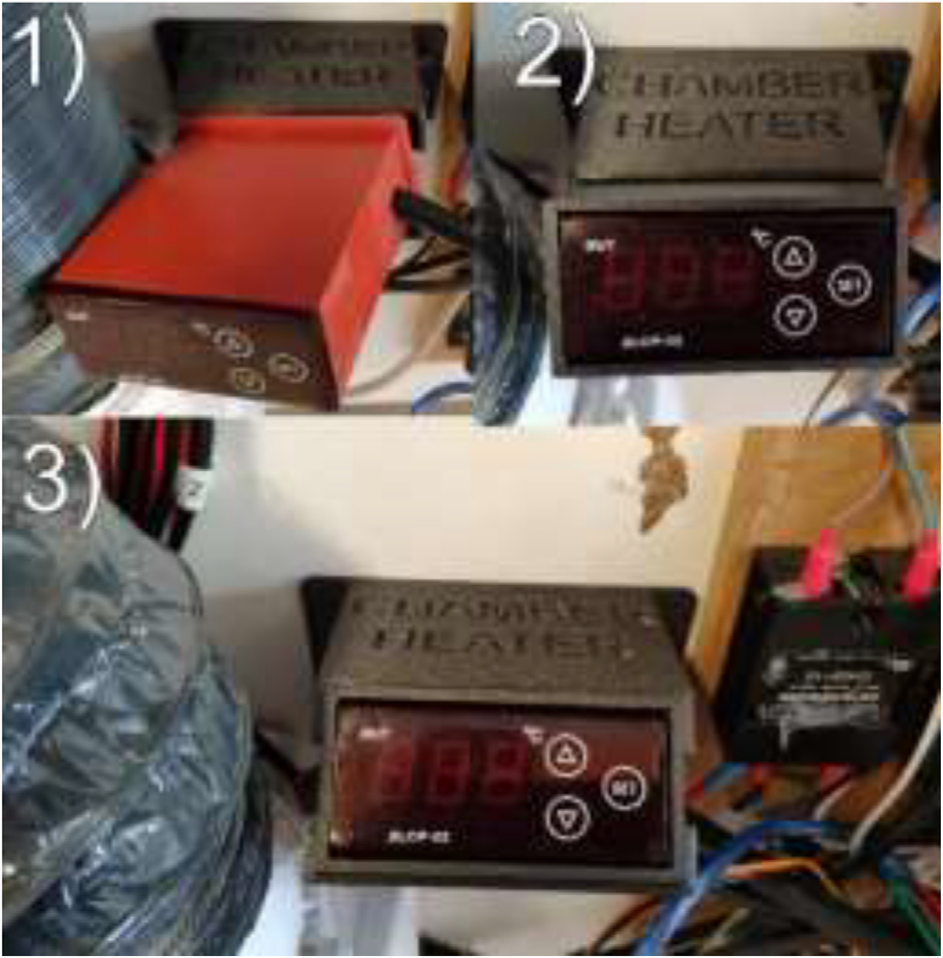
PID controller mounting.

If one has a chamber heater with an included PID controller, print ChamberHeaterBottom and ChamberHeaterTop. Mount as following Fig. 75.

**Fig. 76:**
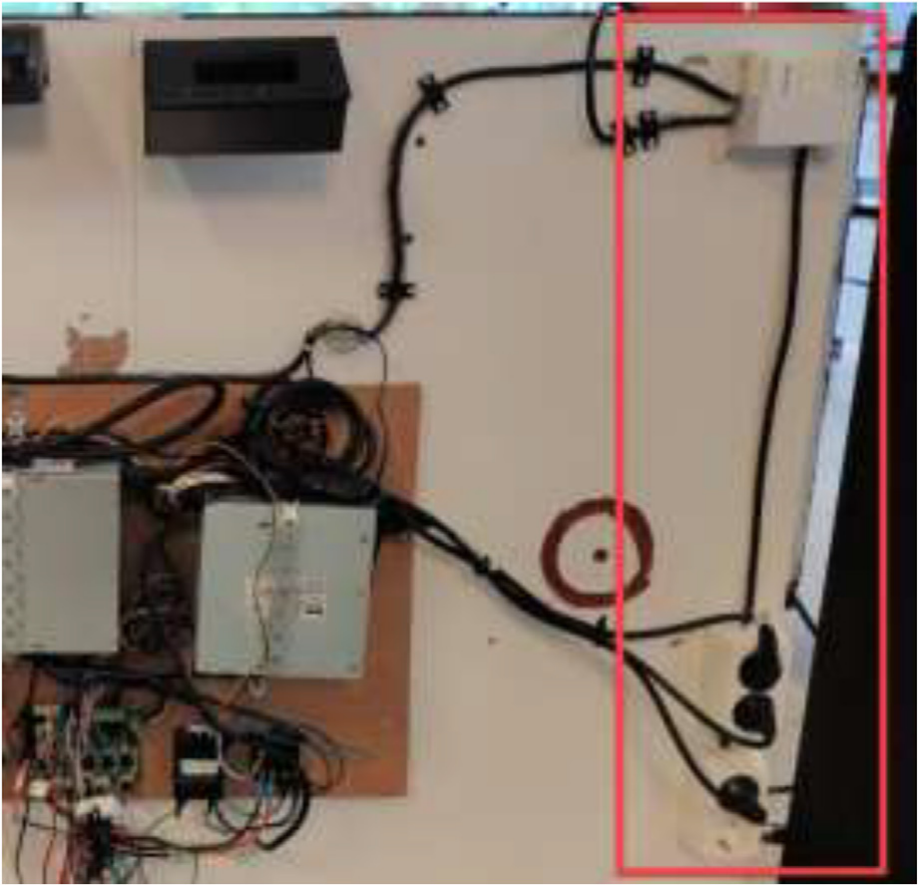
Optionally installation of switch and outlets.

Optionally, outlets and switches can be installed, as seen in Fig. 76, to provide AC power to the device. Wire according to the sheet provided by the manufacturer.

### Electronics wiring

**Fig. 77:**
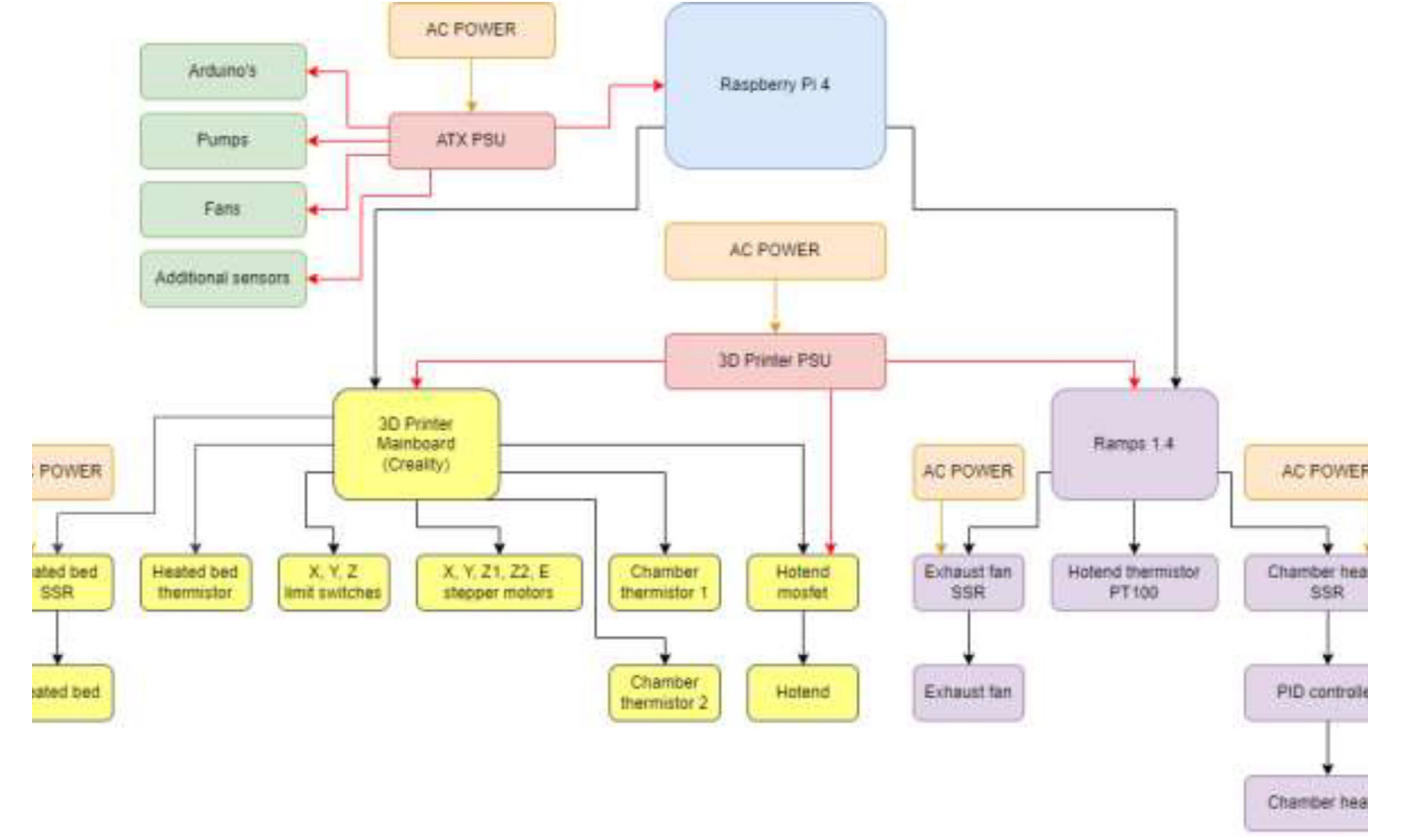
Connection diagram of the printer system.

The connection and communication diagram for the printer and its controlled environment is shown in Fig. 77.

**Fig. 78:**
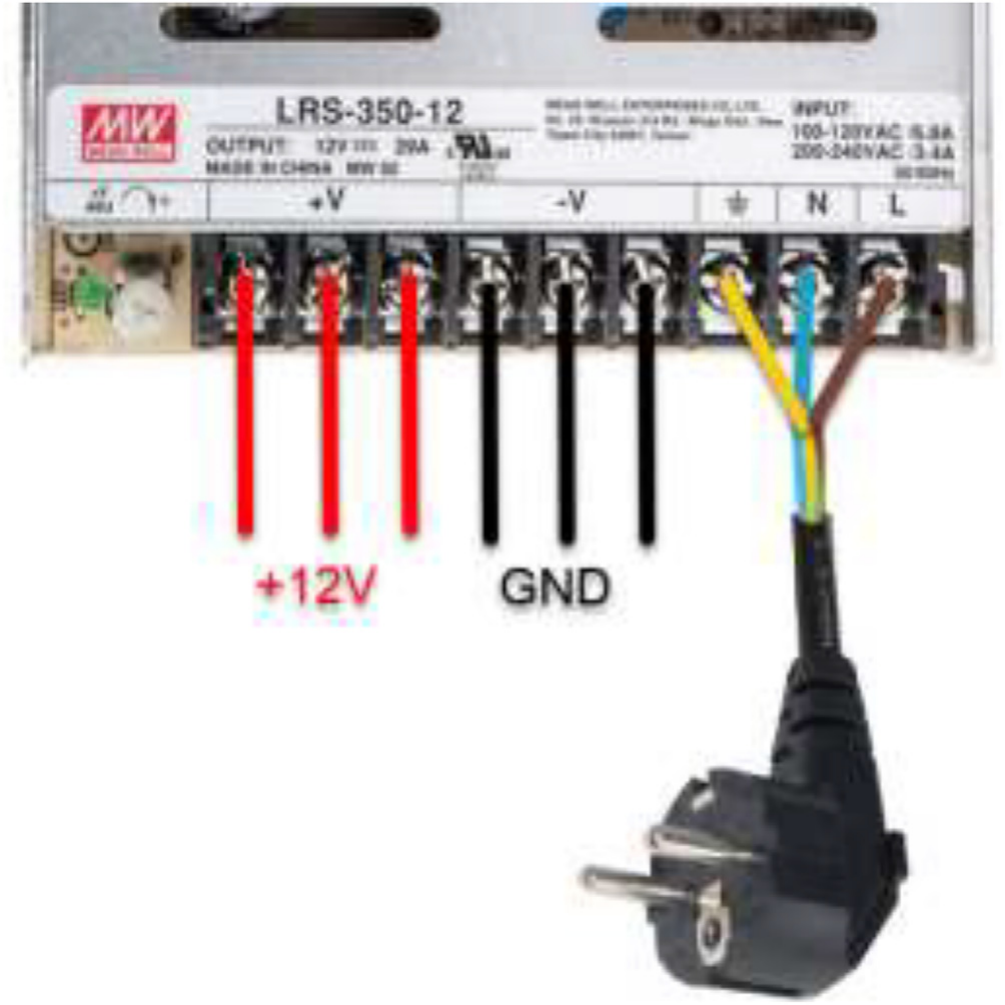
3D printer PSU wiring diagram [19], [20]

The original 3D printer PSU should be used to power the Creality Mainboard and the Ramps 1.4 shield. The original PSU included with the 3D printer is connected by following the markings on the power supply, as shown in Fig. 78.

**Fig. 79:**
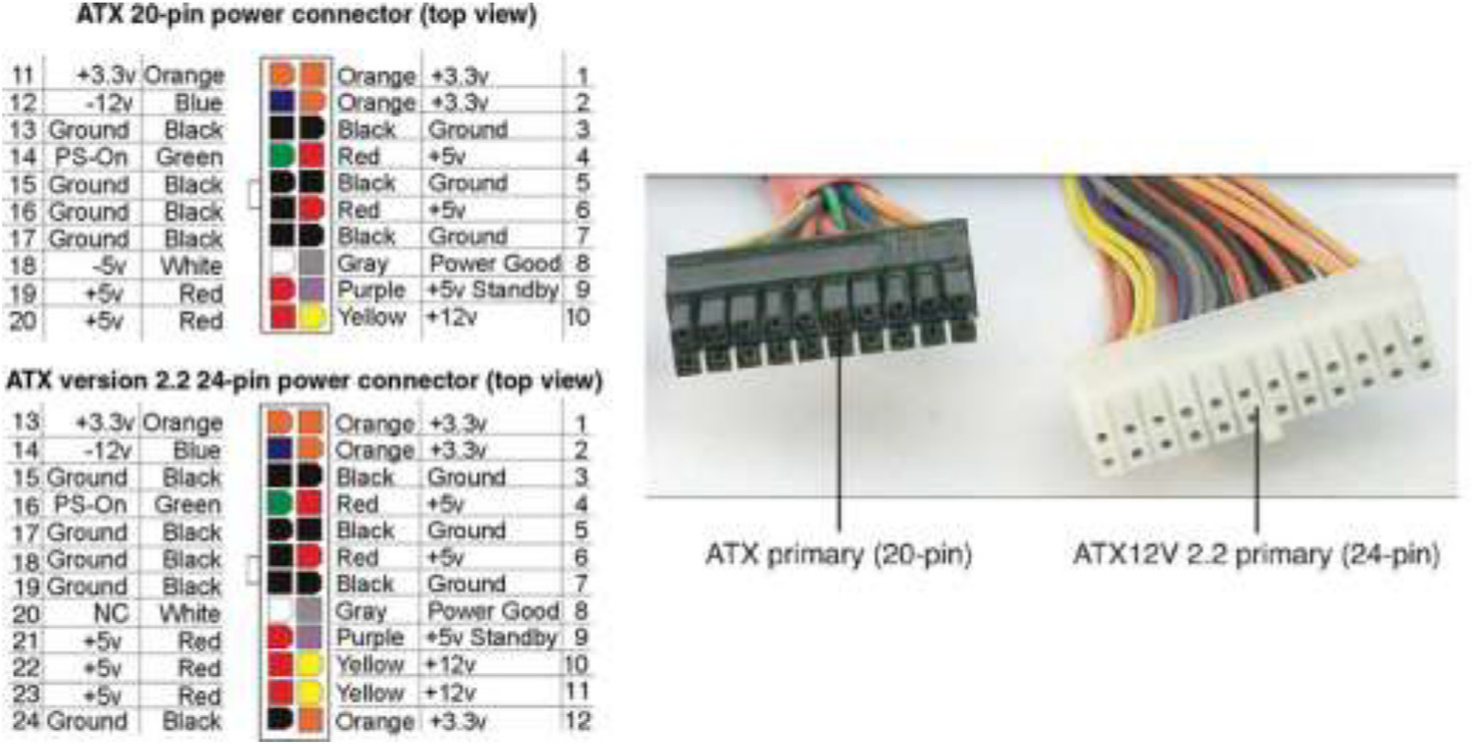
Color coding of most ATX-style PSUs [21]

The mounted ATX PSU power all external devices such as fans, pumps, Arduinos, and RPi. This style of PSU is widely available and inexpensive to obtain. They can also deliver both 12 V and 5 V, eliminating the need for buck converters. Fig. 79 explains the color coding for some of the most popular ATX PSUs. However, it is recommended to either check the documentation for the power supply or measure the voltage of each color against the ground using a multimeter to obtain the correct voltage as some PSUs’ color-coding may be different. Remove the large power connector, strip desired wires, extend cables if necessary and add suitable connectors. Connect the green wire to the ground by twisting together or soldering a switch to power up the PSU. Some PSUs need a 5 V load on startup and require a 10 Ohm resistor between 5 V and GND.

**Fig. 80:**
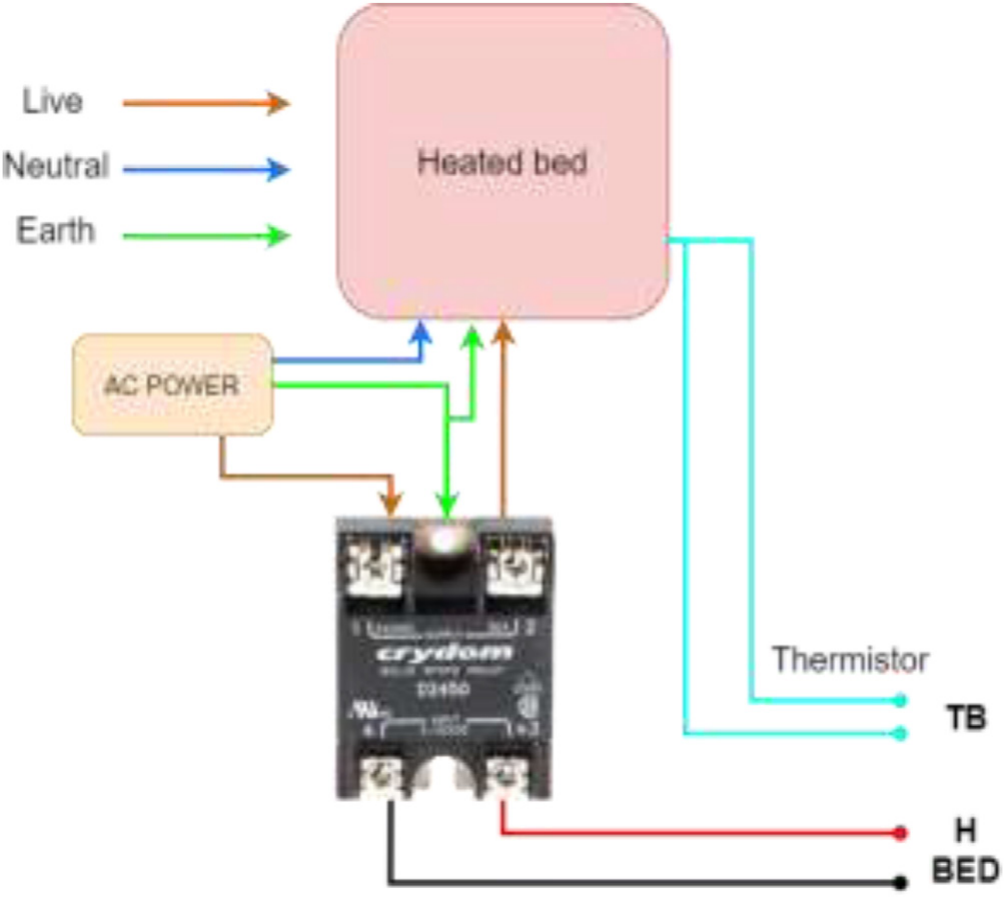
Wiring of silicone heater.

Start by wiring the heated bed, as shown in Fig. 80. This heater runs on AC and could potentially be deadly. Make sure that the grounding of the frame, SSR, and Y-carriage is appropriately installed.

**Fig. 81:**
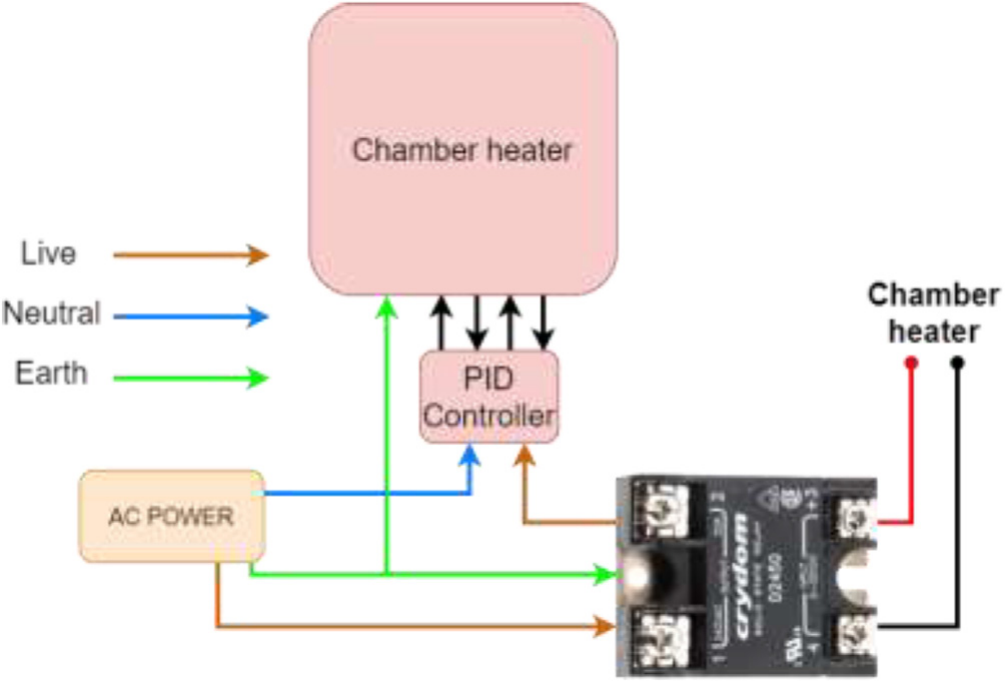
Chamber heater with PID controller.

If the chamber heater has an included PID controller, wire as following (Fig. 81).

**Fig. 82:**
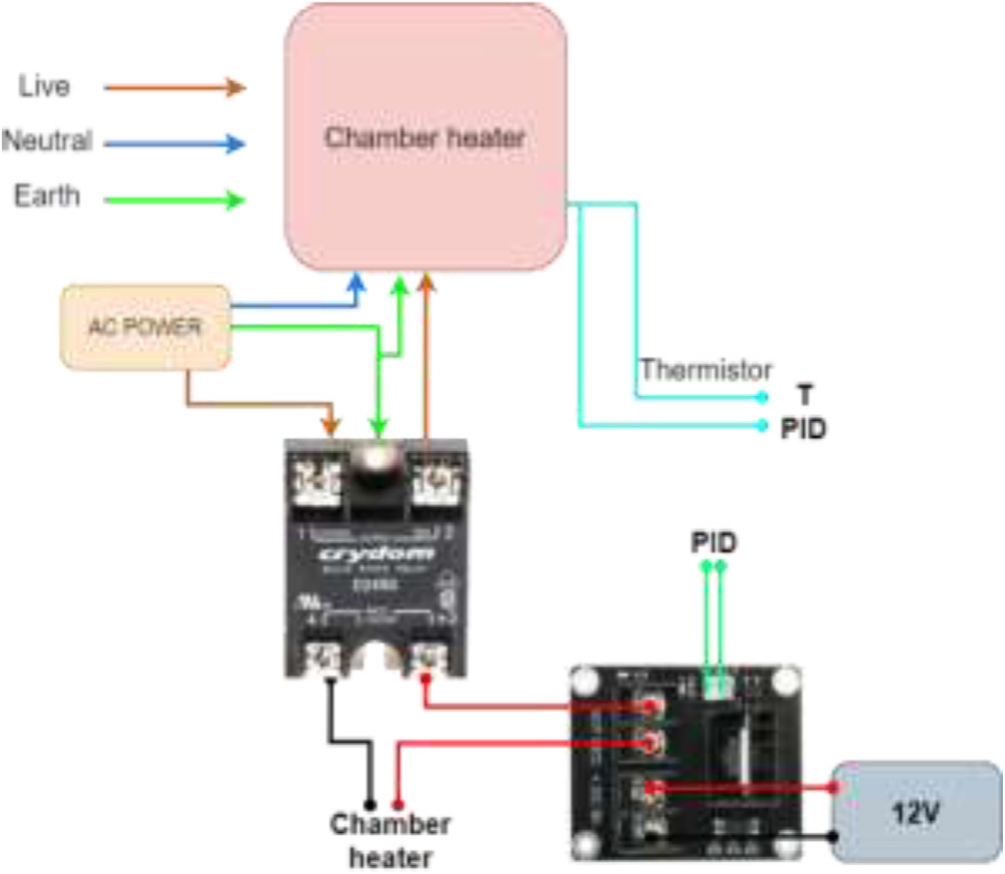
Chamber heater without included PID controller.

A chamber heater without included PID is wired according to Fig. 82.

**Fig. 83:**
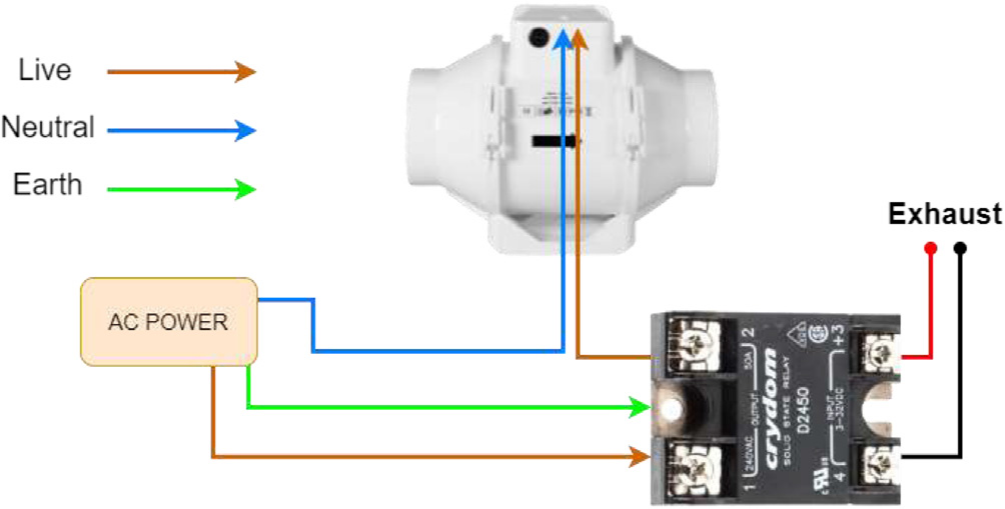
Exhaust fan wiring.

Wire the exhaust fan according to Fig. 83.

The hotend heater draws a relatively high current, especially on 12 V (∼7A for SuperVolcano), and it is vital to use an external mosfet to avoid burning the mainboard.

**Fig. 84:**
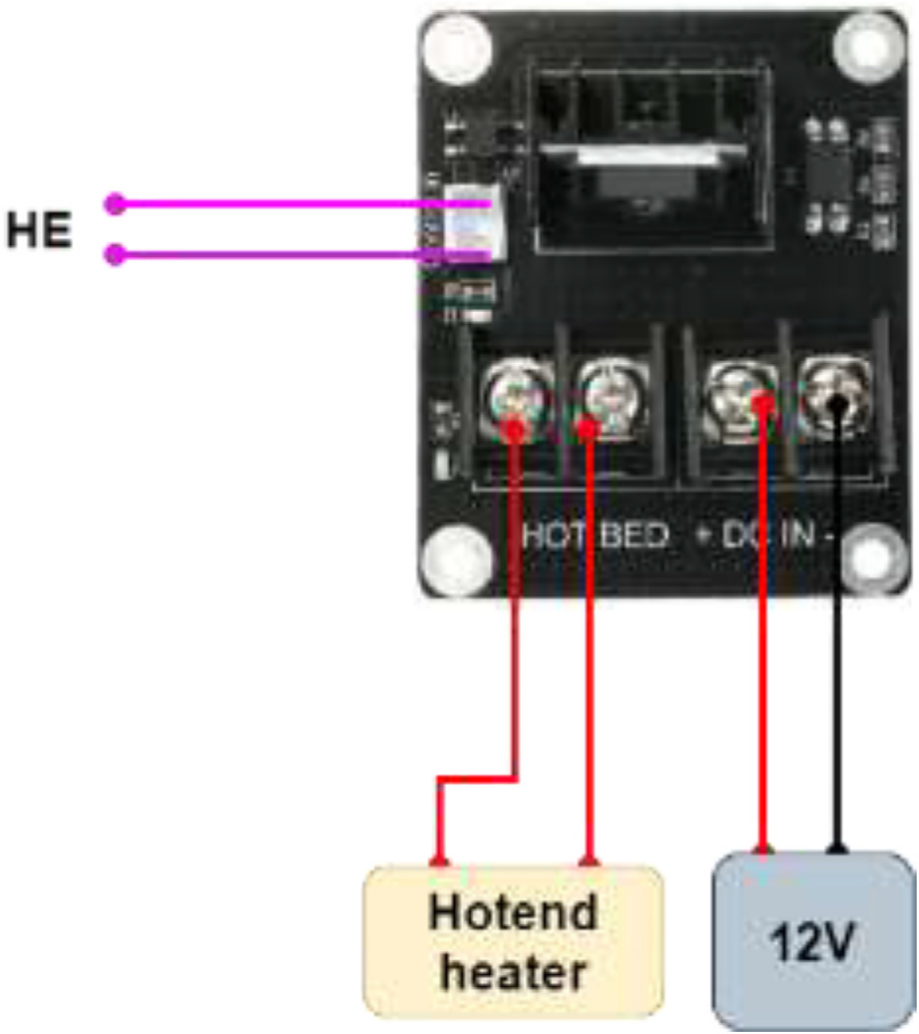
Extruder mosfet wiring.

Wire according to Fig. 84.

**Fig. 85:**
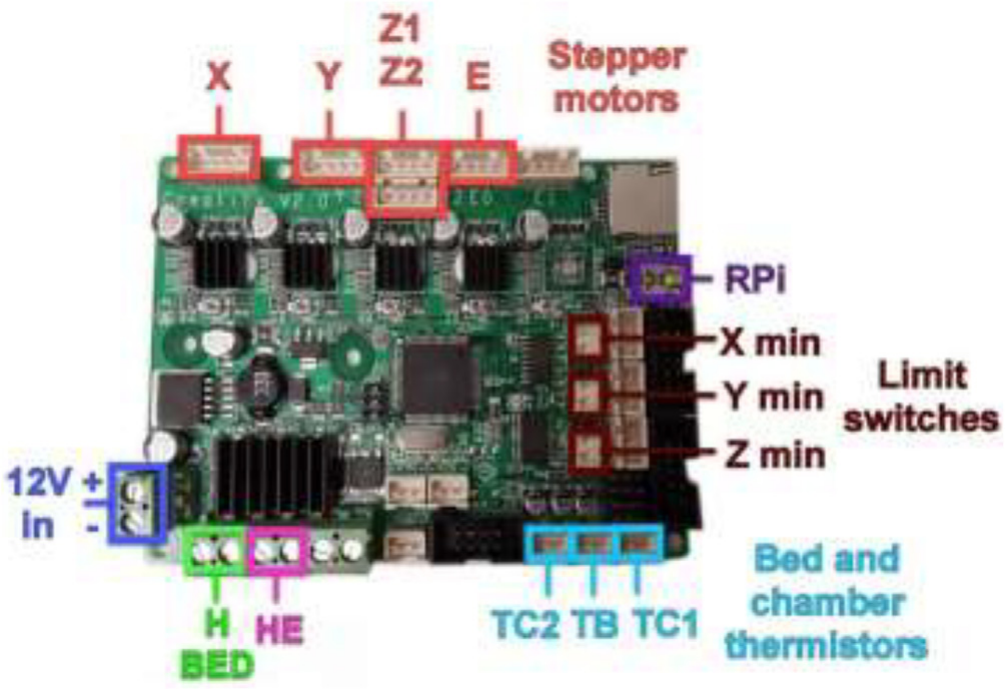
Wiring of original 3D printer mainboard [22]

Wire the mainboard according to Fig. 85 and connect the mainboard to RPi using the included USB cable.

**Fig. 86:**
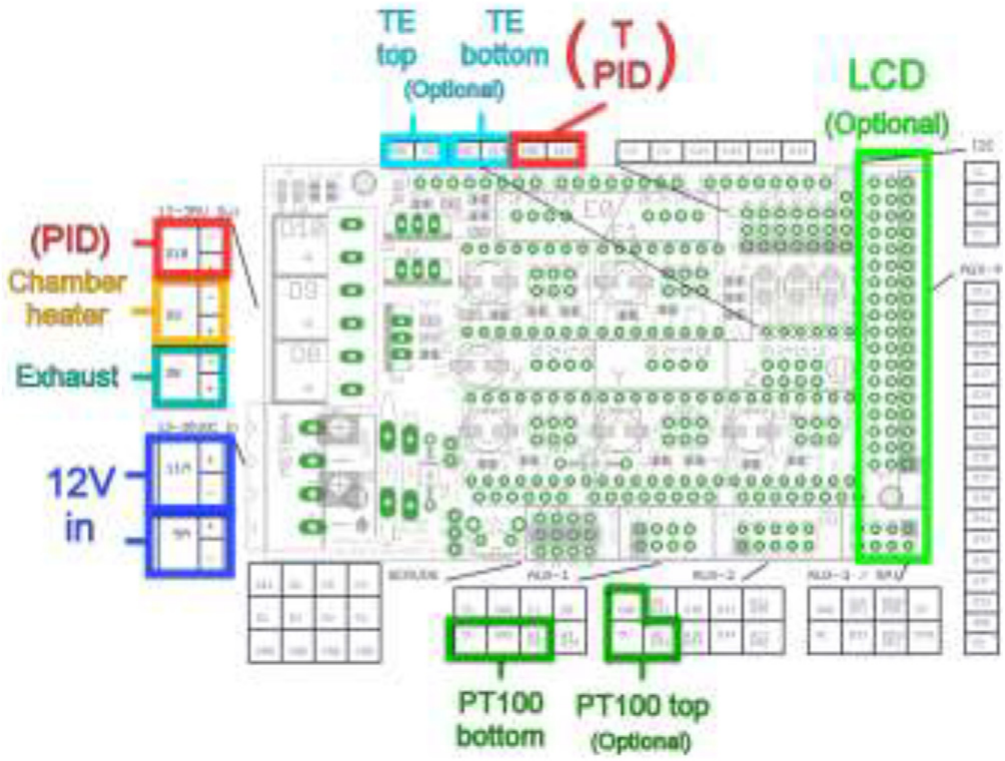
Ramps 1.4 wiring diagram [23]

Wire the rest of the components to the Ramps 1.4 board according to the diagram in Fig. 86. TE top and bottom are optionally extra thermistors added to the extruder either on top or on the cold side. PT100 sensor is not a thermistor and cannot be connected directly as regular thermistors. Connect to the included amplifier and follow the color coding, red to 5 V, black to GND, and yellow to analog input. LCD is optional and added using the included adapter. Connect Ramps to RPi using the included USB cable.

**Fig. 87:**
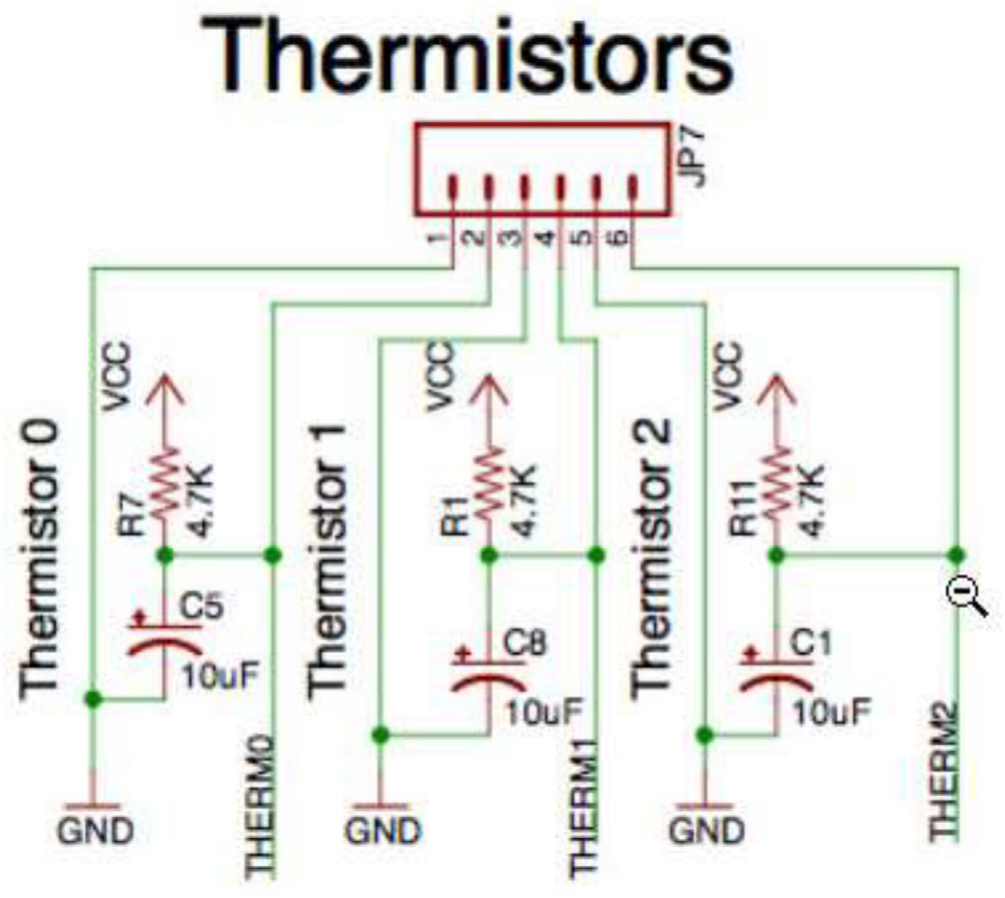
Adding more thermistors to board [24]

More thermistors can be added to vacant analog inputs. However, an adapter with a 4.7 K pull-up resistor and a capacitor with the following circuit is required to read it successfully (Fig. 87).

### Liquid-cooling electronics installation

**Fig. 88:**
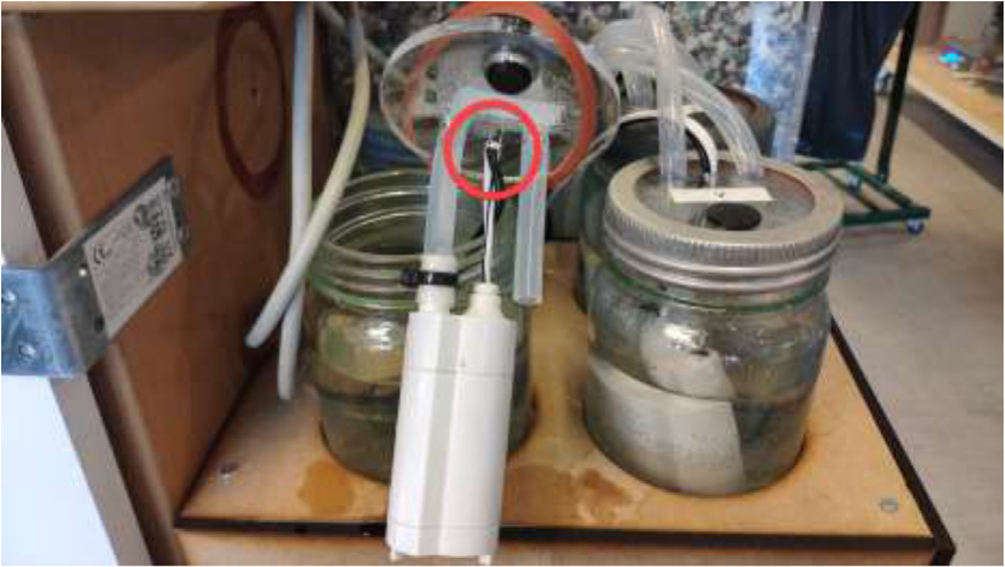
Pulling cables through cover.

The liquid-cooling electronics have a controller to adjust the speed of each pump. It also includes switches for turning all pumps and fans on/off. Optionally temperature sensors can be installed into each reservoir and monitored using an LCD. Insert one pump into each reservoir, optionally a DS18B20 temperature sensor, and pull all cables through the smallest hole in the cover, as seen in Fig. 88.

**Fig. 89:**
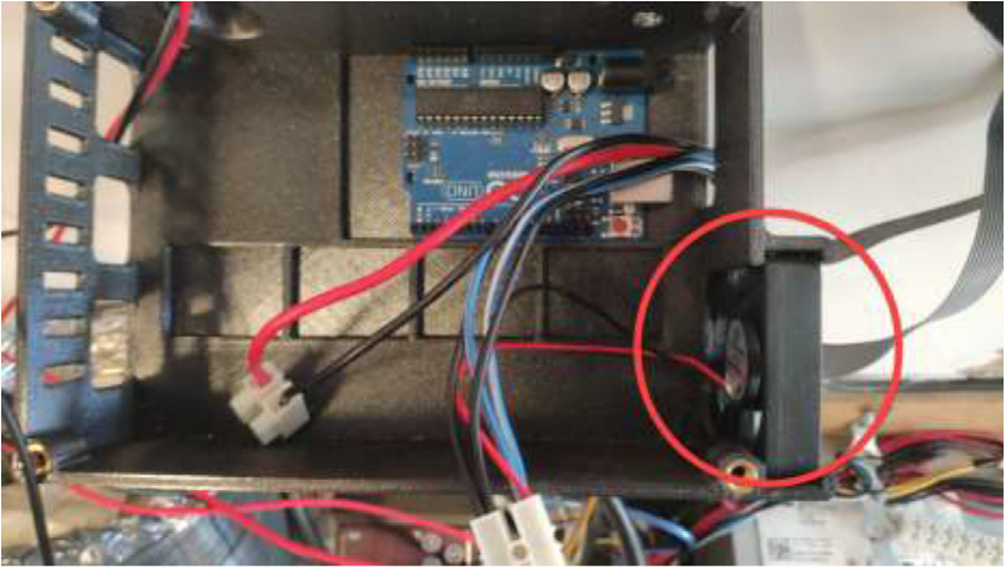
Fan install in pump control casing.

Insert one of the included fans from the original electronics enclosure as seen in Fig. 89 and connect to 12 V.

**Fig. 90:**
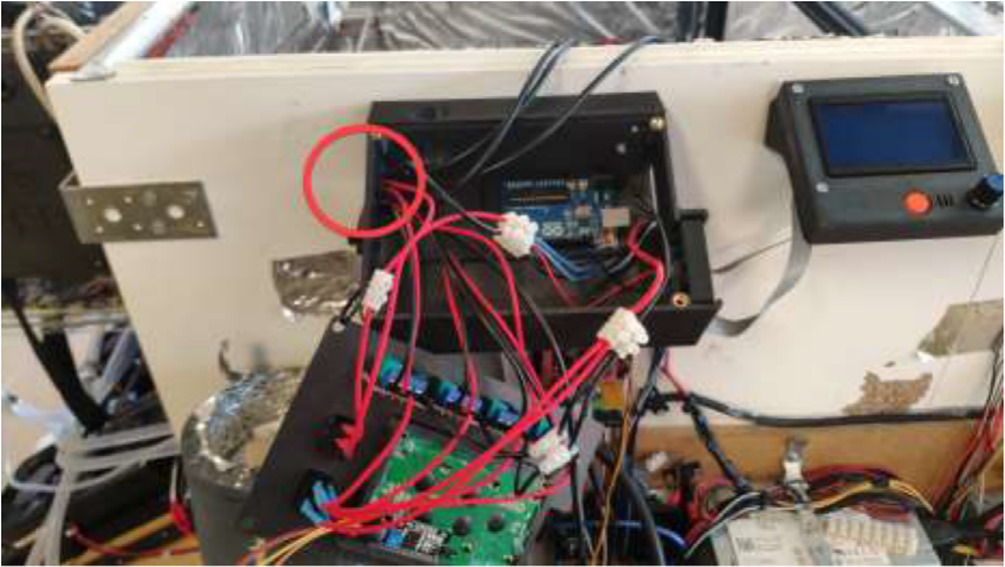
Pulling cables from motors and temperature sensors through casing inlet.

Pull cables from motors and optionally temperature sensors through the side of the case as seen in Fig. 90, extend these cables if necessary.

**Fig. 91:**
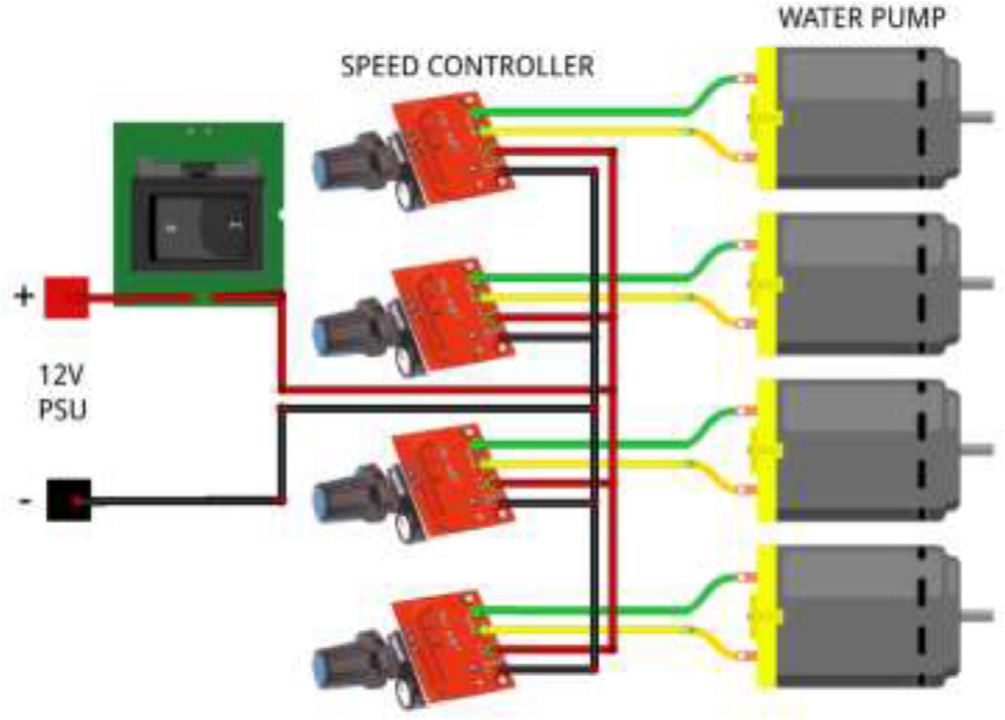
Liquid pump speed control.

Wire pumps, according to Fig. 91. Use 12 V from the ATX PSU.

**Fig. 92:**
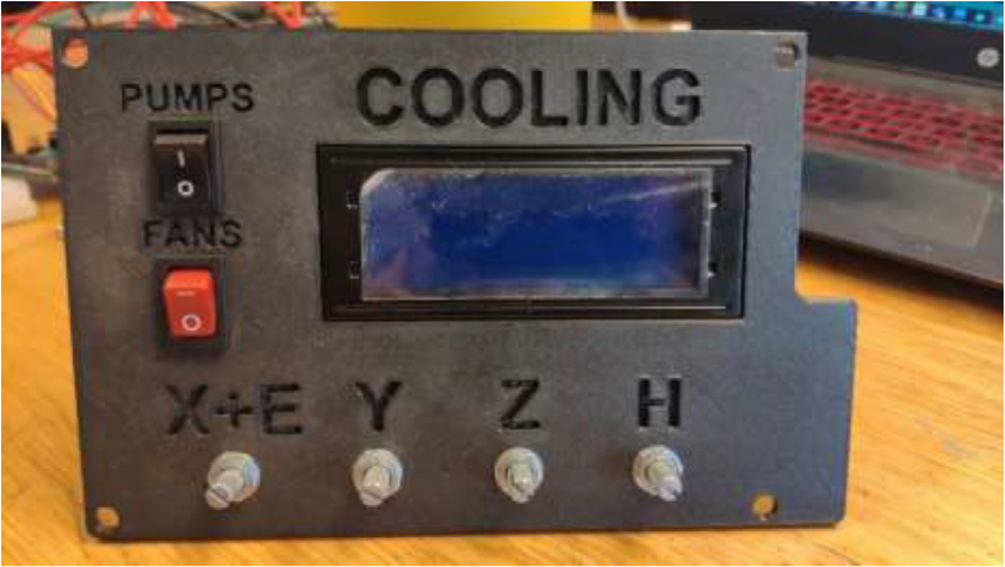
Cooling cover installation.

Insert speed controllers and switches into CoolingControlCover as seen in Fig. 92.

**Fig. 93:**
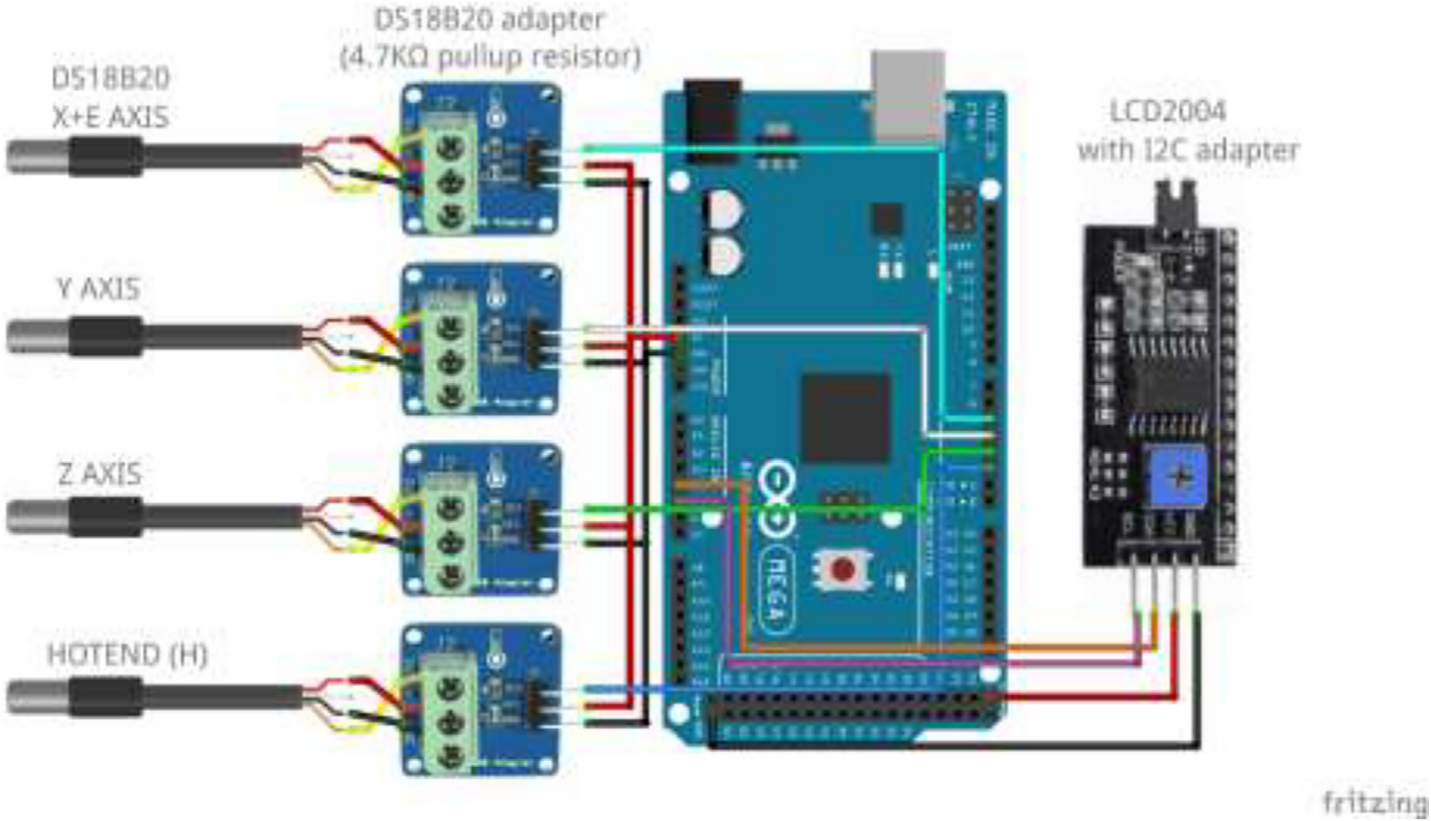
Liquid temperature measurement wiring diagram.

If optional temperature sensors for liquid are installed, connect the I2C adapter to LCD and use either Arduino Mega or Uno with DS18B20 temperature sensors and adapters. Wire as shown in Fig. 93 and insert LCD to CoolingControlCover.

**Fig. 94:**
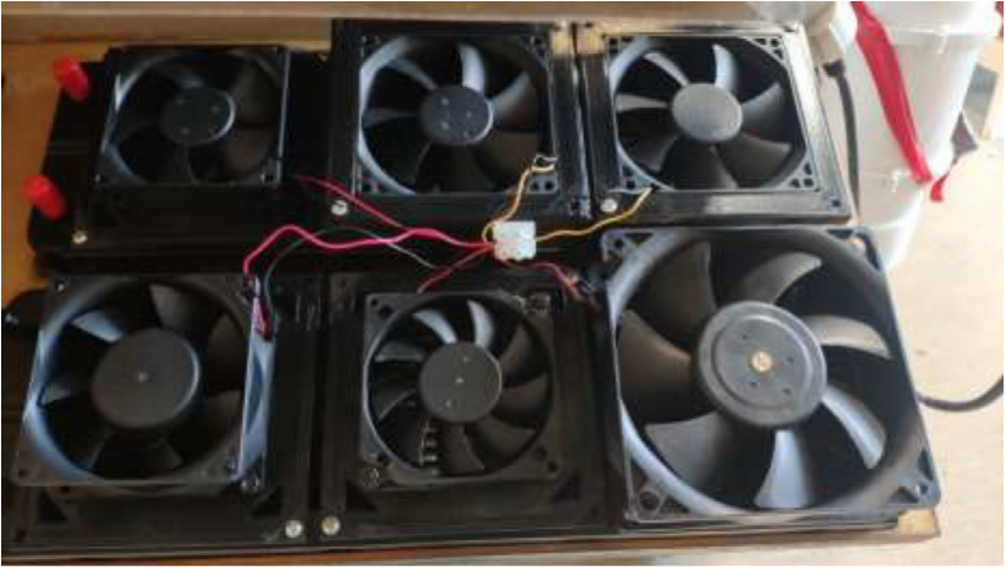
Fan connection.

Mount fans to radiators using the provided screws. If one did obtain smaller than 120 mm fans, adapters can be found online and printed [25], [26]. Connect all fans in parallel using screwable connectors or solder connections, as seen in Fig. 94.

**Fig. 95:**
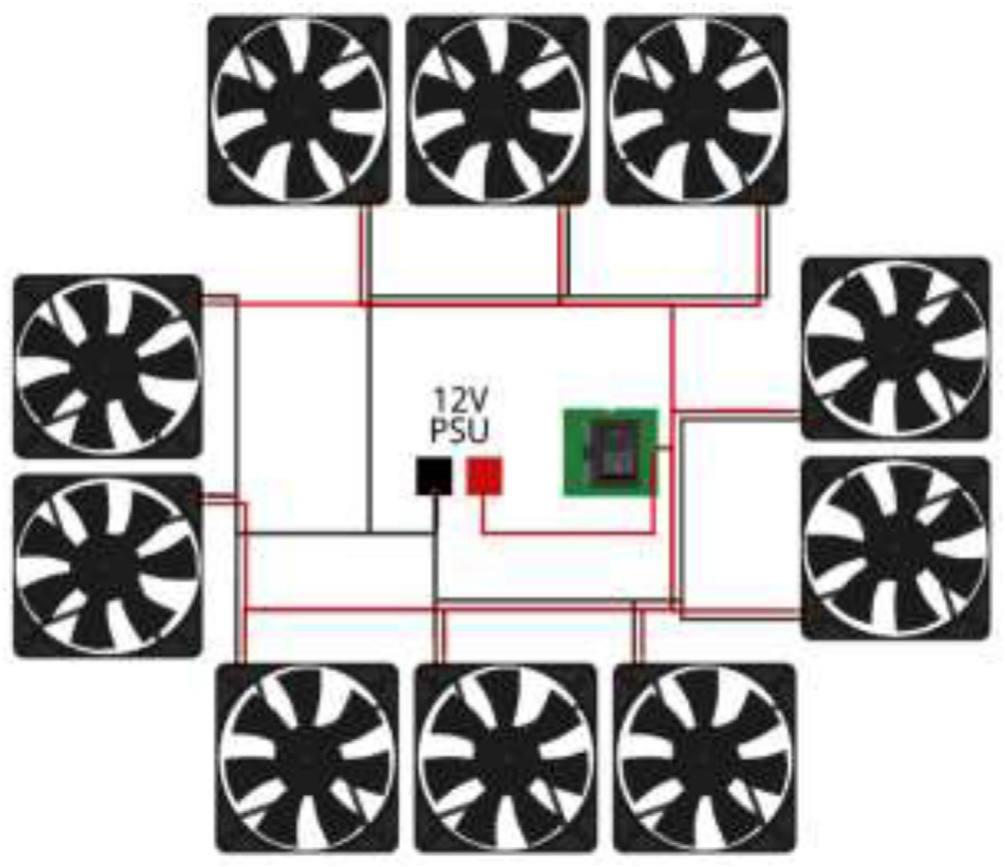
Radiator fans wiring diagram.

Pull cables through CoolingCase and wire according to Fig. 95.

### Liquid cooling loop configuration

**Fig. 96:**
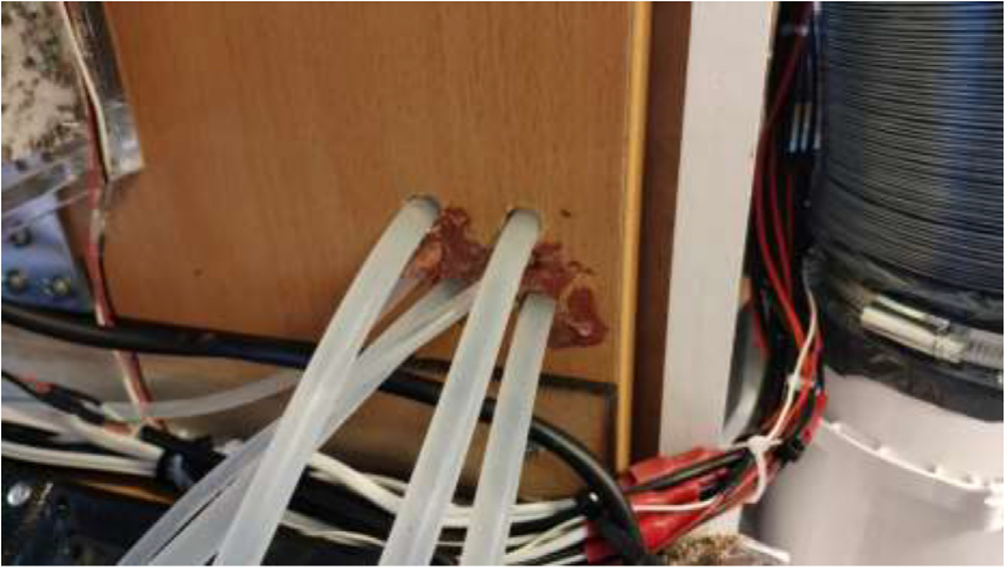
Holes for tubing.

This section will explain the loop configuration of each axis. The tubing length is not set directly and depends on the user’s configuration. Measure the needed size manually and cut where desired. Start by placing all radiators in the radiator holder. Make two 6 mm and four 10 mm holes in the middle right of the heated chamber, as seen in Fig. 96.

**Fig. 97:**
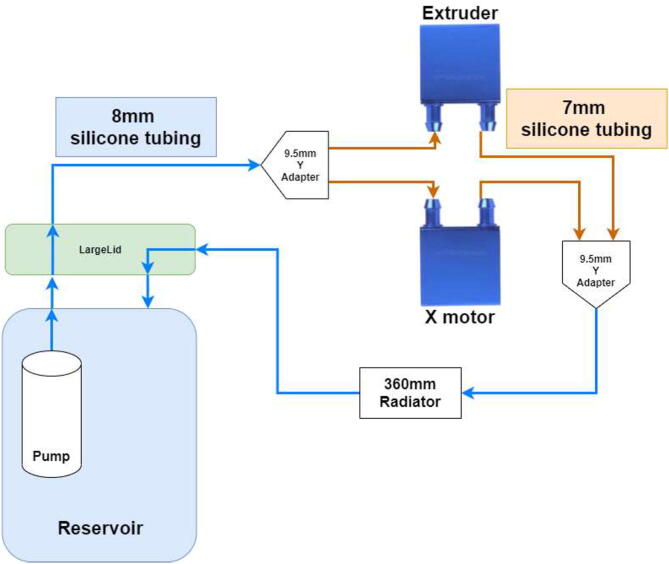
X and E axis cooling loop.

Firstly, the cooling system for the X and E axis will be installed. The tubing diagram is shown in Fig. 97.

**Fig. 98:**
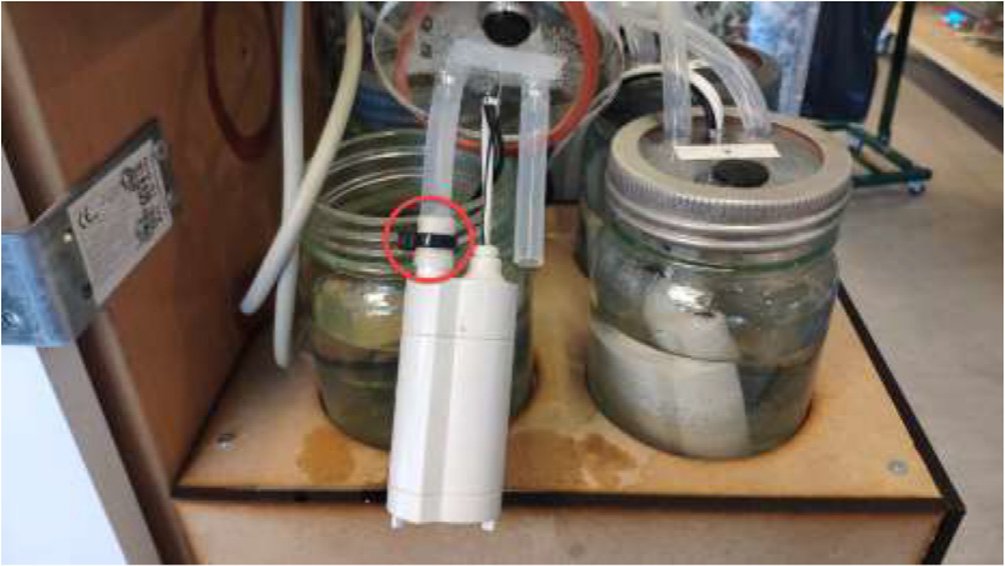
Tube mounted using zip-tie to pump.

Start by pulling 8 mm tubing through the lid and connecting to the pump using a zip tie as in Fig. 98.

**Fig. 99:**
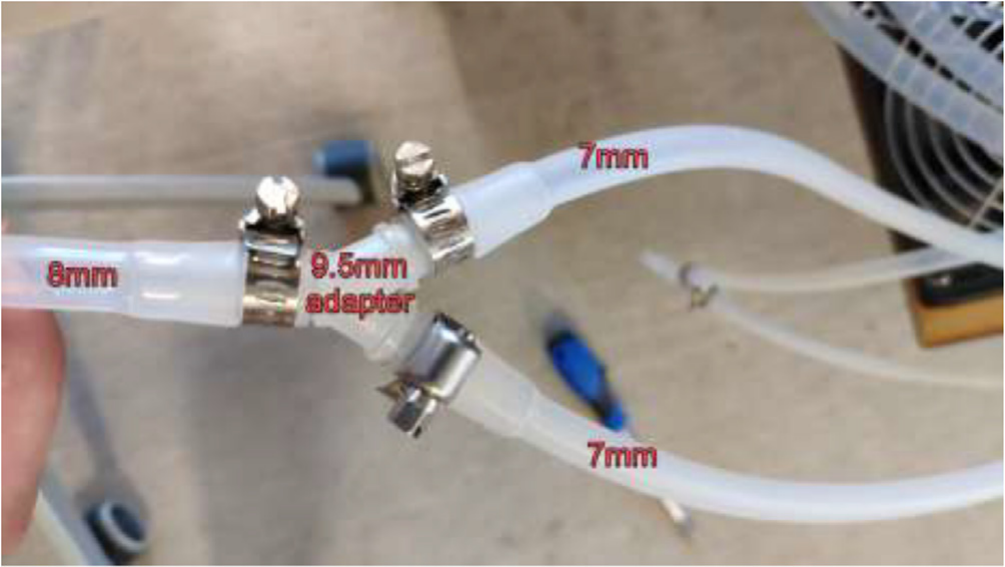
Silicone tube Y adapter, hose clamps and silicone tube.

Connect a splitter to the other end of the tube on the outside of the chamber. Connect two pieces of 7 mm tubing and use a hose clamp on all sides (Fig. 99).

**Fig. 100:**
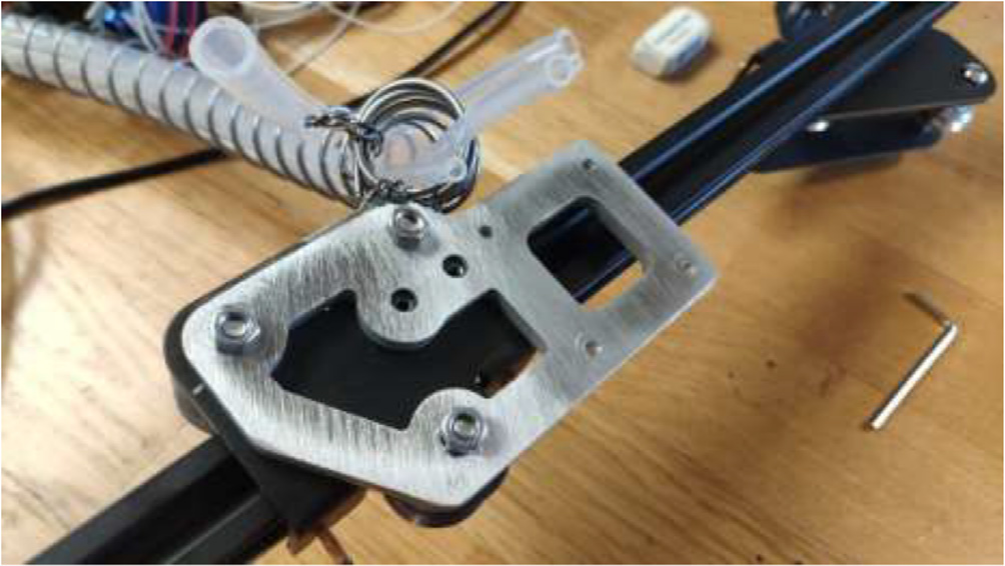
Tubing through spring.

Pull each tube through the backplate using the newly created holes. One tube is connected to the X motor block; the other is pulled through the spring, as seen in Fig. 100.

**Fig. 101:**
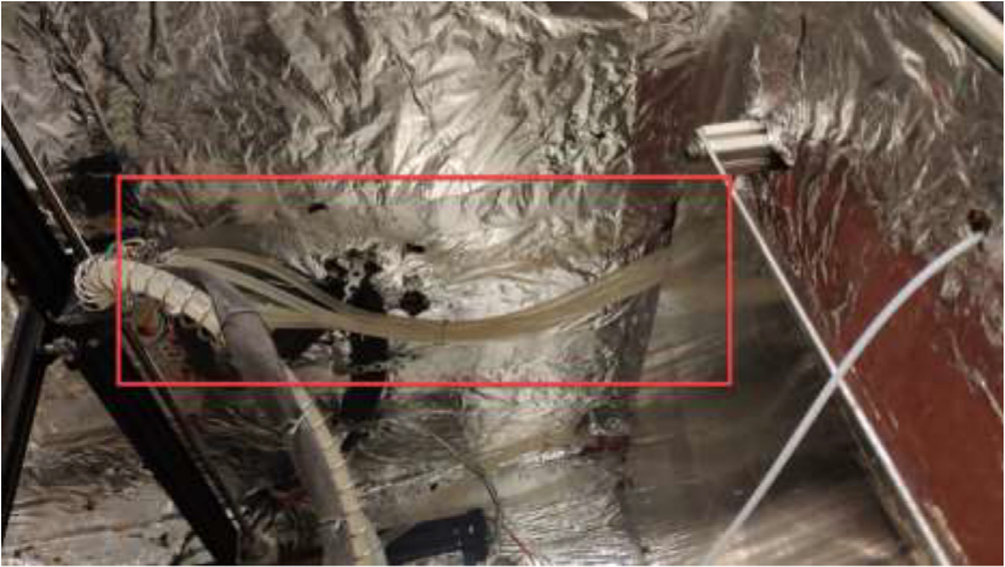
Tubing for X and E- axis.

Cut the tube at an appropriate length such that there is some slack in the tubing so the X-axis can move up and down as seen in Fig. 101.

**Fig. 102:**
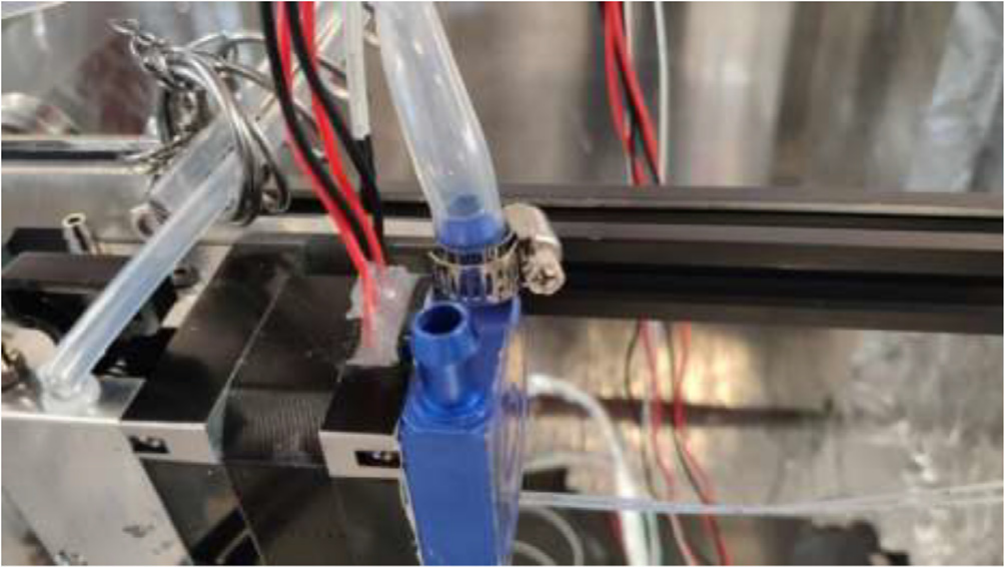
Tube installed on extruder stepper-motor.

Connect the tube to the extruder motor (Fig. 102) by using hose clamps.

**Fig. 103:**
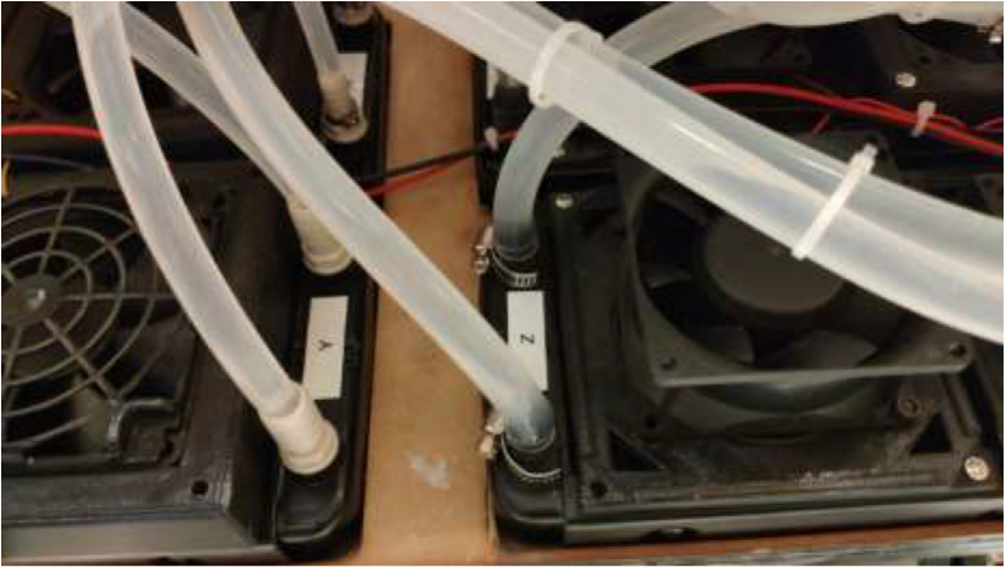
Tubes installed on radiators.

Add 7 mm tubes to the other side of each cooling block and pull the other way through the backplate. Next, connect each hose to a Y-adapter, as shown in Fig. 99, and connect the 8 mm hose directly to a 360 mm radiator (Fig. 103). The other side of the radiator has another 8 mm tube connected, pulled back into the reservoir.

**Fig. 104:**
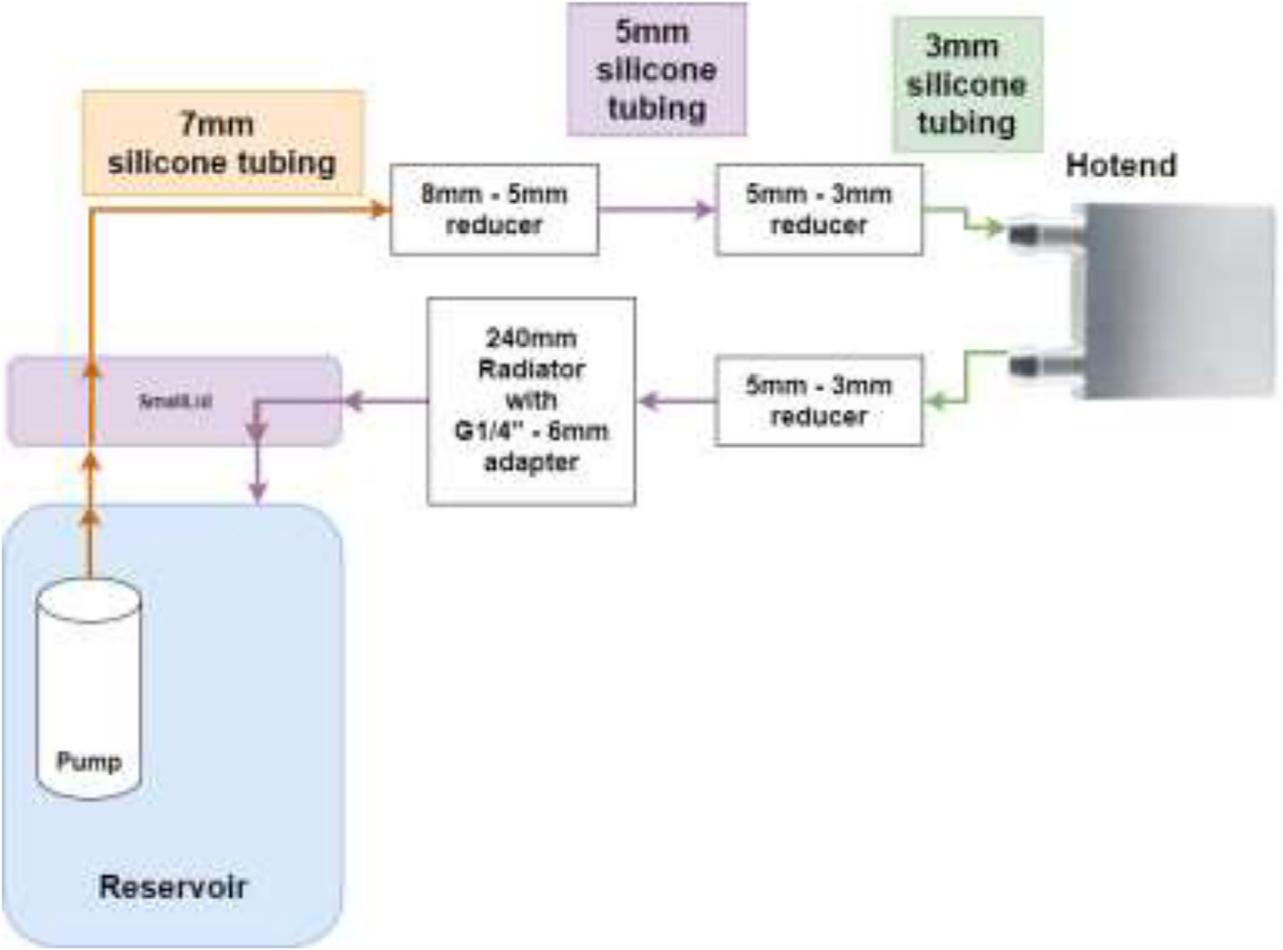
Hotend tubing diagram.

The next step is to install hotend cooling; the tubing diagram is shown in Fig. 104.

**Fig. 105:**
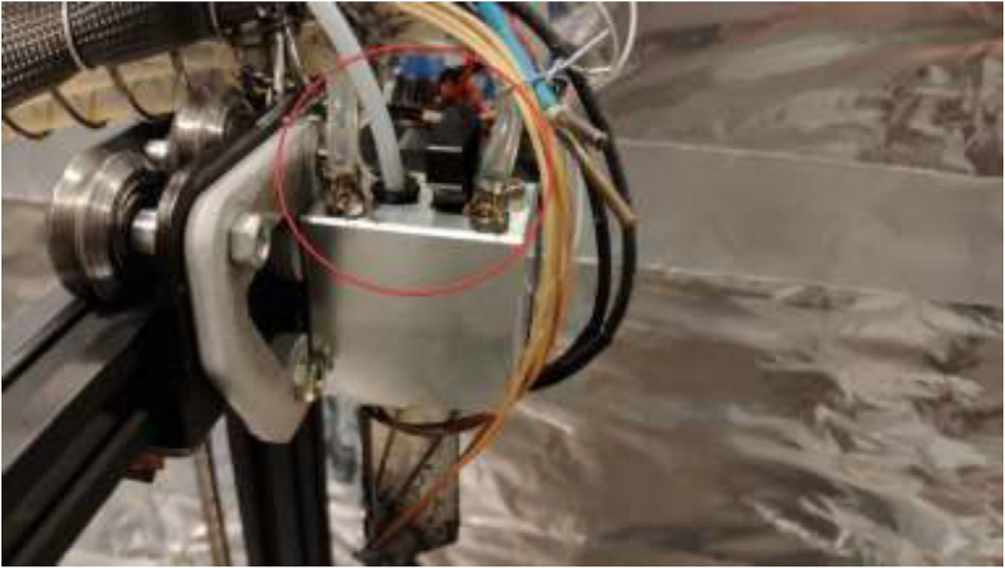
Hotend tubing connection.

Proceed to pull 5 mm tubing through holes in the backplate and spring as done with the E-axis. Connect tubing using the adapters provided in BOM and follow the tubing diagram for hotend in Fig. 104: Hotend tubing diagram. Ensure that hose clamps are used where necessary and that all connections are appropriately mounted to avoid leakage (Fig. 105).

**Fig. 106:**
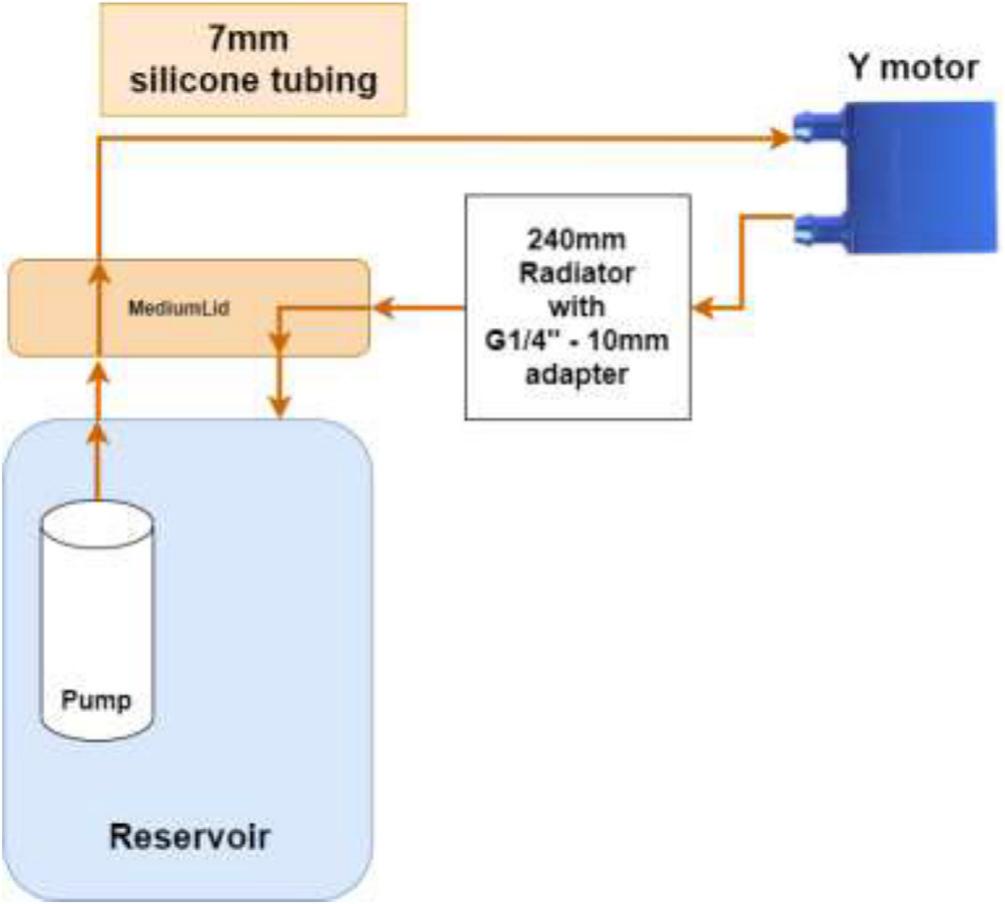
Y stepper motor pump connection diagram.

The loop order diagram for the Y-axis is shown in Fig. 106.

**Fig. 107:**
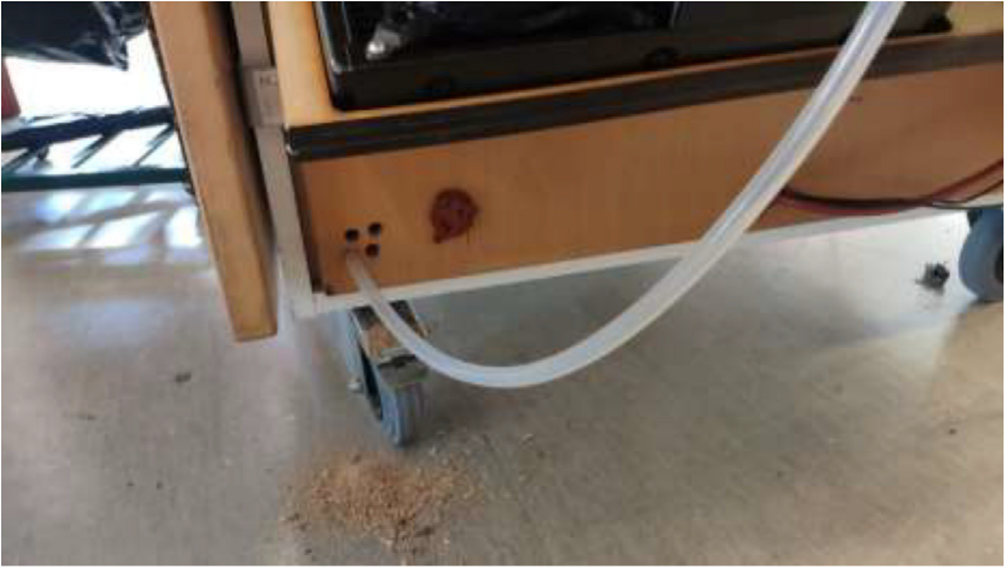
Holes for tubing to Y and Z motor.

Make four 10 mm holes by drilling the bottom right of the backplate, as in Fig. 107.

**Fig. 108:**
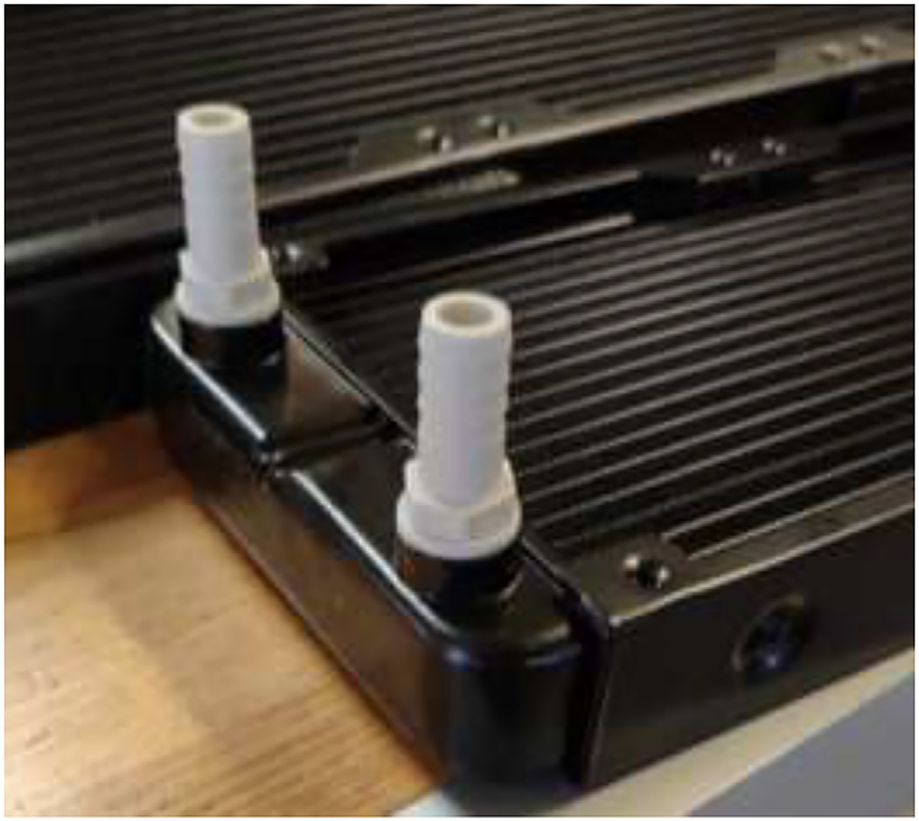
G1/4″ adapter installation.

Proceed to pull tubes and connect according to Fig. 106. G1/4″ adapter is installed as in Fig. 108. Make sure that it is tightly mounted to avoid leakage.

**Fig. 109:**
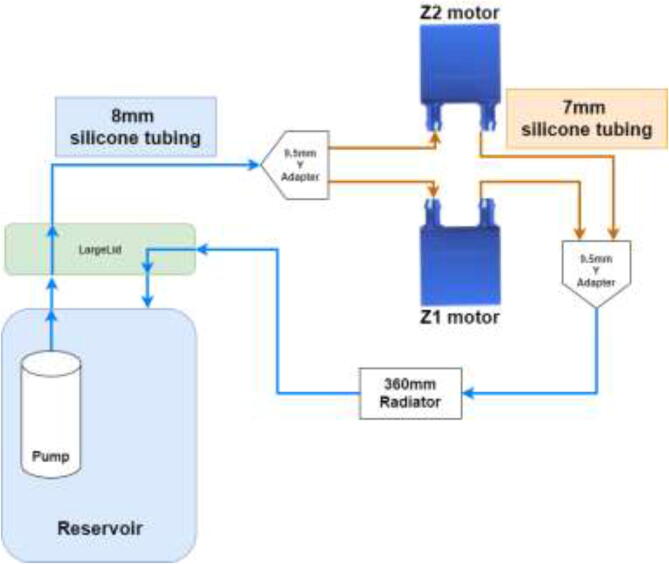
Z motor cooling loop.

Tubing and loop order for Z motor is shown in Fig. 109.

**Fig. 110:**
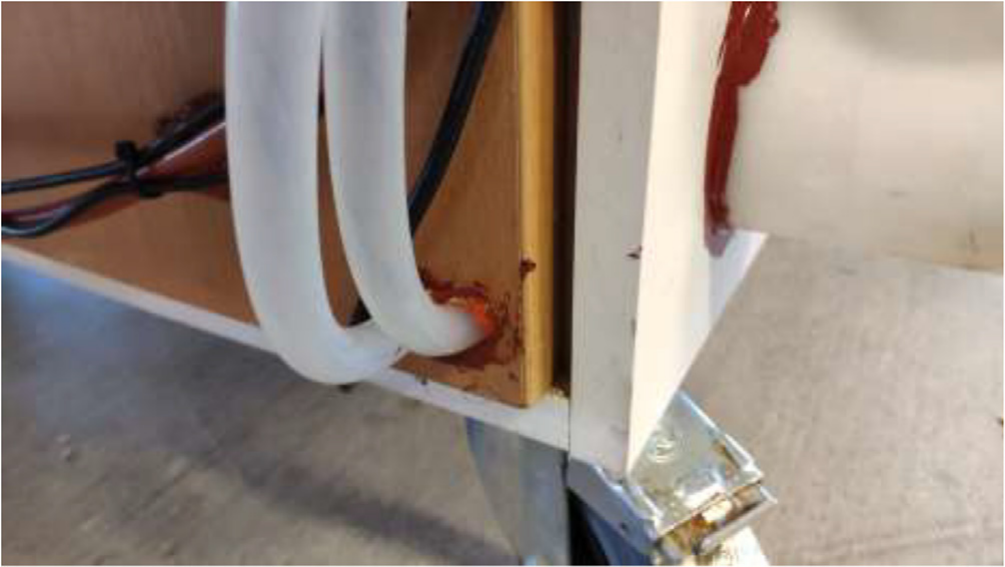
Z motor tubing.

Follow the methodology previously explained and connect according to the diagram. Add two extra holes in the bottom right of the backside, as seen in Fig. 110. Seal all holes for all axes using HTS and aluminum tape.

**Fig. 111:**
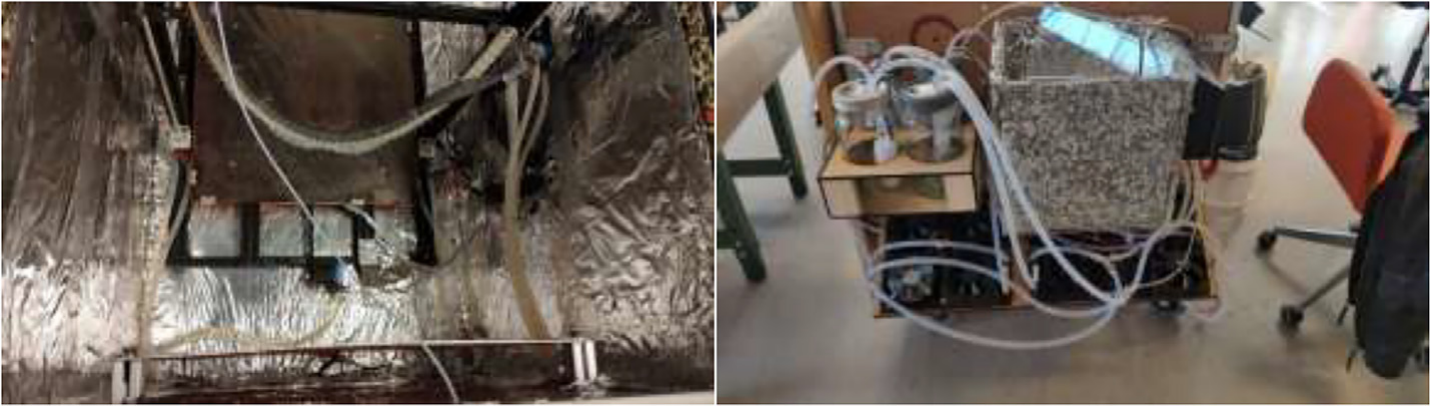
Tubing arrangement.

After installation of the liquid-cooling system, the tubing should resemble Fig. 111.

**Fig. 112:**
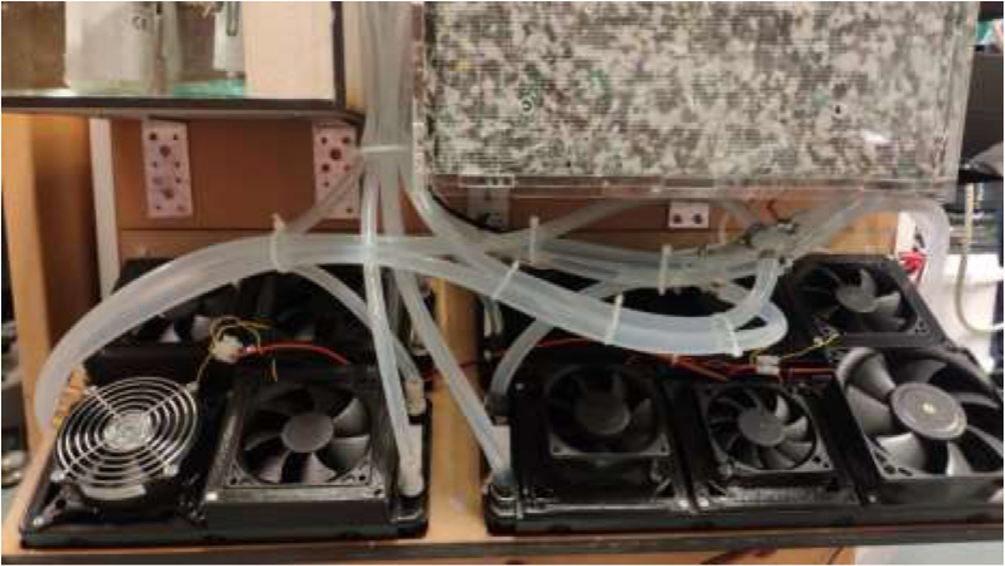
Arranged tubing.

Fill up all reservoirs with distilled liquid, preferably without coloring, to minimize the change of clogs. Tidy up tubes using zip ties (see Fig. 112).

### Drying chamber build

**Fig. 113:**
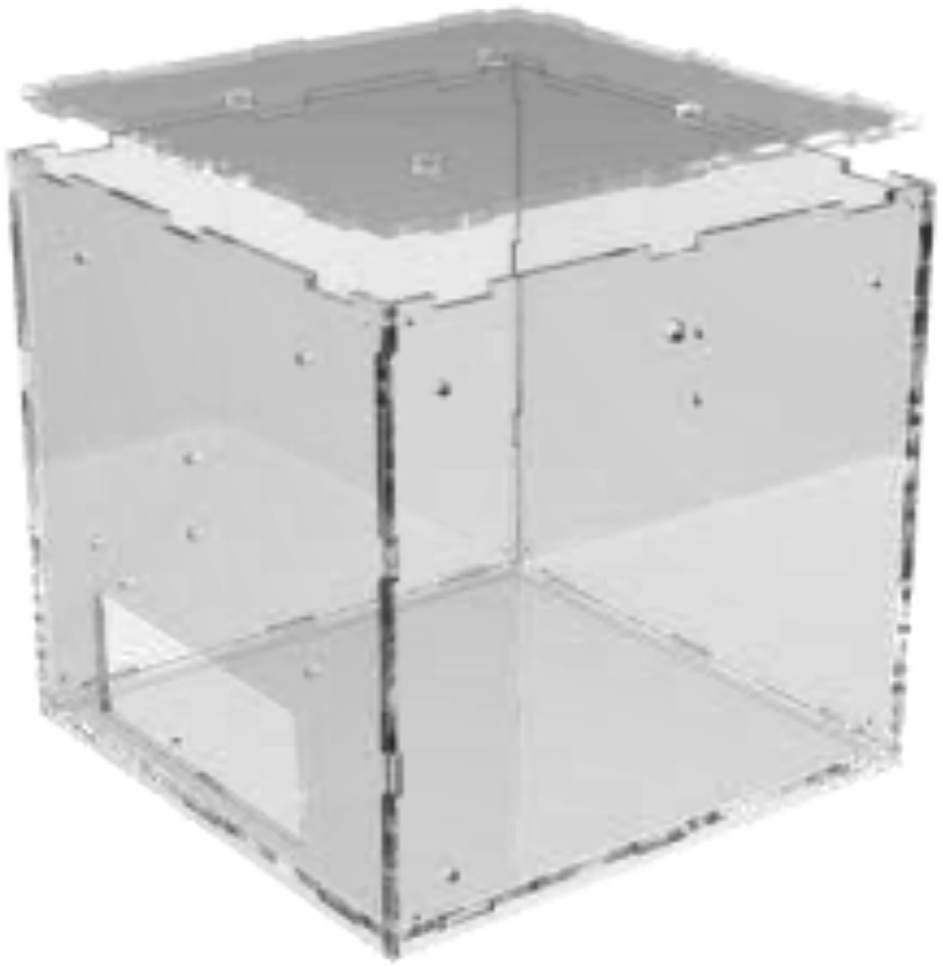
Laser-cut drying-chamber.

Print DC-ECase and DC-ECover. Print two pieces SpoolHolder in HF. The drying chamber are made using 6 mm acrylic and a laser cutter, but other materials capable of 100 °C could also be used. For laser cutting, DXF files are provided and assembled as in Fig. 113, marked with the required position. All bolts are mounted with a washer and a lock nut unless otherwise stated.

**Fig. 114:**
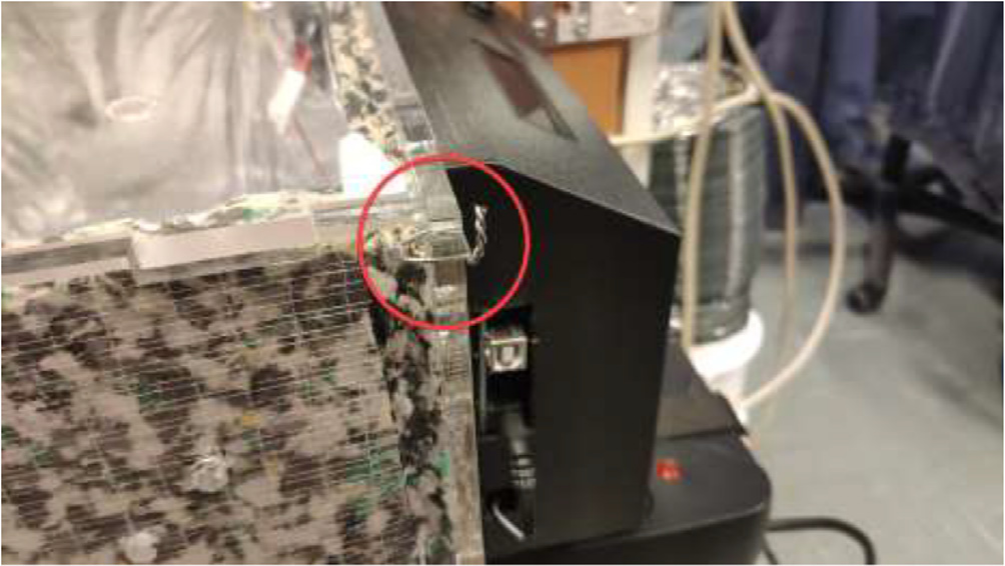
Steel wire to assemble drying-chamber.

Use steel wires to mount the corners as in Fig. 114 and seal all gaps using HTS.

**Fig. 115:**
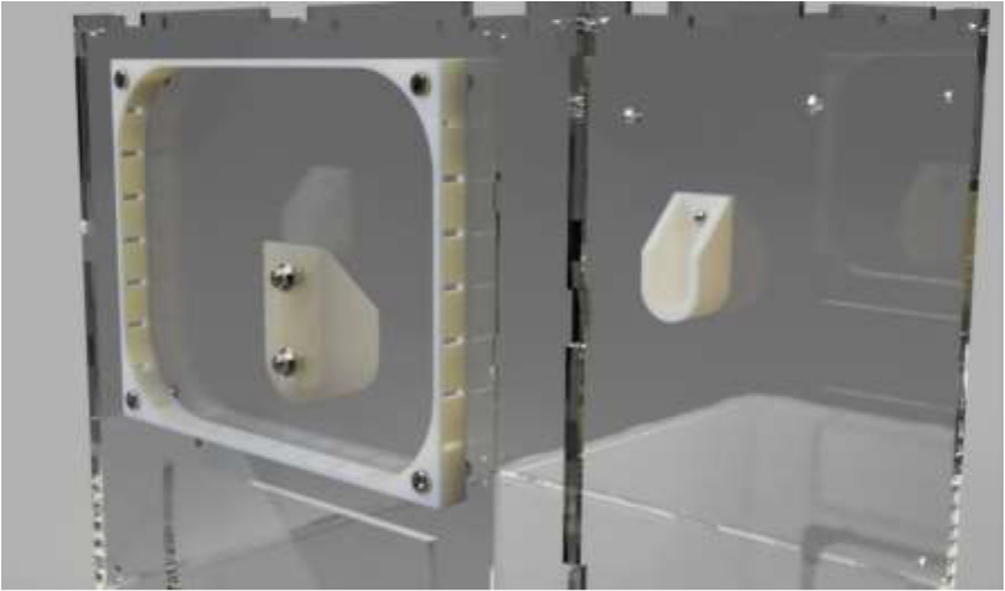
Spool holders and electronics spacer (used as passive cooling).

Mount spool holders using 4x M5x1 6 mm. Mount plastic spacer using 4x M5x25 mm bolts (see Fig. 115).

**Fig. 116:**
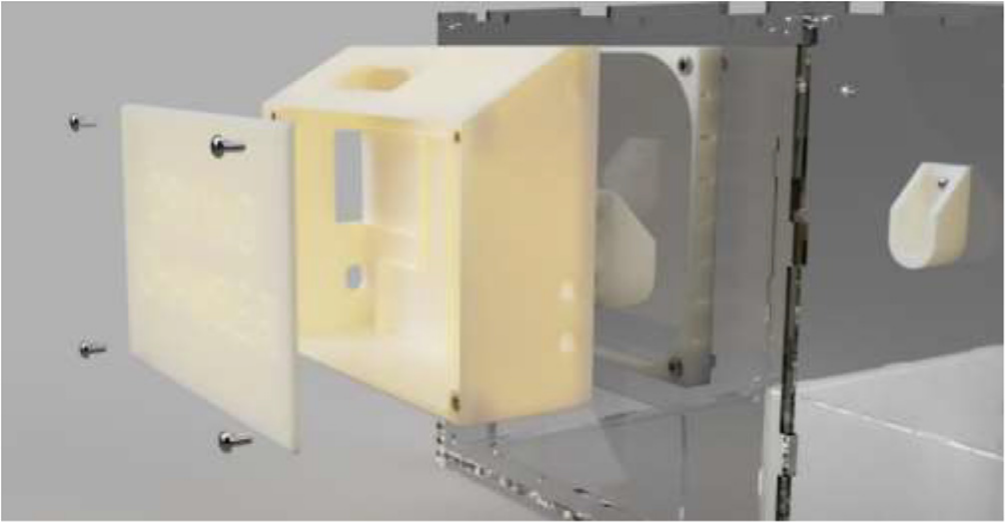
Electronics case mounting for drying-chamber.

Glue case to the spacer and insert brass inserts using a soldering iron. The cover is mounted using M5x10 mm bolts (see Fig. 116).

**Fig. 117:**
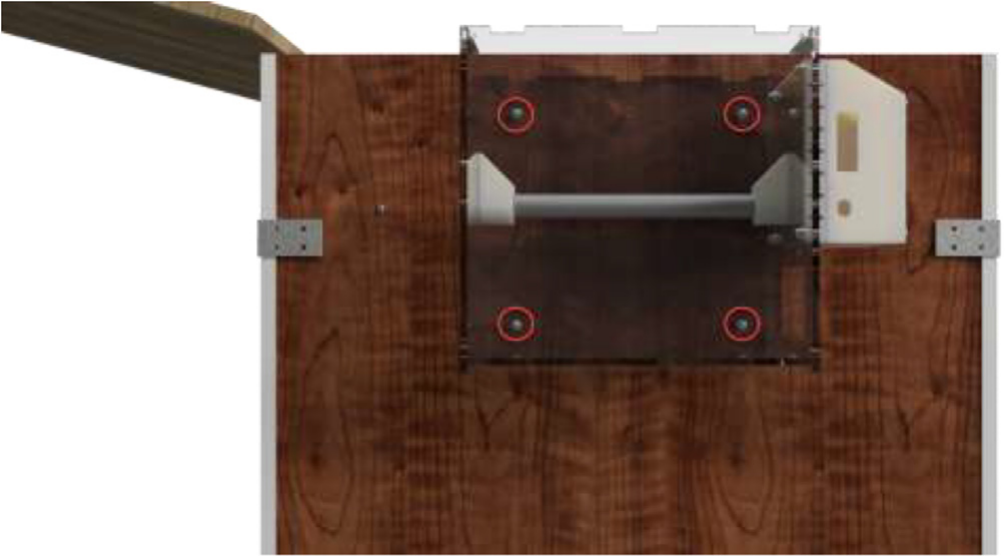
Drying chamber mounting.

The unit can optionally be used as a standalone unit or mounted to the back of the chamber using 4x M5x60 mm bolts (see Fig. 117).

**Fig. 118:**
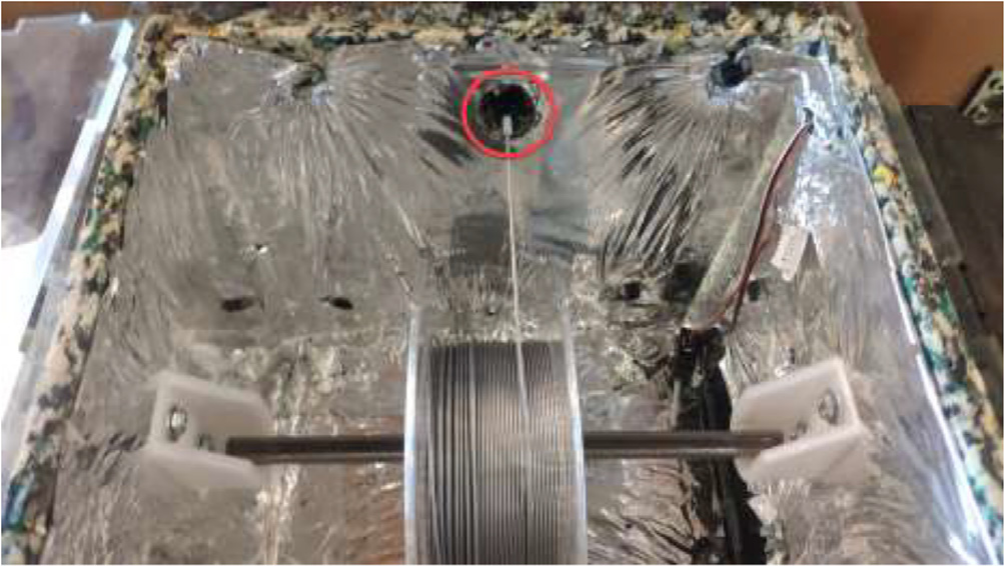
Bowden coupler.

Glue a Bowden coupler in the large hole in front of the case and pull Bowden tubing into the heated chamber as in Fig. 118.

**Fig. 119:**
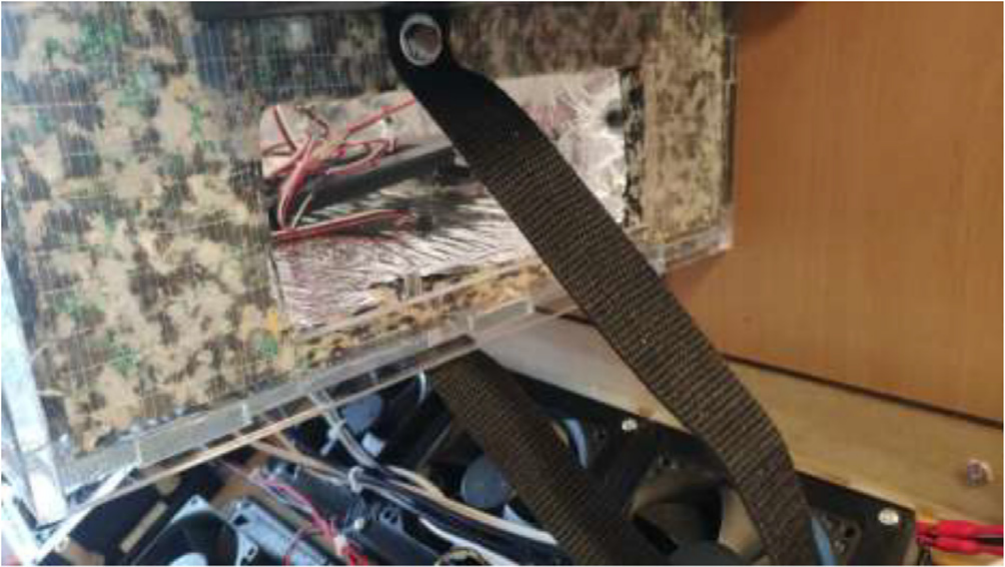
Mounting of the car heater.

Take a piece of steel or textile band and screw it into the drying chamber as in Fig. 119.

**Fig. 120:**
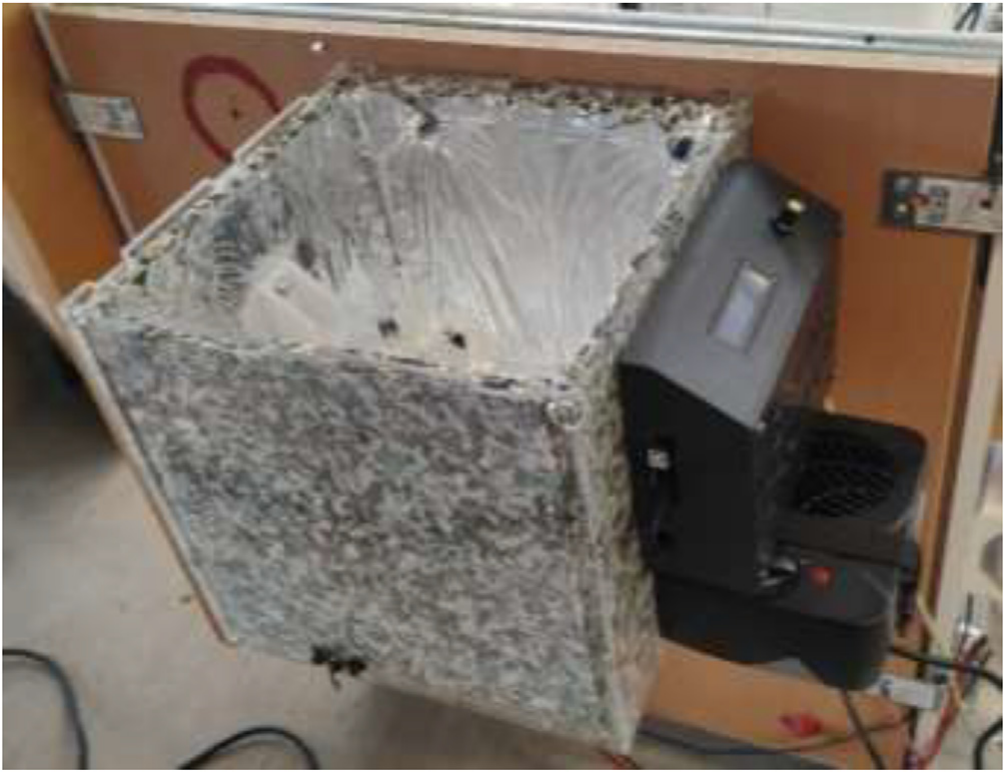
Drying chamber.

Proceed to insulate inside of drying chamber using 10 mm insulation and seal corners using aluminum tape. Ensure that the insulation does not cover the car heater inlet, the filament outlet, or the venting holes in the top cover. Cut a Ø24 mm diameter metal rod to 280 mm length as a filament holder. Mount car heater to the drying chamber as in Fig. 120. Use shims if necessary to ensure that the car heater is mounted correctly.

**Fig. 121:**
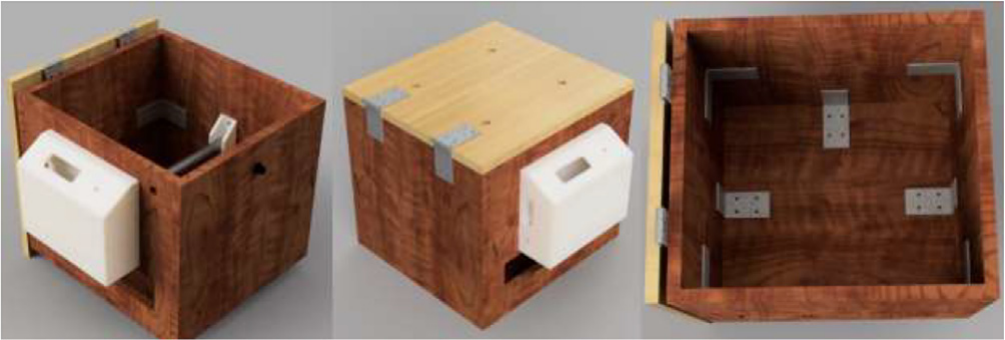
Wooden drying chamber assembly.

Alternatively, the drying chamber could be made using simple hand tools. STEP files and mechanical drawings are included. Cut and drill 16 mm wood panels according to provided files. Then, as shown in Fig. 121, assemble the box and use angle brackets and wood screws no longer than panel thickness.

**Fig. 122:**
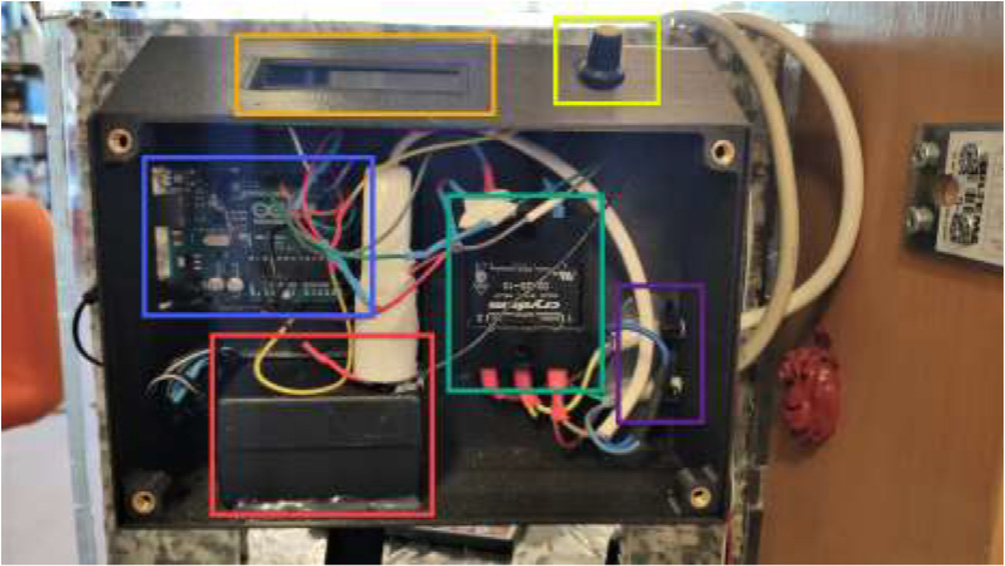
Drying chamber electronics placement. 1602 LCD (orange), 10 K potentiometer (yellow), Arduino Uno (blue), SSR (green), AC power in/out (purple) and 12v powerbrick + extension cord for Arduino (red). (For interpretation of the references to color in this figure legend, the reader is referred to the web version of this article.)

As seen in Fig. 122, place electronics inside the case use glue to make sure everything stays in place.

**Fig. 123:**
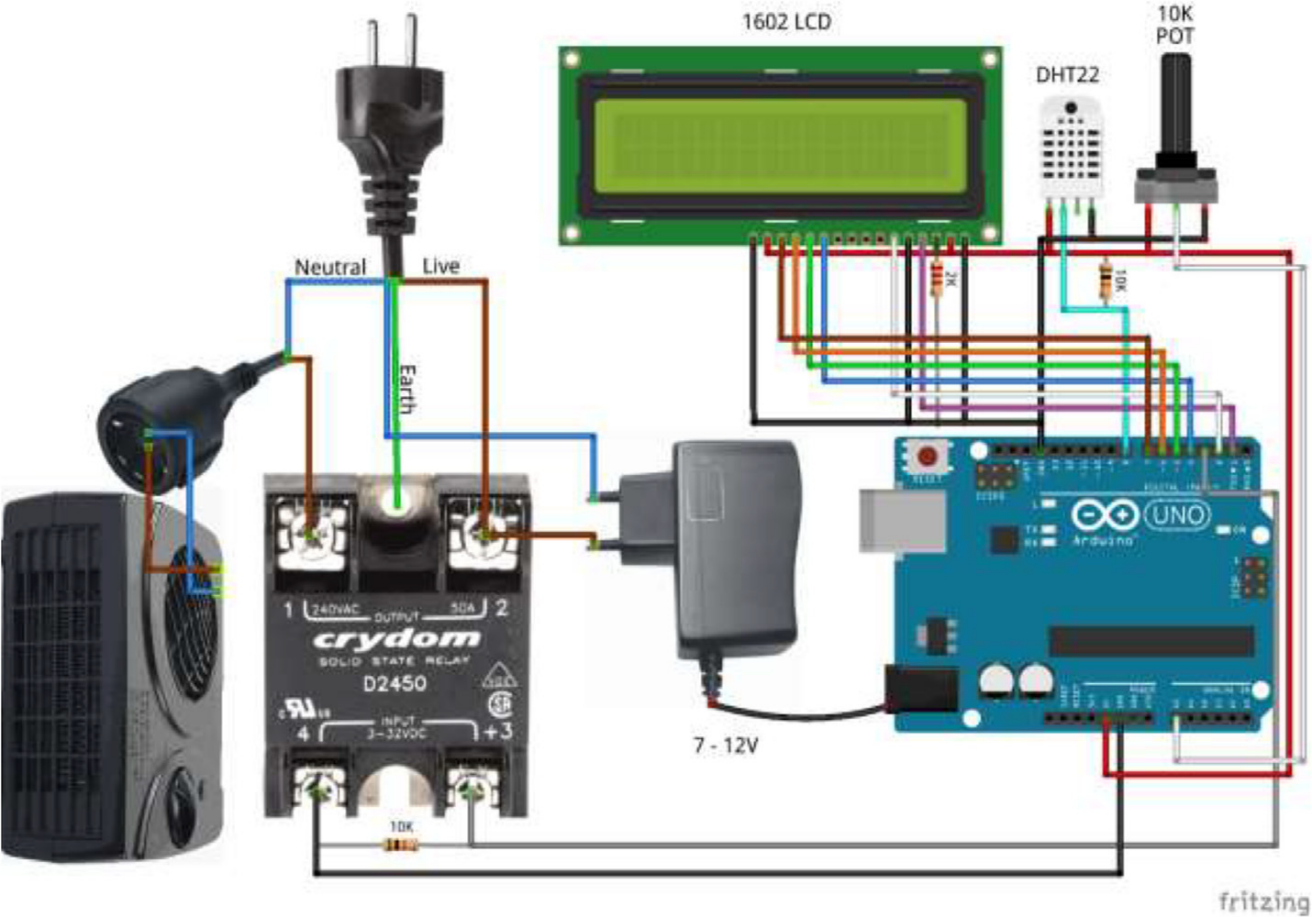
Drying chamber wiring diagram.

Wire electronics according to the diagram in Fig. 123. Place DHT22 sensor inside drying chamber and pull through the hole in the left side of the drying chamber to provide temperature and humidity measurements. Optionally, a filament sensor can be added – pull through the same hole as the DHT22 sensor.

**Fig. 124:**
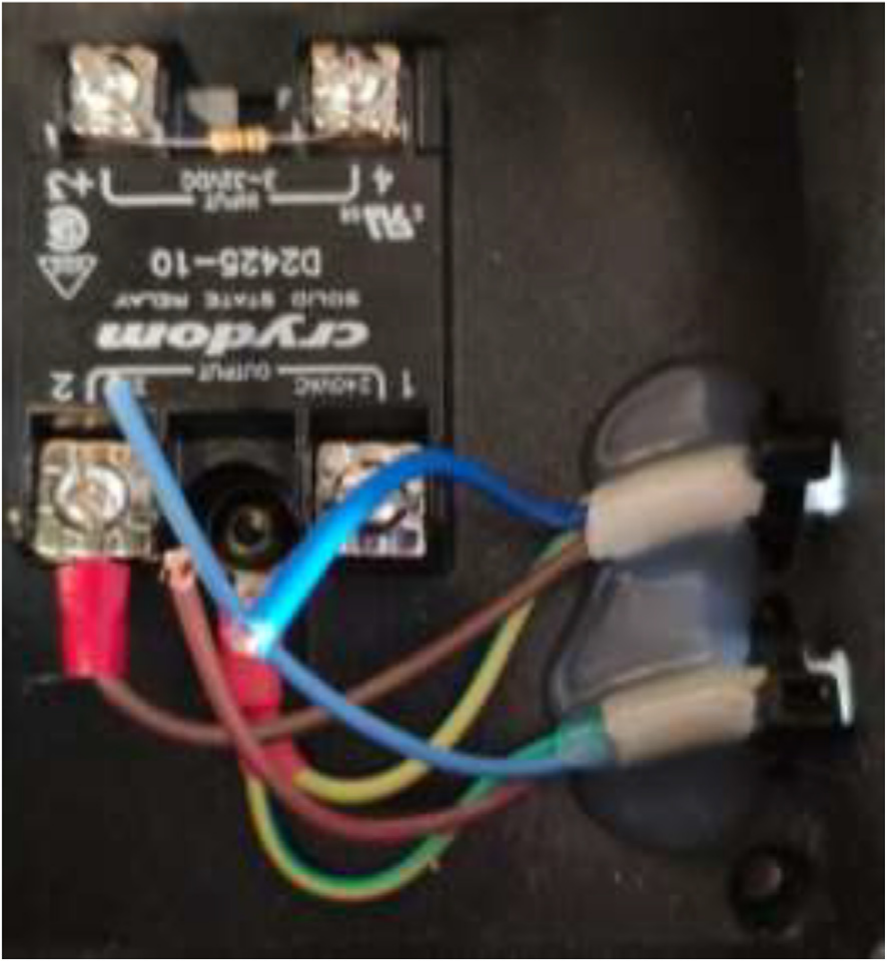
Drying chamber strain relief.

Make sure that the solid-state relay is adequately grounded. Next, create a strain relief on the power inlet and outlet using Zip ties and hot glue, as seen in Fig. 124. Also, add a printed SSR cover [27] or hot glue on terminals to avoid accidentally touching. By connecting the Arduino to a separate power supply instead of the ATX PSU, the drying chamber are able to run as a separate unit.

**Fig. 125:**
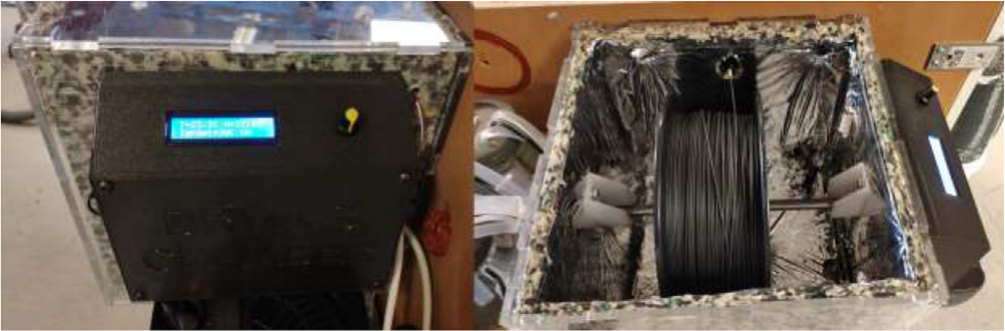
Finished drying chamber.

The finished drying chamber should look as shown in Fig. 125.

### Optionally, stepper temperature observation

A monitoring system could be installed if the builder chose to insert a thermistor into the stepper motors: print ThermistorMeasurementCase and ThermistorMeasurementCover. Then, mount the casing to the side of the heated chamber in the location shown in Fig. 72 and add brass inserts to the case.

**Fig. 126:**
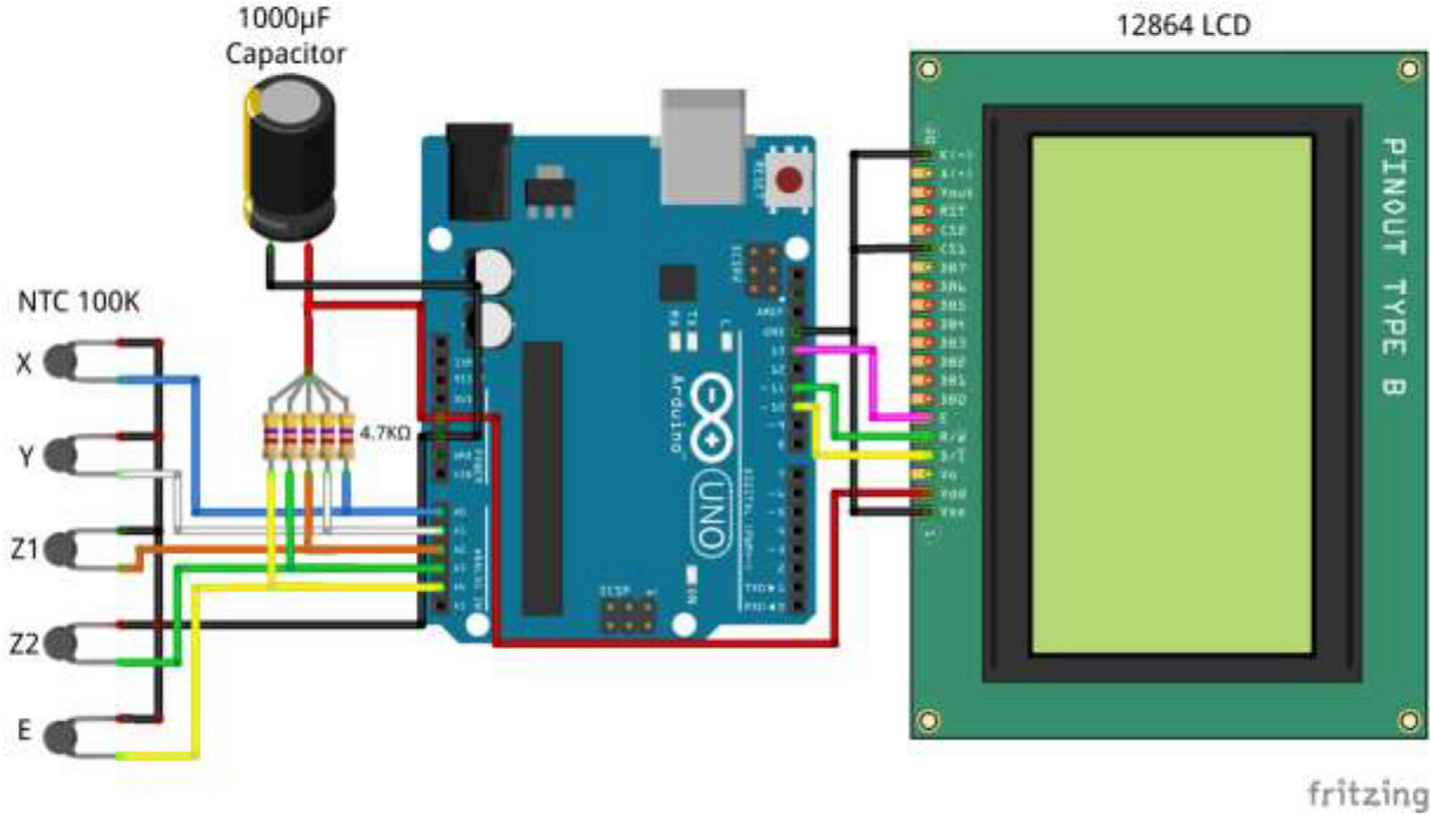
Thermistor readings, wiring diagram.

**Fig. 127:**
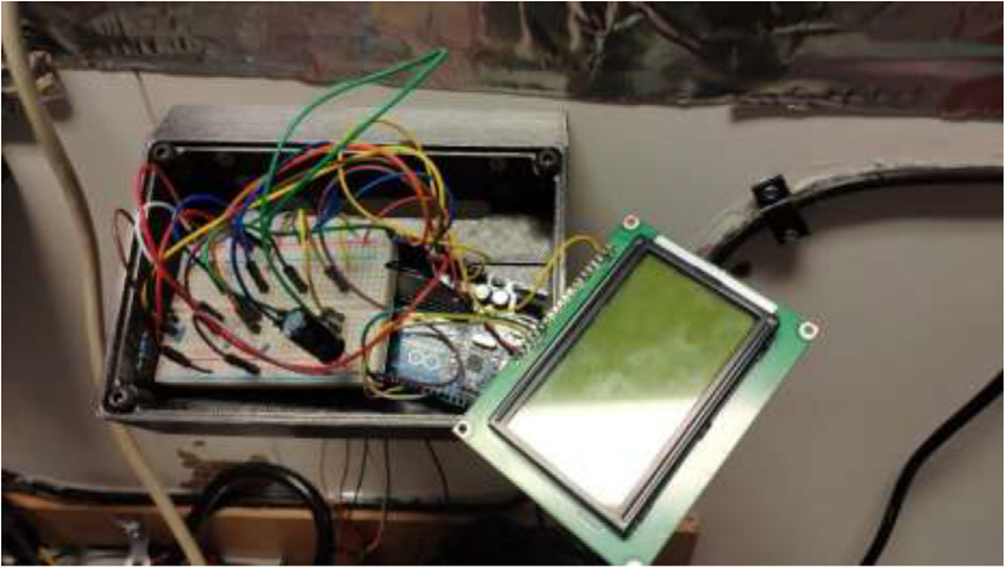
Stepper temperature electronics.

**Fig. 128:**
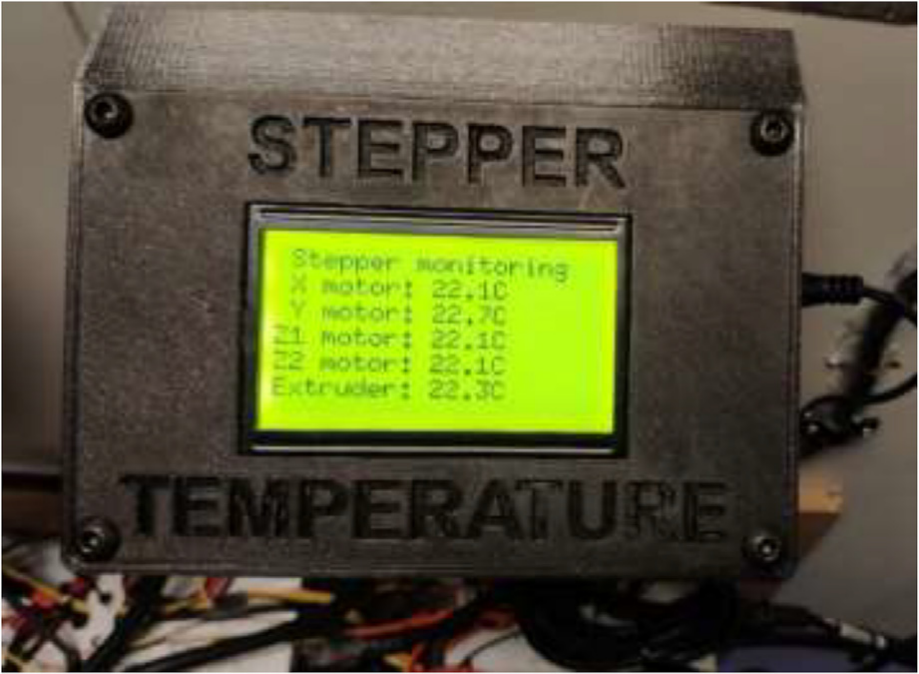
Stepper Temperature Monitoring.

Solder appropriate length wire and connectors to each thermistor and mark the wires. Using an Arduino Uno or Mega, five 4.7 K Ohm resistors, a 1000μ capacitor, and a 12,864 LCD wire according to Fig. 126. Insert electronics into the casing, as seen in Fig. 127. Use 4x M5x10 mm screws to close the cover. The finished build looks as in Fig. 128.

## Operation instructions

### Preflight checks

**Fig. 129:**
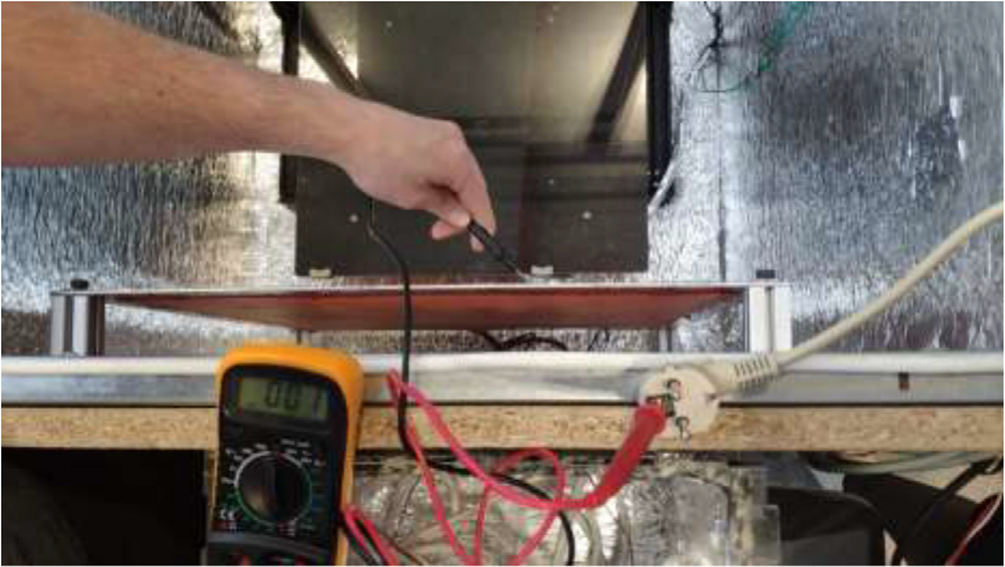
Checking proper grounding.

Before attempting to print high-temperature filaments, several checks should be performed. Start by checking that everything is properly grounded. Using continuity mode on a multimeter, connect one probe to the earth of the power cable and the other to the surface, which is tested in Fig. 129. The multimeter should make a sound and show ∼ 0 O, to show that it is successfully grounded.

Do this procedure for the chamber heater, the heated bed, Y-carriage, aluminum frame, outlets, and solid-state-relays. Check that all AC wires are connected correctly and adequately, as sparks and fire may occur if they are not. All live wires and SSRs should be covered to avoid accidentally touching. Check that the frame is mounted correctly and that the motion system and belts are tightened correctly. Lubricate the lead screws with sewing machine oil or similar. Finally, check that none of the original plastic parts are left in the chamber as they melt during higher chamber temperatures.

It is common for house outlets to provide a maximum of 3600 W (16A). The drying chamber uses up to 1200 W, the heated bed 1300 W, chamber heater 2500 W, and the electronics around 500–1000 W. At maximum power, the printer requires up to 6000 W; it is advisable to use separate outlets with separate fuses for the chamber heater and the rest of the electronics.

### Software setup

To run the 3D printer, the open-source software Klipper [28] will be used. The 3D printer will be controlled using an open-source network interface called Fluidd [29].

1. Start by following the FluiddPi homepage installation instruction to install FluiddPi on the Raspberry Pi 4 [30]. OctoPrint running on OctoPi is an alternative; however, Fluidd is more responsive and suitable for printers running Klipper. When the Fluidd UI can be accessed from the web browser, continue with the next step.
2. The next step is flashing Klipper to both mainboards. Follow the instructions on the Klipper homepage to build and flash both Arduino Mega/Ramps 1.4 combo and Creality V2.0 with Klipper [31]. While flashing, the board type for both mainboards should be ATMega2560.
3. After flashing Klipper, the configuration file needs to be adjusted. A baseline configuration file is uploaded and can be used to create the preferred configuration file. A set of extra additional examples is also included to ease the setup of the final configuration. Reference and explanations of the options are in the Klipper Docs [32]. The configuration must, in most cases, be adjusted to the system used.

### Using Fluidd and Klipper

**Fig. 130:**
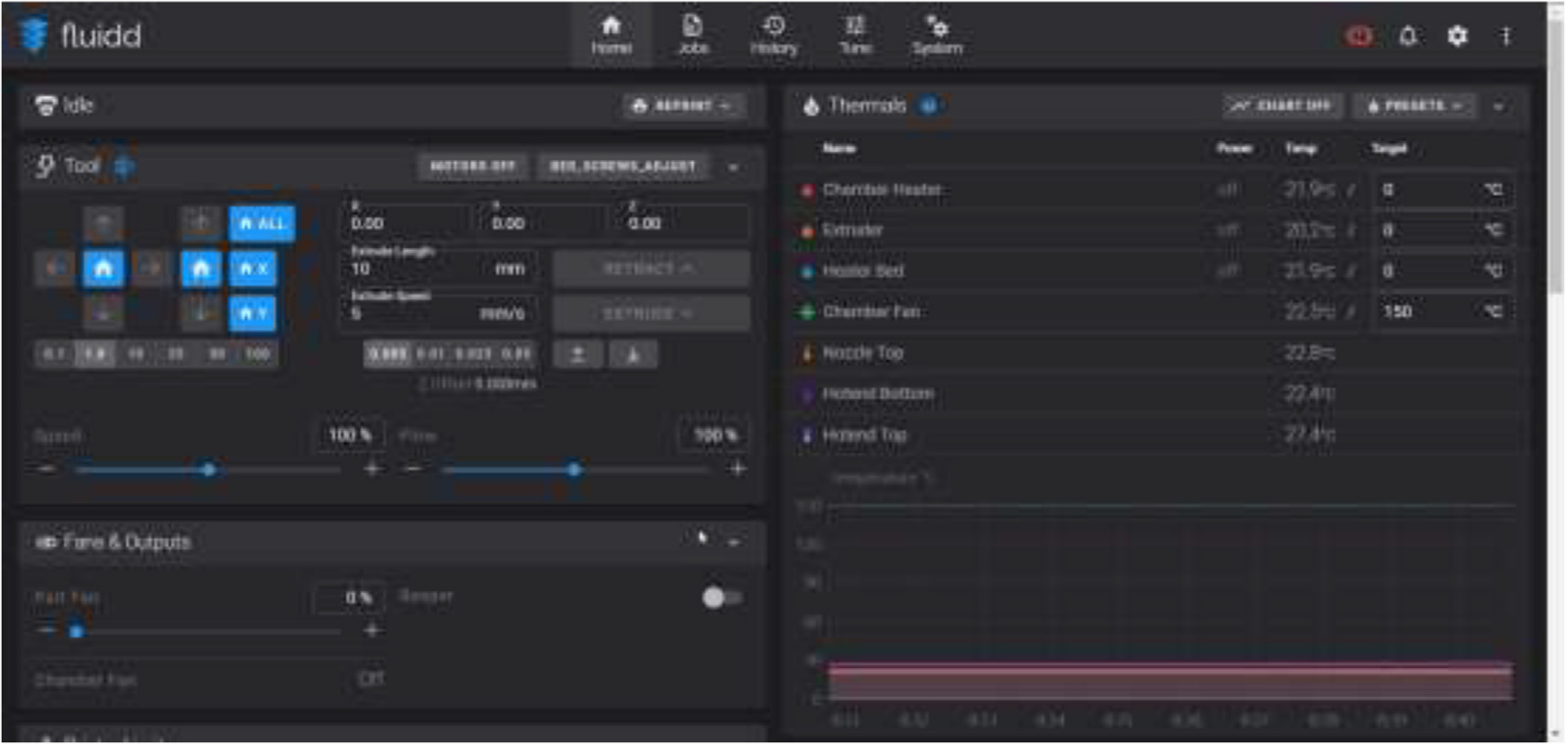
Fluidd interface.

In the Fluidd interface, as shown in Fig. 130, temperatures can be adjusted and monitored. When adding extra heaters or thermistors in the configuration file, they will automatically appear in this interface. Make sure that all temperature sensors provide accurate measurements with minimal fluctuation. “Chamber Fan” adjusts the temperature at which the fan will start running.

**Fig. 131:**
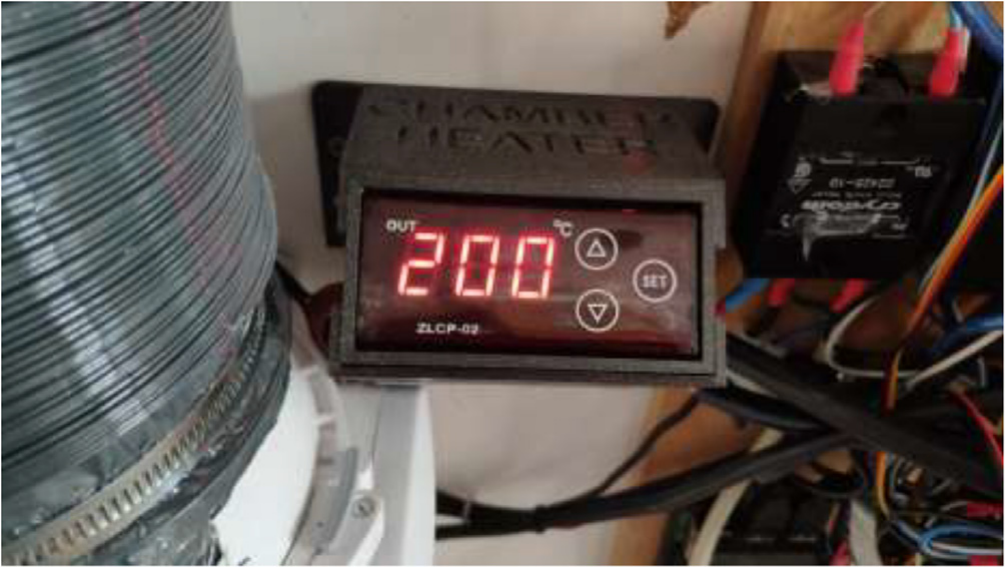
Integrated PID controller.

Under “Chamber Heater,” the target temperature of the chamber is inserted. The chamber heater will turn on if the temperature in the chamber is below target and turn off once the target is reached using the Bang-Bang control method. The maximum temperature of the silicone heater is adjusted on the PID controller either outside (Fig. 131) or under “Thermal” as an additional heater if one uses the external PID controller. The heater may be adjusted up to 220 °C. Lower temperatures will increase the heater and the system’s life span. However, increase the heat-up time of the system. Depending on the configuration and target temperature, the chamber may need to pre-heat for several minutes/hours before starting the print.

**Fig. 132:**
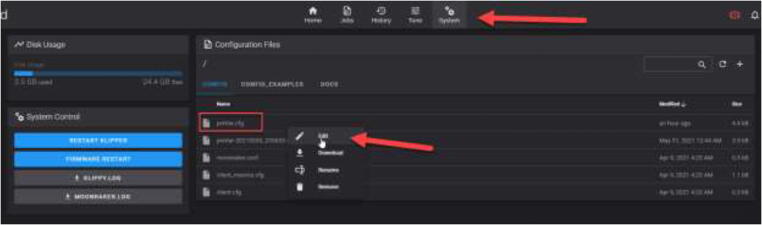
Editing configuration file.

In the tab “System,” the configuration file can be viewed and edited according to the user’s needs (see Fig. 132). This file must be edited to the configuration being used.

### Setting up and using Liquid-Cooling system and drying chamber

If temperature sensors are installed in reservoirs, flash “LiquidTemp.ino” to Arduino Uno Arduino IDE [33] to enable monitoring. All pumps and fans can be turned on/off by a switch, and each pump has its separate speed control. The speed should be adjusted to provide enough cooling. However, lower is better as higher flow could result in a higher chance of leakage. If thermistors are installed in stepper motors, flash “StepperTemp.ino” to Arduino to enable LCD monitoring of stepper. These measurements could be used to optimize the flow of liquid.

**Fig. 133:**
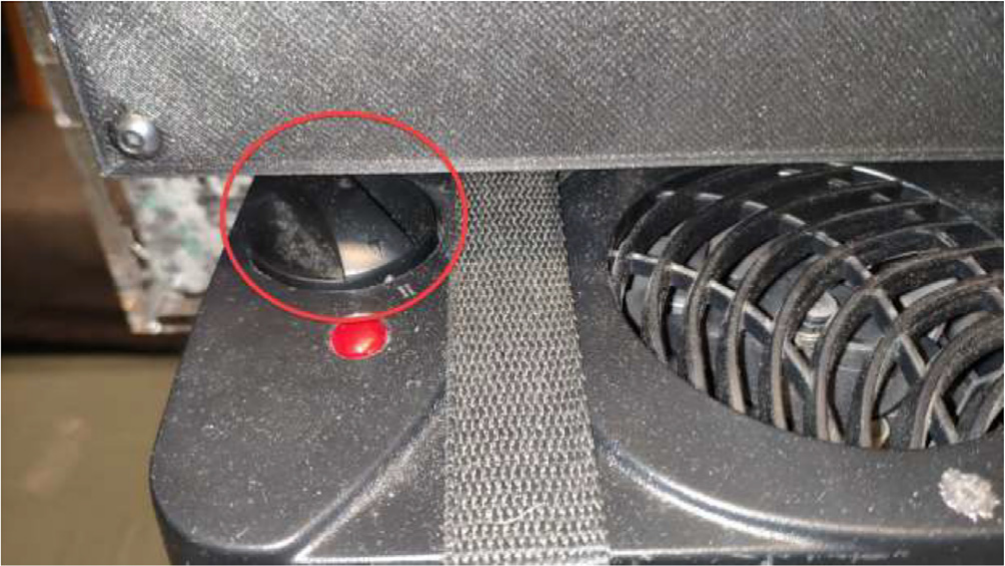
Power setting for drying chamber.

For the drying chamber, start by compiling and uploading the Arduino code “DryingChamber.ino” to the Arduino Uno. The drying chamber could either be used to dry moist filament or to keep filament dry during printing. Filaments such as nylon are very prone to absorbing moisture and should have the drying on at all times. The temperature can be set up to 90 °C. However, it should be as low as possible to avoid overheating. Set the output power of the car heater to either level I or level II (Fig. 133).

**Fig. 134:**
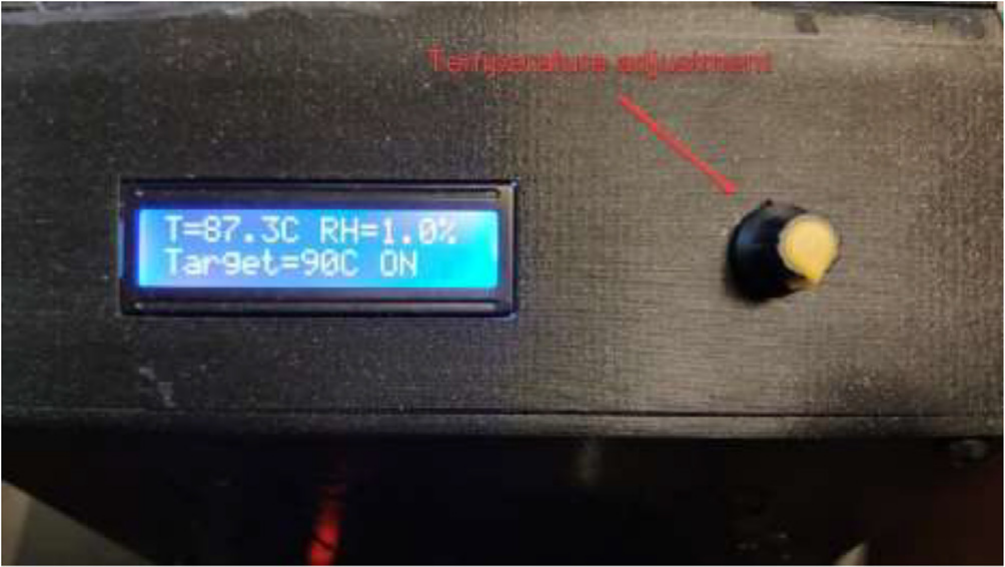
Drying Chamber control panel.

Level 1 will work well for filaments that require low temperatures, at 75 °C to dry or keep humidity out of the box during printing. However, this setting will not allow the drying chamber to achieve the maximum temperature. Level 2, at 90 °C, is excellent for drying filaments that require higher temperatures. The temperature is set using the potentiometer, as shown in Fig. 134. The LCD will show the current temperature, relative humidity, and target temperature and indicate if the heater is currently running.

### Calibration

After software setup, it is essential to validate and calibrate the system to ensure safe operation and high quality prints.

1. Validation and PID tuning

First, verify that all components are working correctly by following the Klipper Config Checks document [34]. Follow the instructions on the same page to PID tune hotend heater, bed heater, and chamber heater. If the chamber heater has an included PID controller, this step is unnecessary for this heater as the chamber temperature is controlled using bang-bang. After the tuning, heat the nozzle to a working temperature and tighten it to ensure no leaks.

2. Bed leveling

The system does not include an auto-level sensor. Start by adjusting the Z limit switch to a position where the axis can safely home. Adjust the “Homing Override” in the Klipper config file if necessary. Proceed by leveling the bed manually by following the guide on Klipper GitHub [35]. If the bed is warped or uneven and produces an uneven first layer, one could use manual bed mesh leveling as described in the GitHub docs [36]. It is recommended to heat the chamber and nozzle to operating temperature before bed leveling as temperature differences influence the system.

3. Optionally, fine-tuning

Klipper implements several functions, which enables more dimensionally accurate parts with a good surface finish. To reduce ringing on parts, the resonance can be compensated using the guide on Klipper homepage [37]. The resonances can also be measured using an accelerometer, as explained under “measuring resonances” [38]. Calibrate pressure advance using Klipper’s guide, which reduces blobbing in corners and reduces oozing from the hotend [39]. To ensure that all stepper motors output the correct number of steps per millimeter, the rotation distance can be calibrated for all axes and the extruder. Follow the Klipper Docs “Rotation Distance” [40] to calibrate this parameter. It is crucial to create dimensionally correct parts with the correct amount of filament extruded.

### Printing high-temperature filaments

High-temperature filaments may clog the system if residues of filament are left inside the nozzle when the nozzle is cold. Therefore, it is recommended to leave the heater after printing, then decrease the temperature to 300C and flush the extruder with another filament such as PolyCarbonate. After the filament is fully extruded, the nozzle can be cooled safely. When printing high-temperature materials, additional adhesive should increase adhesion and decrease warping [41]. Printed parts must be removed while the bed is hot to avoid shattering of glass. Failed prints in high-performance materials may lead to irreversible damage on the extruder and bed as the filaments fuse exceptionally well and are very difficult to remove.

High-temperature filaments will often absorb large amounts of moisture from the air. To avoid filament moisture absorption, it is recommended to keep the filament in a dry box with silica gel during storage and have the drying chamber set at an elevated temperature during printing. The drying chamber can also be used to dry moist filament by increasing the temperature further. It is important to keep the printer in a well-ventilated area. The exhaust fan may also extract fumes through a filter or outside through a window. Some parts, especially the belt and limit switch, are run above their recommended rated temperature. Over time the belt may stretch, so it is essential to tighten regularly. It may also need replacing more often than usual. The limit switch should be checked before each print and replaced if necessary.

## Validation and characterization

The presented printer design uses Superslicer, an open-source version of PrusaSlicer. The calibrated slicer profile is available in the repository.

### Chamber and stepper temperature

**Fig. 135:**
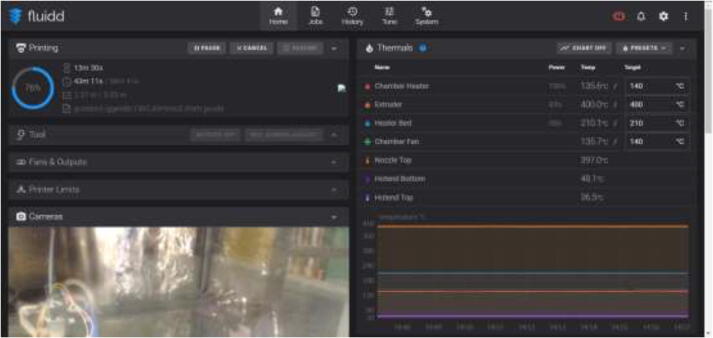
Maximum obtainable chamber temperature.

The proposed 3D-Printer was tested, and the maximum obtainable chamber temperature was 135.6 °C, as seen in Fig. 135.

**Fig. 136:**
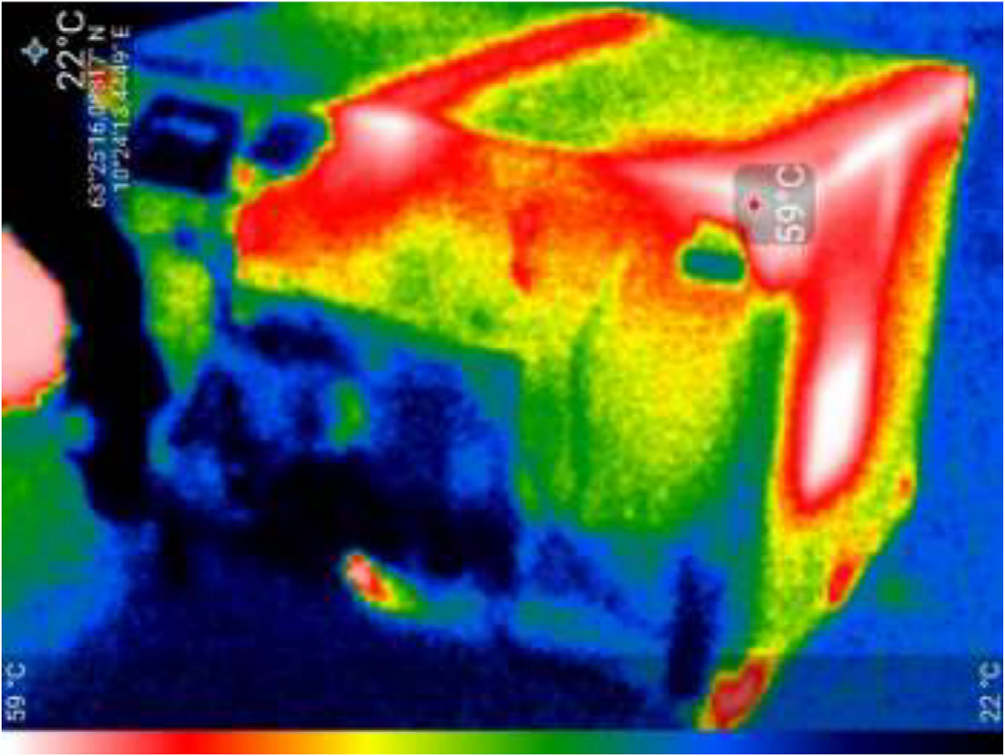
Thermal Image of heated chamber during operation.

Thermal image of the heated chamber is shown in Fig. 136. The chamber is sufficiently insulated, however, there are some heat losses from the window and the rails along the top.

**Fig. 137:**
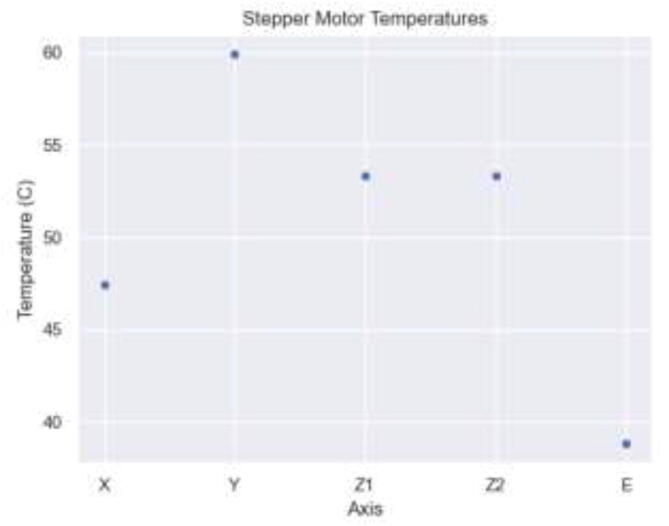
Stepper motor temperature during maximum chamber temperature.

While printing at 70 mm/s, 200 °C bed, 400 °C nozzle, and 135 °C chamber for one hour, the stepper temperatures were measured to be as seen in Fig. 137, indicating sufficient cooling. While the Y stepper motor had the highest temperature of 60 °C, all stepper motors ran within the recommended operating temperature below 70–80 °C.

### Objects printed in CF-PEEK

To evaluate the modified printer’s capability to manufacture in carbon fiber reinforced PEEK, a 3DBenchy and spiral vase were printed using CarbonX, PEEK-CF from 3DXTECH. The printer settings are presented in Table 1. An adhesive Dimafix Pen 90 ml thermal glue was applied to the glass bed before initiating the bed pre-heating process.

**Table 1:** Printer settings used for the 3DBenchy and spiral vase manufactured in PEEK-CF.

Material	CarbonX PEEK-CF
Chamber type	Insulated
Filament drying	Continuous drying
Nozzle	0.6 mm hardened steel
Extruder temperature, °C	400
Bed temperature, °C	140
Enclosure temperature, °C	135
Layer height, mm	0.15
Line With, mm	0.65
Perimeters	1
Speed mm/s	70
First layer speed, mm/s	10
Infill, %	20

**Fig. 138:**
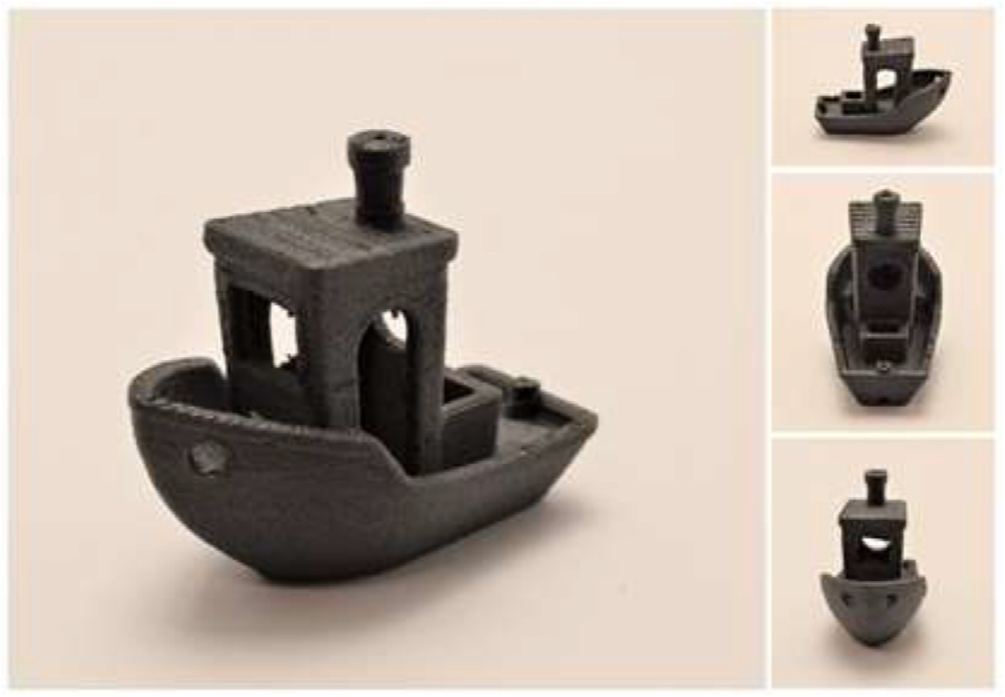
3DBenchy printed to showcase the geometrical details of printed CF-PEEK.

Fig. 138 shows a 3D-printed 3DBenchy in CF-PEEK printed with a chamber temperature of 90 °C, bed 150 °C, and 400 °C nozzle. It was printed at 5 mm/s with 20% infill and a 1:1 scale to the original model. The part shows successfully printed details and overhangs.

**Fig. 139:**
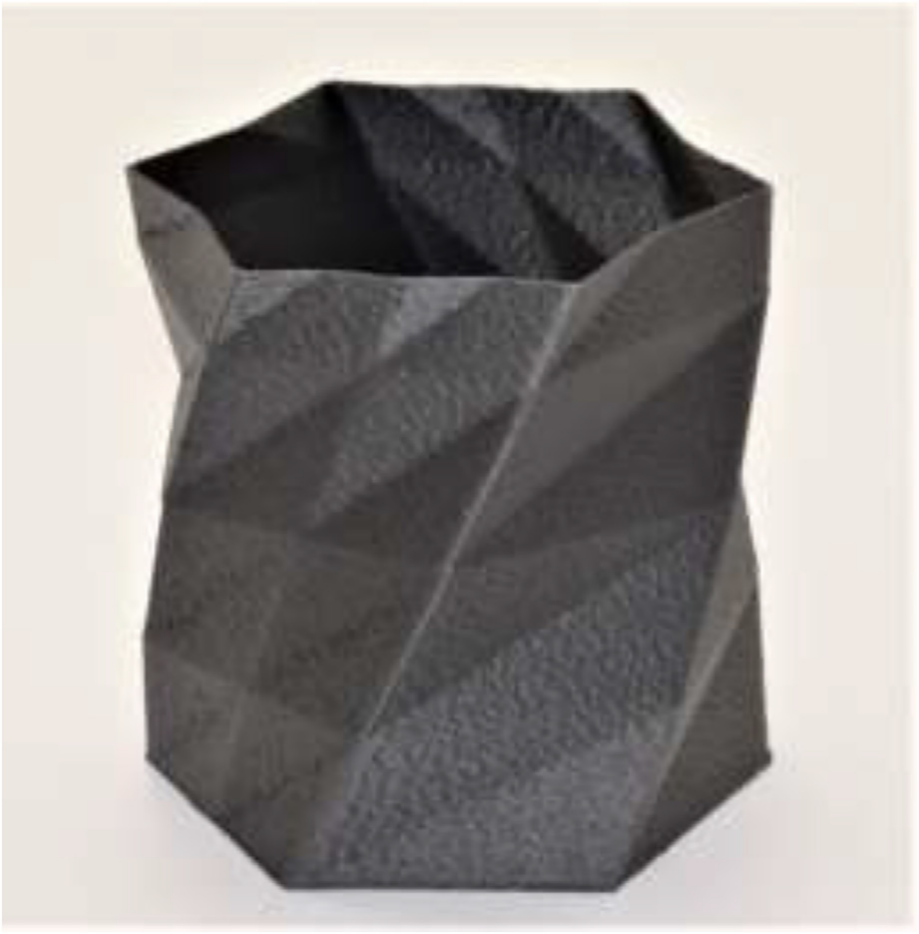
Vase printed to demonstrate the ability to print larger parts in Z-direction. The height of the item was 100 mm.

Fig. 139 shows a printed vase in CF-PEEK. The vase shows no sign of delamination, and the quality at the top of the model is comparable to the bottom, which indicates an adequately uniform chamber temperature.

### Validation print of carbon fiber nylon

The printer was tested using PolyMide PA6-CF from Polymaker [42]. According to ISO 527, 5 samples were printed on the presented printer and an Original Prusa i3 MK3S with a hardened nozzle inside a non-heated chamber. Dimafix Pen 90 ml thermal glue was used as adhesive and the main settings for both printers are presented in Table 2.

**Table 2:** Printer settings used for ISO527 test samples.

Characteristic	Modified printer	Prusa MK3S
Chamber type	Insulated	Non-insulated plexiglass
Filament drying	Continuous drying	Pre-dried
Nozzle	0.6 mm hardened steel	0.6 mm hardened steel
Extruder temperature, °C	300	292
Bed temperature, °C	120	120
Enclosure temperature, °C	75	28
Layer height, mm	0.3	0.3
Line With, mm	0.65	0.65
Perimeters	1	1
Printing speed XZ specimens, mm/s	8	5
Printing speed XY specimens, mm/s	70	70
First layer speed, mm/s	10	10
Infill, %	100	100

When manufacturing the samples using the Prusa Mk3S and the modified, both printers were thoroughly calibrated with identical primary settings. As there was a difference in printer capabilities, the main deviations are arguably acceleration, nozzle effect, temperature, and drying. Due to the capabilities of the Prusa Mk3, the maximum nozzle temperature was limited to 292 °C, 8 °C lower than the modified printer. When printing on the Prusa, the material was pre-dried for 6 h at 100 °C for 6 h according to manufacturer’s recommendations. The filament was then put into air-sealed bags with silica with a Teflon tube running to the printer. While printing at the modified printer the filament where first pre-dried, then continuously dried until finishing the samples.

**Fig. 140:**
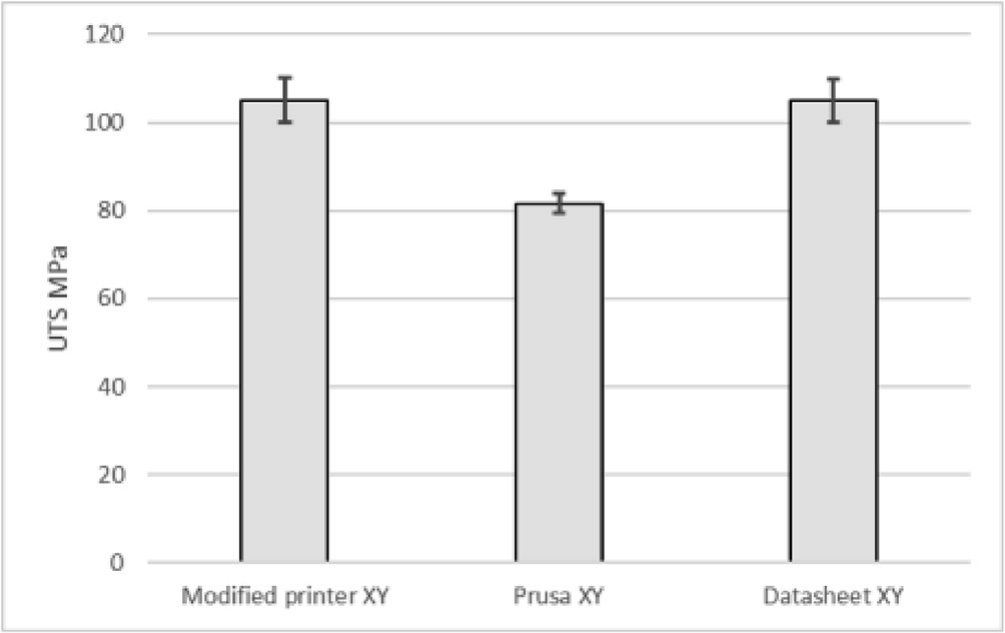
Tensile test of the XY direction with samples lying flat on the bed according to ISO527.

The presented printer managed to print PA6-CF according to the datasheet in the XY direction with the samples lying flat on the printer bed and outperforming the Original Prusa i3 MK3S by 22% in ultimate tensile strength (see Fig. 140).

**Fig. 141:**
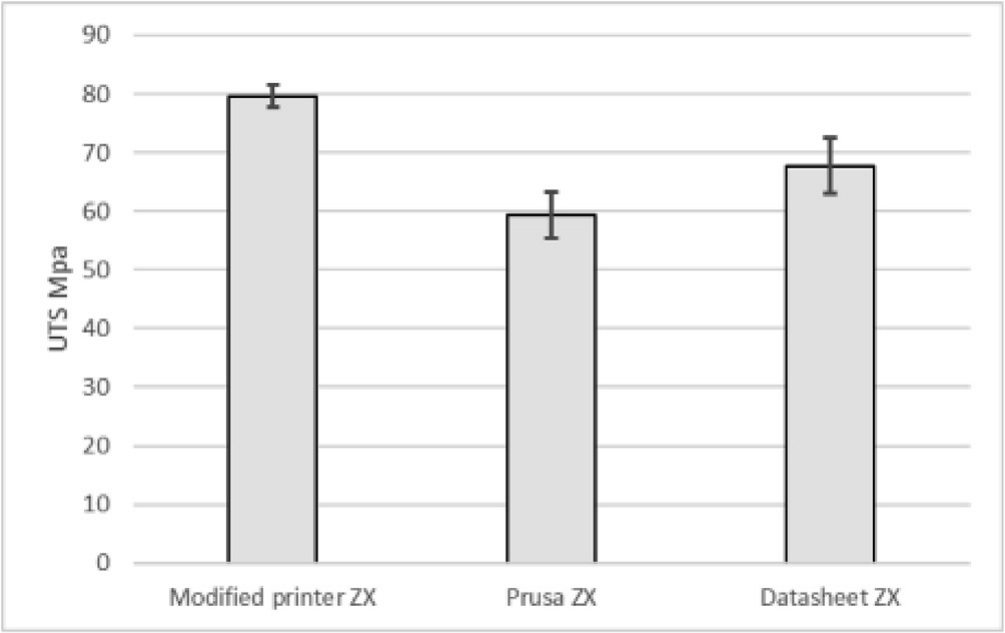
Tensile test in the XZ direction with standing samples on the printer bed for testing layer adhesion according to ISO527.

When printing samples in the ZX direction (Fig. 141) to test layer adhesion, the presented printer outperformed the properties provided by the material manufacturer [42] by 15% and the Original Prusa i3 MK3S by 25%.

## Declaration of Competing Interest

The authors declare that they have no known competing financial interests or personal relationships that could have appeared to influence the work reported in this paper.

## References

[1] Wohlers T., Campbell I., Diegel O., Huff R., Kowen J. (2020).

[2] S. S. Crump, “Apparatus and method for creating three-dimensional objects,” US5121329A, Jun. 09, 1992 Accessed: Dec. 17, 2021. [Online]. Available: https://patents.google.com/patent/US5121329/en?oq=scott+crump

[3] Jones R., Haufe P., Sells E., Iravani P., Olliver V., Palmer C., Bowyer A. (2011). RepRap–the replicating rapid prototyper. Robotica.

[4] Tan L.J., Zhu W., Zhou K. (2020). Recent progress on polymer materials for additive manufacturing. Adv. Funct. Mater..

[5] Das A., Chatham C.A., Fallon J.J., Zawaski C.E., Gilmer E.L., Williams C.B., Bortner M.J. (2020). Current understanding and challenges in high temperature additive manufacturing of engineering thermoplastic polymers. Addit. Manuf..

[6] Zawaski C., Williams C. (2020). Design of a low-cost, high-temperature inverted build environment to enable desktop-scale additive manufacturing of performance polymers. Addit. Manuf..

[7] Choi Y.-H., Kim C.-M., Jeong H.-S., Youn J.-H. (2016). Influence of Bed Temperature on Heat Shrinkage Shape Error in FDM Additive Manufacturing of the ABS-Engineering Plastic. WJET.

[8] Yang C., Tian X., Li D., Cao Y., Zhao F., Shi C. (Oct. 2017). Influence of thermal processing conditions in 3D printing on the crystallinity and mechanical properties of PEEK material. J. Mater. Process. Technol..

[9] Yan Y., Zhang R., Hong G., Yuan X. (Apr. 2000). Research on the bonding of material paths in melted extrusion modeling. Mater. Des..

[10] Zaldivar R.J., Mclouth T.D., Ferrelli G.L., Patel D.N., Hopkins A.R., Witkin D. (Dec. 2018). Effect of initial filament moisture content on the microstructure and mechanical performance of ULTEM® 9085 3D printed parts. Addit. Manuf..

[11] Woern A.L., McCaslin J.R., Pringle A.M., Pearce J.M. (2018). RepRapable Recyclebot: Open source 3-D printable extruder for converting plastic to 3-D printing filament. HardwareX.

[12] “Funmat HT Enhanced,” *Vision Miner*. https://visionminer.com/products/funmat-ht-enhanced (accessed May 04, 2021).

[13] “AON-M2,” *Vision Miner*. https://visionminer.com/products/aon-m2 (accessed May 04, 2021).

[14] Gardner J., Stelter C., Yashin E., Siochi E. (2016). High Temperature Thermoplastic Additive Manufacturing Using Low-Cost. Open-Source Hardware..

[15] Skrzypczak N.G., Tanikella N.G., Pearce J.M. (2020). Open source high-temperature RepRap for 3-D printing heat-sterilizable PPE and other applications. HardwareX.

[16] “https://www.ikea.com/no/no/p/metod-benkeskapstamme-hvit-10205633/,” *IKEA*. https://www.ikea.com/no/no/p/metod-benkeskapstamme-hvit-10205633/ (accessed May 21, 2021).

[17] “CR10-Plus.” https://images-na.ssl-images-amazon.com/images/I/7128lnpsHKL.jpg (accessed Jun. 01, 2021).

[18] “3D-printer-CR-10S_005_1920x1080.jpg - HomoFaciens.” https://www.homofaciens.de/bilder/technik/3D-printer-CR-10S_005.htm (accessed Jun. 01, 2021).

[19] “12V 30A Power Supply, Mean Well 350W LED Driver.” https://www.bestledstrip.com/12v-30-amp-power-supply-350watt-lrs-350-12/ (accessed Jun. 03, 2021).

[20] “Eu 220v Ac Lead Cord 3*0.75mm Wire Open End Stripped Diy Mains Psu Cable Extension European 3prong Cee7/7 Power Plug - Buy Psu Cable Extension European 3prong Cee7/7 Power Plug,Replacement European 10a/220v Schuko Power Cord 2m Open End Stripped Wires Cee7/7 Ac Extension Lead For Dishwasher Freezer,1m 2m 10ft 1.5m Vacuum Cleaner Eu Power Cable 3*0.75mm Rewired Cable Schuko Cee 7/7 Stripped End Power Extension Lead Product on Alibaba.com.” https://www.alibaba.com/product-detail/EU-220V-AC-Lead-Cord-3_1600137351985.html (accessed Jun. 03, 2021).

[21] “CompTIA A+ Cert Guide: Power Supplies and System Cooling | Foundation Topics | Pearson IT Certification.” https://www.pearsonitcertification.com/articles/article.aspx?p=1945640 (accessed Jun. 03, 2021).

[22] “Creality CR-10S Motherboard v2.0,” *Digitmakers.ca*. https://www.digitmakers.ca/products/creality-cr-10s-mother-board-v2-0 (accessed Jun. 03, 2021).

[23] “RAMPS 1.4 - RepRap.” https://reprap.org/wiki/RAMPS_1.4 (accessed Jun. 03, 2021).

[24] “Arduino Mega Pololu Shield - RepRap.” https://reprap.org/wiki/Arduino_Mega_Pololu_Shield (accessed Jun. 03, 2021).

[25] Thingiverse.com, “Fan Adapter 92 / 120 by jgrzybowski.” https://www.thingiverse.com/thing:3940100 (accessed May 19, 2021).

[26] Thingiverse.com, “PC Case Fan Adapter - 80mm to 120mm by DavidHanwell.” https://www.thingiverse.com/thing:131469 (accessed May 19, 2021).

[27] Thingiverse.com, “SSR, Thingiverse.” https://www.thingiverse.com/search?q=SSR+cover&type=things&sort=relevant (accessed May 22, 2021).

[28] “Klipper3d Homepage,” *klipper*. https://www.klipper3d.org/ (accessed May 04, 2021).

[29] C. Bassett, *cadriel/fluidd*. 2021. Accessed: May 04, 2021. [Online]. Available: https://github.com/cadriel/fluidd

[30] “FluiddPI,” *Fluidd*. https://docs.fluidd.xyz/installation/fluiddpi (accessed May 04, 2021).

[31] “Klipper Installation Instructions,” *GitHub*. https://github.com/KevinOConnor/klipper (accessed May 04, 2021).

[32] “Klipper Configuration,” *GitHub*. https://github.com/KevinOConnor/klipper/blob/master/docs/Config_Reference.md (accessed May 04, 2021).

[33] “Getting Started with Arduino products.” https://www.arduino.cc/en/Guide (accessed May 22, 2021).

[34] “Klipper Config Checks,” *GitHub*. https://github.com/KevinOConnor/klipper (accessed May 04, 2021).

[35] “Klipper Manual Bed Level,” *GitHub*. https://github.com/KevinOConnor/klipper/blob/master/docs/Manual_Level.md (accessed May 04, 2021).

[36] “Klipper Bed Mesh,” *GitHub*. https://github.com/KevinOConnor/klipper/blob/master/docs/Bed_Mesh.md (accessed May 04, 2021).

[37] “Resonance Compensation,” *klipper*. https://www.klipper3d.org/Resonance_Compensation.html (accessed May 04, 2021).

[38] “Measuring Resonances,” *klipper*. https://www.klipper3d.org/Measuring_Resonances.html (accessed May 04, 2021).

[39] “Pressure Advance,” *GitHub*. https://github.com/KevinOConnor/klipper/blob/master/docs/Pressure_Advance.md (accessed May 04, 2021)

[40] “Klipper Rotation Distance,” *GitHub*. https://github.com/KevinOConnor/klipper/blob/master/docs/Rotation_Distance.md (accessed May 04, 2021).

[41] Singh K. (Jun. 2018). Experimental study to prevent the warping of 3D models in fused deposition modeling. Int J Plast Technol.

[42] “PolyMideTM PA6-CF”, Polymaker EU, (accessed Nov. 12 2020 2020) https://eu.polymaker.com/product/polymide-pa6-cf/

