# Supplementary material for: High-performance polymer 3D printing – Open-source liquid cooled scalable printer design
Source: HardwareX. 2022 Jan 10;11:e00265. doi: 10.1016/j.ohx.2022.e00265 (PMC9058719; doi:10.1016/j.ohx.2022.e00265)
Supplement: Supplementary data 1 [file mmc1.docx]

“High-performance polymer 3D printing – open-source liquid cooled scalable printer design”, Mendeley Data, V1, doi: 10.17632/7sjjmr9bz2.1

Doi.org/10.17632/7sjjmr9bz2.1
